# High-Throughput Determination of Exchange Rates of Unmodified and PTM-Containing Peptides Using HX-MS

**DOI:** 10.1016/j.mcpro.2025.100904

**Published:** 2025-01-07

**Authors:** Jamie A. Moroco, Alvaro Sebastian Vaca Jacome, Pierre Michel Jean Beltran, Andrew Reiter, Charlie Mundorff, Miklos Guttman, Jeff Morrow, Stephen Coales, Leland Mayne, Yoshitomo Hamuro, Steven A. Carr, Malvina Papanastasiou

**Affiliations:** 1 Broad Institute of MIT & Harvard, Cambridge, Massachusetts, USA; 2 Department of Medicinal Chemistry, University of Washington, Seattle, Washington, USA; 3 Trajan Scientific and Medical, Morrisville, North Carolina, USA; 4 Department of Biochemistry and Biophysics, Perelman School of Medicine at the University of Pennsylvania, Philadelphia, Massachusetts, USA; 5 Janssen Research and Development, Spring House, Pennsylvania, USA

**Keywords:** hydrogen exchange, mass spectrometry, cell digest, exchange rates, acetylation, phosphorylation

## Abstract

Despite the widespread use of MS for hydrogen/deuterium exchange measurements, no systematic, large-scale study has been conducted to compare the observed exchange rates in protein-derived, unstructured peptides measured by MS to the predicted exchange rates calculated from NMR-derived values and how neighboring residues and post-translational modifications influence those exchange rates. In this study, we sought to test the accuracy of predicted values by performing hydrogen exchange measurements on whole cell digests to generate an unbiased dataset of 563 unique peptides derived from naturally occurring protein sequences. A remarkable 97% of observed exchange rates of peptides are within two-fold of predicted values. Using fully deuterated controls, we found that for approximately 50% of the peptides, the amino acid sequence and, consequently, the intrinsic exchange rate, are the primary contributors to back exchange. A meta-analysis of the remaining physicochemical properties of peptides revealed multiple features that contribute either positively or negatively to back exchange discrepancies. Employing our workflow for comparable measurements on synthetic peptide mixtures containing post-translational modifications, and their unmodified counterparts, we show that lysine acetylation has a strong effect on the observed exchange rate, whereas serine/threonine phosphorylation does not. Our automated workflow enables high-throughput determination of exchange rates in complex biological peptide mixtures with diverse properties.

## Highlights

• Novel high-throughput HX-MS workflow to calculate *k*_*obs*_ across hundreds of peptides.
• Majority of *k*_*obs*_ falls within a 2-fold range of the *k*_*calc*_ predicted values.
• Peptide physicochemical traits strongly influence back exchange rates.
• Acetylation significantly affects *k*_*obs*_ while phosphorylation has minimal impact.
• FD controls are essential for comparing modified and unmodified peptides.

## Graphical Abstract

**Figure:**
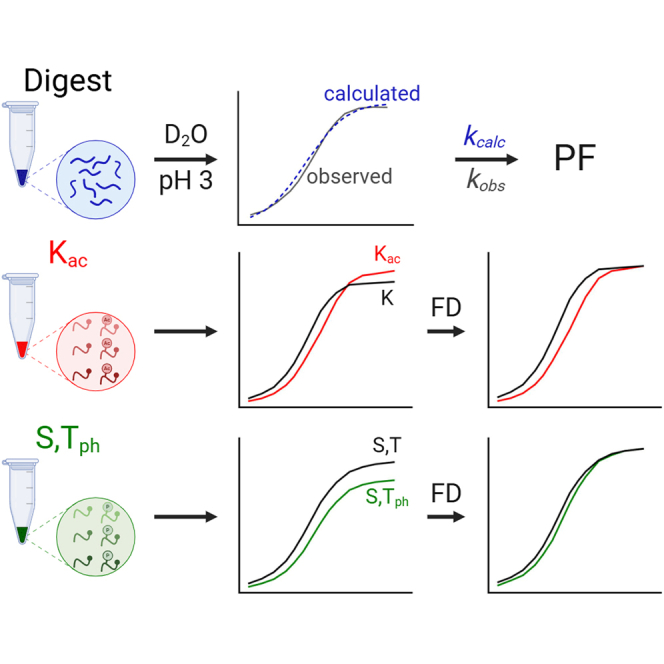

## In Brief

This study provides the first large-scale comparison of NMR-derived hydrogen exchange rates to MS-derived values, analyzing more than 500 peptides. We show that peptide properties beyond amino acid sequence significantly influence back exchange during analysis. Additionally, we demonstrate that lysine acetylation, but not serine/threonine phosphorylation, substantially affects observed exchange rates. These findings establish mass spectrometry as a reliable tool for high-throughput exchange rate determination while highlighting important considerations for hydrogen exchange mass spectrometry experiments.

The exchange rates for individual amide hydrogens to deuteriums were established nearly 30 years ago by measuring the exchange of model dipeptides by NMR. Hydrogen exchange (HX) rates for unstructured peptides were calibrated as a function of pH, temperature, nearest neighbor effects, salt concentration, and isotope effects (1, 2), with minor adjustments reported more recently (3). Improvements in mass spectrometry (MS) sensitivity and resolution have enabled HX-MS to become an invaluable tool for studying protein dynamics, folding, energetics, conformational changes, and interactions in a wide variety of biological systems (4, 5). The majority of these studies use a bottom-up approach in which the labeled protein is digested into peptides using a protease or combination of proteases, and deuterium incorporation is measured at the peptide or near amino acid level (6, 7, 8, 9). Typically, the subsequent analysis is qualitative, where D-uptake values of peptides in the free-protein state are compared to D-uptake values of respective peptides in a bound-protein state, frequently resulting in the localization of perturbation-induced structural and dynamic changes (10, 11, 12). Less often, D-uptake values derived from MS are correlated with quantitative biophysical values, such as average protection factors (PFs) and folding free energies (ΔG) (13, 14). These values are obtained by comparing the observed D-uptake by fitting to a single, double, or stretched single exponential (3, 15, 16, 17) and compared with respective calculated D-uptake of unstructured peptides (1, 2, 3). To the best of our knowledge, a systematic, large-scale study investigating how neighboring residues or post-translational modifications (PTMs) influence the observed rate of exchange (*k*_*obs*_) in unstructured peptides derived from native protein sequences has yet to be explored.

Here, we develop a robust, high-throughput method using a typical six-minute gradient employed for HX-MS experiments to enable the labeling and acquisition of hundreds of peptides from Jurkat cells, yielding approximately 50,000 manually validated isotopic distributions. We compare MS-measured *k*_*obs*_ to NMR-derived *k*_*calc*_ to identify peptide physicochemical properties that induce deviations. Further, we analyze mixtures of acetylated and phosphorylated peptides to investigate the effect of these post-translational modifications on the observed exchange rates, an effect previously overlooked in the HX-MS field.

## Experimental Procedures

### Chemicals and Reagents

All chemicals and reagents used were purchased from Sigma, unless otherwise noted. Phosphorylated (SPT-PTM-POOL-STphospho-1, PTM-Kit 54) and acetylated (SPT-PTM-POOL-Kac-1, PTM-Kit-37) peptide mixtures, as well as their unmodified counterparts (SPT-PTM-POOL-ST-Unmod-1, PTM-Kit 53; SPT-PTM-POOL-K-Unmod-1, PTM-Kit 33), were purchased from JPT Peptide Technologies GmbH (Berlin). Deuterium oxide (D_2_O, 99.9 atom % D; 151,882), acetonitrile (ACN, 99.9%; A998), and formic acid (FA, 99.5% purity; A117) were from Fisher and HPLC water from J.T.Baker (JT4218–3).

### Sample Preparation

Jurkat cells were grown, lysed, and digested as previously described (18). Briefly, Jurkat cells (clone E6-1 obtained from ATCC (TIB-152)) were lysed for 30 min with ice-cold urea (8 M) lysis buffer, NaCl (75 mM), Tris-HCl (50 mM, pH 8.0), EDTA (1 mM), Aprotinin (2 μg/ml, Sigma), Leupeptin (10 μg/ml Roche), and PMSF (1 mM, Sigma). Samples were spun at 20,000*g* for 10 min, and protein concentration was measured by a BCA assay (Thermo Fisher). Proteins (8 mg/ml) were reduced with dithiothreitol (5 mM, Thermo Fisher) and alkylated in the dark using iodoacetamide (10 mM) following standard protocols. Lysates were diluted to a final urea concentration of 2 M. Samples were digested with LysC (Wako, 1:50 enzyme-to-substrate ratio) at 25  °C for 2 h, followed by Trypsin (Promega, 1:50 enzyme-to-substrate ratio) at 25  °C overnight. The digestion was quenched using a final 1% (v/v) formic acid. Samples were spun at 5000*g* for 5 min to remove precipitated urea and desalted using Sep-Pak C18 columns (Waters, 500 mg WAT043395) following standard protocol. Eluted peptides were dried down and resuspended in 0.23% FA, quantified using a BCA assay (Thermo Fisher), and their concentration was adjusted to a final concentration of 250 ng/μl.

### MS Peptide Identification

For identification runs, peptides (1 μg) were injected using a Chronect HDX Extended Parallel with lipid filtration capabilities (Trajan Scientific and Medical) coupled with a Q-Exactive HF Orbitrap MS (Thermo). Peptides were trapped online for 1 min at 100 μl/min onto an Acclaim PepMap 300 μ-Precolumn (C18, 1 × 15 mm, 163,593, Thermo) using solvent A (0.23% formic acid (v/v)). Peptides were separated onto a Hypersil Gold C18 (1 × 50 mm, 1.9 μM, 25,002–051030, Thermo) at 40 μl/min using solvents A and B (0.23% v/v formic acid in acetonitrile). The following gradient was applied: 2% B to 10% B in 0.1 min, to 35% B in 6 min, to 95% B in 0.1 min; kept at 95% B for 1.6 min and returned to initial conditions in 0.2 min. Both the trap and analytical column were operated at 0 °C. Samples were analyzed in data-dependent analysis (DDA) mode using a Top-15 method. Ion source parameters were: spray voltage 3.8 kV, capillary temperature 320 °C. Full MS scans were acquired in the *m/z* range 300 to 1300, with an AGC target 1e6, maximum IT 100 ms, and resolution (at *m/z* 200) 45,000. MS/MS parameters were as follows: AGC target 5e4, maximum IT 100 ms, isolation window 2 *m/z*, isolation offset 0.5 *m/z*, NCE 28, resolution (at *m/z* 200) 30,000 and dynamic first mass. Unassigned and singly charged ions were excluded from MS/MS. Data were acquired in profile mode in both MS and MS/MS scans. Peptides were identified using SpectrumMill Proteomics Workbench (prerelease version BI.07.09.215, Agilent Technologies). Spectra were searched against the UniProt database containing 69,062 sequences (release date December 28, 2017), including a set of common contaminant proteins (264 sequences). Spectra were allowed ±20 ppm mass tolerance for precursor and product ions, 40% minimum matched peak intensity, and “trypsin allow P” enzyme specificity with up to four missed cleavages. The Instrument was ESI QExactive HCD. Carbamidomethylation was added as a fixed modification and the search mode was set to Identity. For post-translational modification searches, the search mode was set to Variable modifications. Phosphorylated peptide searches allowed variable modifications were Phosphorylated (S,T) using a precursor MH+ shift range of 0 to 90 Da. For acetylated peptide searches, allowed variable modifications were Acetyl (ProtN-term, K) using a precursor MH+ shift range of 0 to 50 Da. A target–decoy FDR of 1% was used for all searches. Peptide Spectrum Match exports were used to generate the FASTA files imported into HDExaminer v3.4.0 (Sierra Analytics) for downstream processing of the data.

### HX-MS Labeling and Analysis

For hydrogen exchange (HX) experiments, Jurkat digests were labeled at 20 °C. The autosampler is equipped with eight temperature-controlled units for the syringe park stations, the labeling, quenching, and filtration modules, the digestion column, and the desalting and analytical columns, which allowed for tightly temperature-controlled HX sample preparation and subsequent chromatographic analysis, resulting in accurate D-uptake measurements. All syringes used for sample preparation and quenching were at room temperature. Peptide digests (3 μl of 0.25 μg/μl in 0.23% FA) were mixed with D_2_O (22 μl, final D_2_O content 88% v/v) and labeled at pH 3 (pH_READ_ 3). Samples were quenched at 18, 30, 60, 120, 180, 300, 600, 1200, 1800, 3600, 7200, 14,400, and 93,600 s with 0.23% FA (40 μl, final pH 2.2). For peptide labeling under denaturing conditions, peptides (3 μl in 0.5 M guanidine hydrochloride (GndHCl), pH 3) were mixed with deuterated GndHCl (22 μl, 0.5 M, pH 3) and quenched with GndHCl (40 μl, 0.5 M, 3.2% FA, final pH 2.3) at 0 °C. Samples were injected in the loop (50 μl, injection amount 500 ng), trapped online, and separated on the analytical column as described earlier. Full deuteration (FD) controls were prepared by labeling for 24 h at 37°C using an EppendorfTM ThermomixerTM R (Thermo Scientific). FD controls were transferred to the autosampler and equilibrated at 20 °C prior to quenching as described above. Undeuterated samples were prepared using protiated 0.23% FA or GndHCl (0.5 M, pH 3). For HX measurements of the post-translationally modified peptides, phosphorylated and acetylated peptide mixtures (10 pmol/peptide, 15 μg total peptide amount), were reconstituted in 3% acetonitrile (0.23% FA) at a final concentration of 100 ng/μl (injection amount 200 ng). These were mixed with their respective unmodified counterparts at a 1:1 (w/w) ratio and labeled as described above. Quench time points and all other parameters were the same as described earlier for the Jurkat digests, including the preparation of undeuterated samples and FD controls. All samples were run in triplicates and timepoints were randomized to eliminate the possibility of biased D_2_O measurements. HX data measurements occurred in MS1 using the Full MS parameters described in MS peptide identification. For calculations of D_2_O uptake, RAW files were imported into HDExaminer v3.4.0, using 88% D_2_O content. Carbamidomethyl of cysteine was added as a global modification; phosphorylation (S,T) and acetylation (Nterm, K) were added as point modifications where appropriate. Analysis occurred assuming that the first 1 residue of a peptide can’t hold deuteration. The number of deuteriums measured was not corrected for back exchange. Theoretical uptake curves were calculated at pH_READ_ 3 and temperature 20 °C. Upon manual validation, data were exported using the Export Peptide Pool Results feature of the software and analyzed further as described below.

### Experimental Design and Statistical Rationale

For hydrogen exchange (HX) measurements, Jurkat peptides were labeled with deuterium across 13 independent time points (18, 30, 60, 120, 180, 300, 600, 1200, 1800, 3600, 7200, 14,400, 93,600 s) to fit the wide range of an exponential curve. The D-uptake of each peptide was normalized to the respective full deuteration (FD) control, prepared by labeling Jurkat peptides for 24 h at 37 °C. Samples and FD controls were independently labeled and analyzed as processing triplicates, using a single Jurkat lysate. Given the scope of this experiment for calculating exchange rates, biological replicates were not necessary. Data at each time point are presented with confidence intervals calculated using Welch’s unequal variances *t* test and by taking into account all of the replicates available for the best charge state (charge state with the most complete set of replicates). Statistically significant differences between D-uptake of unmodified and PTM peptides are calculated using Welch’s unequal variances *t* test at 95% confidence level (*p*-value <0.01) by taking into account processing triplicates within each time point at each state.

### Calculation of Theoretical Rates of Exchange and Protection Factors

Theoretical (pH_READ_ 3, 20 °C) and experimental D-uptake values for all peptides were exported from HDExaminer v3.4.0 without any corrections for back exchange and by considering loss of 1 Da from the N-terminus that cannot hold deuterium. Uptake values were plotted for all timepoints. To allow for a direct comparison of the calculated to measured curves, theoretical D-uptake values were normalized to the theoretical maximum number of exchangeable amides (MaxD = #AAs - # Pro - 1) and measured D-uptake values to the respective experimental FD control values so that all curves converge at 100% deuterium. We fitted experimental data to stretched single exponentials (*D*_*t*_ = *N*(1-exp(−(*k*_*obs*_t)^*β*^))) with *β* = 0.8 (1, 2, 3, 15), where D_t_ is the number of deuterons and N is the number of exchangeable amide sites in a given peptide, and calculated exchange rates (*k*_*obs*_). We compared measured exchange rates (*k*_*obs*_) with the respective calculated HX curves obtained for unstructured peptides (*k*_*calc*_) (1, 2) and calculated Protection Factors (PF = *k*_*calc*_*/k*_*obs*_).

### Data Analysis of Experimental *vs.* Theoretical Measurements

Experimental and theoretical D-uptake values were normalized by dividing D-uptake values by the maximum D-uptake value of the individual peptides. Differences between experimental and theoretical D-uptake curves were calculated using both the root sum of squares deviations (RSSD) and the difference between the areas under the curve (AUC difference; AUC_diff_). The root sum of square deviations was calculated using the following equation RSSD = √[Σ(D_t,i_ - D_e,i_)^2^] where D_t,i_ represents the theoretical D-uptake value at time point i and D_e,i_ represents the experimental D-uptake value at the corresponding time point i. Σ denotes the sum across all time points (t_1_...t_n_) in the dataset. The squared terms ensure that both positive and negative deviations contribute positively to the final score, while the square root operation returns the result to the original unit of measurement (D). AUC was calculated with the *auc* function from the *MESS* package in R 4.1.0. Both linear regression and robust linear regression were calculated using the model (AUC_diff_ ∼ amino_acid_proportion) with the *lm* function from base R and *rlm* function from the *MASS* package (R v4.1.0).

### Back Exchange Corrections

Back exchange values were calculated based on experimental FD controls (FD_exp_) and the theoretical maximum number of exchangeable amides (MaxD = #AAs - # Pro - 1) using the following equation: BE = 1-(FD_exp_/Max D). BE corrections of individual peptides occurred using the D-retention values as reported in (19), based on the amino acid composition of the peptide at its N-terminus, its C-terminus and at each position, taking into account neighboring residues (1, 2, 3). To determine the physico-chemical properties of individual peptides, we first used the *Peptides* package in R (v. 2.4.4) and manually added additional attributes. We then performed a feature selection to classify each physico-chemical property based on its importance, using a variable importance measure (VIM). For this analysis, we employed the *Boruta* package in R (v. 7.0.0) which uses Random Forest (20). This algorithm performs a top-down search for relevant features by comparing original attributes' importance with the importance achievable at random, estimated using their permuted copies, and progressively eliminating irrelevant features to stabilize that test.

### ETD Measurements of Unmodified and Acetylated Peptides at Single-Residue Resolution

Fully labeled controls were prepared and quenched as described above. Samples were thawed at 5°C for 8 min and injected using a custom LEAP robot integrated with an LC-MS system 1. The protein was loaded at 400 μl/min over a Waters XSelect CSH C18 trap cartridge column (2.1 × 5 mm 2.5 μm) and resolved over a CSH C18 column (1 × 50 mm 1.7 μm 130 Å) using linear gradient of 5 to 35% B (A: 0.1% FA, 0.025% TFA, 5% ACN; B: ACN with 0.1% FA at 80 μl/min) over 10 min. Eluting peptides were analyzed on an Orbitrap Ascend (Thermo). A series of washes over the trap column were used between injections to minimize carry-over (21).

Non-deuterated samples were used for data-dependent acquisition (DDA) using both HCD and CID fragmentation. Peptides were identified using Byonic (Protein Metrics). For deuterium measurements, targeted LC-MS was employed for each peptide of interest using a 0.8-min retention time window at their elution time. To account for the mass shift from deuteration, the masses for the 3+ charge state were targeted using a 6 *m/z* window centered 2 *m/z* higher than the monoisotopic mass. The H-ESI ion source voltage was 3500 V and the ion transfer tube temperature was 250 °C. The MS method involved two distinct scan experiments. First, a Full MS scan was conducted at a resolution of 60,000 in the Orbitrap, covering a scan range from 350 to 2000 *m/z*. The AGC target was set at 250%, the RF Lens was 60%, and maximum fill time 123 ms. All data were collected in profile mode. Second, a targeted MS2 scan was performed at a resolution of 30,000 in the Orbitrap, utilizing the quadrupole with a 6 *m/z* isolation window. The AGC target for this scan was 300%, the RF Lens was 30%, maximum fill time 200 ms, and using calibrated charge-dependent ETD parameters. Spectra for both intact peptides and c/z ions were exported from Thermo Xcalibur and deuterium content was analyzed using HX Express (22, 23).

## Results

### High-Throughput HX-MS Measurements of a Jurkat Cell Digest

In this study, we employed the latest addition to the HX automation arsenal, the Chronect HDX Extended Parallel with lipid filtration capabilities (Trajan Scientific and Medical), to develop and optimize a high-throughput protocol to enable the analysis of enzymatically digested cells. The two arms provided parallel processing capabilities and allowed for the labeling of time points as early as 18 s while minimizing "downtime". Rapid trapping and desalting (1 min) limited the deuterium’s back exchange with water in solution. Using a 6-min gradient, we identified ∼1600 peptides. The desalting and analytical columns were backflushed with a higher organic content (50% ACN) after the elution gradient to eliminate carry-over and, consequently, the requirement for blank runs between samples. To avoid biased deuterium measurements, the labeling time points were randomized, while triplicates ran together. Our optimized method allowed for the labeling and acquisition of 42 samples per day, yielding high-quality, reproducible data.

**Fig. 1:**
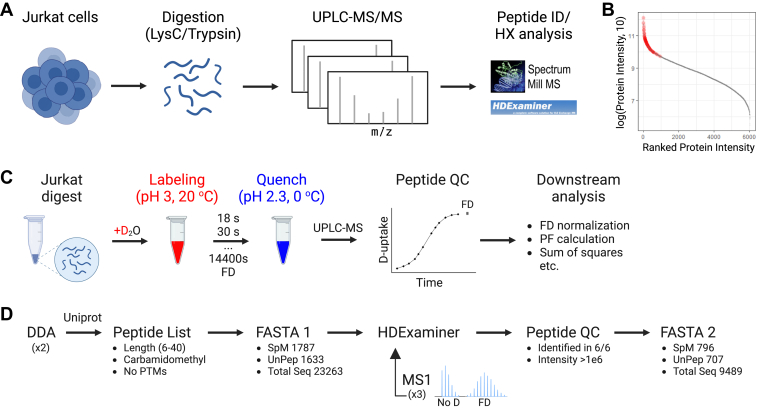
**Experimental and analysis workflow**. *A*, sample generation workflow *B*, intensities of proteins identified with a latest generation nanoLC-MS/MS using a 2-h gradient (*grey*, 6000 proteins) and the HX-UPLC-MS/MS using a 6-min gradient (*red*, 235 proteins). Intensities are ranked from highest to lowest. *C*, Labeling workflow of the Jurkat digest *D*, FASTA generation workflow using non-deuterated and fully deuterated samples.

To enable simultaneous HX-MS measurements of hundreds of peptides, we employed Jurkat cells and generated tryptic digests following a conventional proteomics sample preparation workflow (Fig. 1*A*). Using our fully automated HX-UPLC-MS/MS setup, we were able to identify 1630 peptides that correspond to 235 proteins that were among the most abundant in the cell (Fig. 1*B* and Supplemental Table S1). The number of peptides identified in our study is similar to a recent study that used *E. coli* digestion and sub-zero chromatography with a 90-min gradient to identify 1400 peptides (24). The majority of our peptides were between 6 and 20 amino acids long and hydrophilic, as evident by their grand average hydropathy (GRAVY) score (Supplemental Fig. S1) and mainly measured with +2 and +3 charge states. Despite that the tryptic dataset possesses an inherent bias toward basic C-terminal residues, these peptide properties are comparable to those obtained in HX-MS experiments following digestion with protease XIII (25, 26) and Nepenthesin II (27) which also cleave at basic residues (discussed further in the Discussion section).

Leveraging our developed HX-MS method, we then labeled the tryptic peptides with deuterium (Fig. 1*C*). To decelerate the intrinsic rate of exchange and enable measurements on a typical HX-MS timescale (seconds to hours), we labeled peptides at pH 3 and 20 °C, where, based on poly-DL-alanine reference molecules, the intrinsic rate of exchange is minimal and H-D exchange is still predominantly dominated by base catalysis (1). To account for heterogeneity in rate constants and capture the full range of observed exchange rates, peptides were labeled across 14 timepoints, ranging from 18 s to 26 h, yielding high temporal resolution. Full deuteration (FD) controls were also included upon incubation of peptides for 24 h at 37 °C under the same solution conditions. To ensure equivalent handling with the labeled samples, FD controls were transferred to the autosampler and equilibrated at 20 °C prior to injecting.

HX runs were subsequently imported into HDExaminer for D-uptake analysis and validation. The 1630 peptide sequences were combined into a single file for the generation of the FASTA required for analyzing the HX data, yielding 23,200 amino acids (Fig. 1*D*). To efficiently filter the dataset, we ran triplicates of undeuterated and fully deuterated controls with MS1 detection (as we would for typical HX-MS experiments) and retained only peptides identified in all six runs based on manual inspection of peptide isotopic distributions. Peptides removed were either due to the low signal-to-noise of the wider *m/z* distribution observed compared to the corresponding natural isotopic distribution or due to spectral interferences detected in the shifted mass. Following this process, our dataset consisted of 707 distinct peptides, comprising approximately 9500 amino acids.

**Fig. 2:**
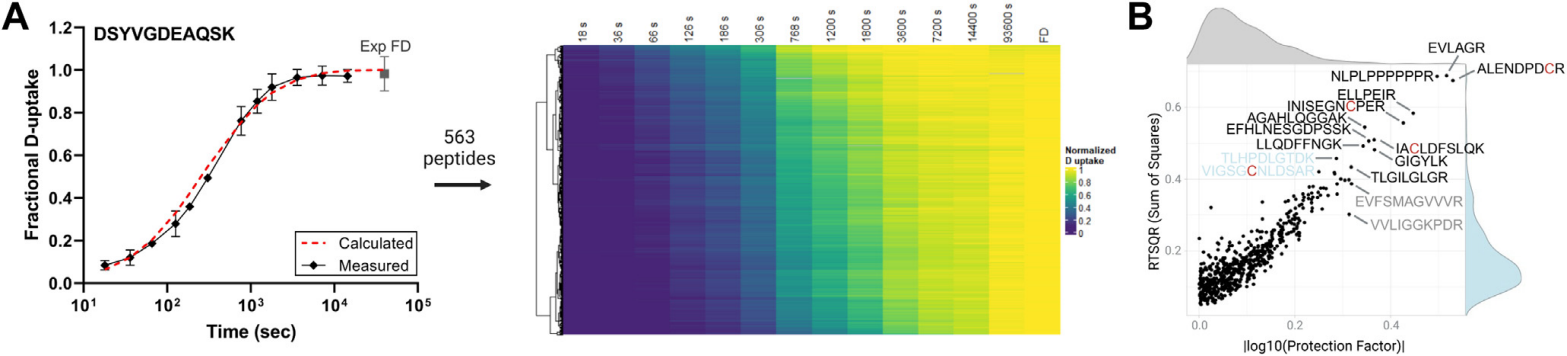
**Comparisons between calculated and measured exchange rates**. *A*, example D-uptake plot of DSYVGDEAQSK (+2) and heatmap containing all peptides that passed quality control in our dataset, ranked by the median value per peptide. Confidence intervals are drawn by calculating Welch’s unequal variances *t-test* using all of the replicates available for the best charge state of the peptide. *B*, distributions of the different methods (absolute values log10 transformed protection factors and root sum of square deviations, RSSD) used to compare calculated and observed curves and respective scatter plots (see Experimental Procedures for further details). Strong positive correlations between the absolute log10 PFs and RSSDs were calculated using Pearson (R = 0.92) and Spearman (ρ = 0.82) correlations. Sequences of outlier peptides with PF and RSSD values outside the 97.5 percentile are shown. Sequences of outlier peptides detected with both methods are shown in *black*. Sequences of outliers detected only using PF and RSSD values are shown in *grey* and *light blue* respectively. Carbamidomethylated cysteines are colored *red*.

The entire dataset was comprised of more than 30,000 isotopic distributions that were manually inspected to determine their D-content. If needed, chromatographic elution profiles were reintegrated, and the D-content was reanalyzed. Excluded from subsequent analysis were peptides with missing time points or co-eluting spectra that rendered D-uptake measurements ambiguous. Thus, complete deuterium exchange curves were obtained for 563 out of the 707 unique peptides. A global view of the data is depicted in the form of a heat map in Figure 2*A* and Supplemental Table S2. Deuterium incorporation values for all peptides in our dataset, followed the expected trend, with low values at early time points and high values at later time points, with peptides reaching equilibrium at different time points, yielding a wide range of *k*_*obs*_.

### Low pH Labeling of the Jurkat Cell Digest Using HX-MS Enables Accurate *K*_*obs*_ Quantification

In HX-MS studies, labeling of a protein typically occurs over a relatively narrow time window (seconds to hours) near neutral pH, relevant to the protein’s function (4, 28, 29). Amides, however, can exchange with rates ranging from msec for unstructured peptides to days or months for stable folds. In these cases, multiple time points spanning a wide time-sampling window are required to enable accurate determination of the exchange rates (14, 29). As such, sampling over a broad temporal range is necessary for detecting significant exchange events that inform protein function and/or stability. In the present study, we performed labeling at low pH (pH_READ_ 3) since our peptides are anticipated to be unstructured, and labeling at a neutral pH resulted in high D-uptake even at very short time points to accurately measure *k*_*obs*_ (data not shown).

The observed D-uptake curves for peptides detected in our dataset display a broad range of exchange rates (*k*_*obs*_). These curves were compared to uptake curves predicted for unstructured peptides at pH_READ_ 3 (1, 2). Experimental and calculated curves were fitted to stretched single exponentials (see Experimental Procedures), using a stretching factor *β* = 0.8 that falls within the narrow range that has been shown to work well for unprotected peptides (*β* = 0.8 ± 0.1, (3, 15, 17)) and *k*_*obs*_ and *k*_*calc*_ were calculated. These were subsequently used for the calculation of Protection Factors (PF = *k*_*calc*_*/k*_*obs*_). Given that labeling occurs at the peptide level and in an acidic environment following a conventional proteomics sample preparation workflow that includes urea protein denaturation and the use of organic solvents during desalting steps, we anticipate that all our peptides are unstructured and have minimal protection against HX, resulting in PF values close to unity. Peptides had PF values < 3.5 with an average PF (PF_all_) of 0.99 (Supplemental Table S3), confirming the absence of ordered structures (15, 16). Our results indicate a strong correlation between the *k*_*obs*_ derived from our MS measurements and the *k*_*calc*_ derived from NMR. Specifically, ∼97% of the *k*_*obs*_ fall within a 2-fold range of the predicted values, providing additional support for the accuracy of the *k*_*calc*_ (Supplemental Fig. S2*A*, (1, 3)). A positive correlation between NMR- and MS-derived exchange rates has previously been demonstrated for small proteins in their native environment (equine cytochrome C and staphylococcal nuclease) labeled at near neutral pH over a wide time-sampling window (13, 29, 30, 31, 32).

To provide a quantitative measure of the discrepancy between observed and calculated exchange curves, we utilized the root sum of square deviations (RSSD) as a complementary analysis to PF values. After normalizing curves to their full deuteration controls (Supplemental Fig. S2*B*), we found that 551 peptides (98% of the dataset) showed RSSDs below 0.2, indicating excellent agreement between observed and calculated curves (Fig. 2*B*). Strong positive correlations between absolute log10 PFs and RSSDs were observed using both Pearson (R = 0.92) and Spearman (ρ = 0.82) correlations, with both methods effectively identifying similar outlier sequences.

Peptides (14 peptides) with PF values outside the standard normal distribution (97.5 percentile) are shown in Figure 2*B*. Three of these peptides contained carbamidomethylated cysteines, a modification introduced during sample preparation. Whether the addition of carbamidomethyl (+57 Da, C_2_H_3_NO) affects the intrinsic exchange rate of a peptide is unknown. In total, we identified a small subset within our dataset (19 peptides) containing 1, 2, or 3 carbamidomethylated cysteines. The average PF for these peptides was larger than the PF_all_, indicating that the presence of carbamidomethyl decelerates *k*_*obs*_ (Supplemental Fig. S2*A*). The remaining 11 peptides lacked characteristics that would indicate extreme exchange behavior (*i.e.*, many slow or many fast-exchanging residues), and their properties (length, hydrophobicity) were well within the range of peptides generated in HX-MS experiments. The exception to this was a peptide with a high proline content (73%, VPPPPPIAR), which had higher deuteration levels than the calculated curve until equilibrium was reached. These findings led us to hypothesize that, for some peptides, the primary amino acid sequence and experimental conditions employed may not be the sole determinants of *k*_*obs*_ and that other peptide properties may be contributing.

In summary, these results show that our introduced HX-MS workflow, which employs peptide digests and labels them under low pH conditions, effectively validates the accuracy of NMR-derived exchange rates. The findings of our study unambiguously demonstrate that HX-MS can reliably generate HX rates in a high-throughput fashion by concurrently measuring deuterium on hundreds of peptides. This method holds significant potential for the analysis of peptide mixtures with diverse characteristics (*e.g.* post-translationally modified peptides) that are difficult to obtain by NMR in a high-throughput fashion.

### FD Controls Provide Novel Insights into Peptide Properties Contributing to Deuterium Retention and Back Exchange

Due to the presence of water in the quench solution and mobile phase, D-label is lost during the quench and LC separation steps in HX-MS studies, a phenomenon known as back exchange (BE). D-loss is inevitable and highly dependent on the intrinsic exchange rate of the peptide residues (1, 33). In addition, variations in the intrinsic amino acid HX rates result in back exchange values that are highly variable from peptide to peptide due to their varying lengths and amino acid compositions. In typical bottom-up HX-MS workflows, the average back exchange level reported in the literature is 30%; however, this number is highly dependent on the sample preparation method, ionic strength, pH, and instrument parameters (34, 35, 36).

**Fig. 3:**
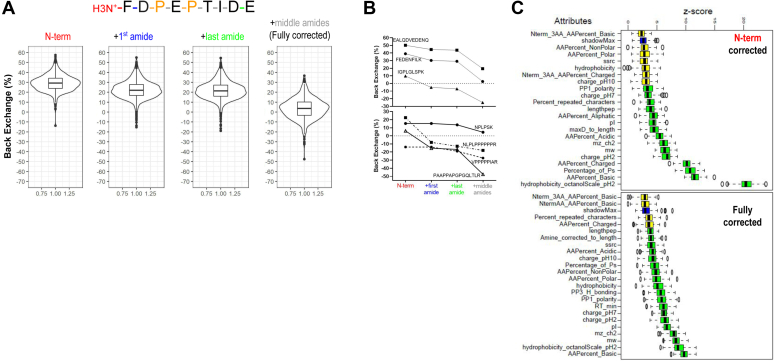
**Back-exchange correction workflow.***A*, sequence cartoon depicting the amide bonds in a peptide and boxplots showing BE values upon correction for D-loss from the N-term, first amide, last amide and middle amides based on Bai, Connely, Nguyen (see Main Text and Experimental Procedures for calculation details). *B*, BE corrections for example peptides (*top*) and proline-rich peptides (*bottom*) *C*, boruta result plots for N-term and Fully corrected BE values. *Yellow* and *green boxplots* represent z-scores of tentative and confirmed attributes, respectively. *Blue boxplots* correspond to the maximum z-score of a shadow attribute.

To evaluate our system and quench conditions for BE, we employed experimental FD controls and calculated BE values (described in detail in Experimental Procedures). We initially considered a loss of 1 Da corresponding to the loss of 1 D at the N-terminus (Fig. 3*A*). Our workflow resulted in most peptides exhibiting a median back exchange rate of 29%, which is within the expected range. Intriguingly, BE values ranged from as low as 3% to as high as 58% (selected peptides shown in Fig. 3*B*, top), with an outlier peptide with a high proline content (VPPPPPIAR) displaying negative BE (−14%, Fig. 3*B*, bottom). Negative BE indicates the retention of a higher number of D than expected and could be attributed to exchange and retention of D by the NH of the guanidine side-chain of Arginine that can occur, especially at low pH (37). To investigate whether such extreme differences are solely the result of the intrinsic exchange rate of a peptide's sequence or if other factors contribute to BE, we corrected uptake values based on the complete amino acid composition of individual peptides (Fig. 3*A*). To achieve this, we sequentially corrected BE values based on the first two amino acids (first amide), the last two amino acids (last amide), and all amino acids in between these amides (middle amides) using values reported in (19) that are based on previous studies (1, 3).

Correcting for the first amide resulted in a 7% decrease in the median BE values (from 29% to 22%), which is expected since the majority of peptides will lose deuterium from the first amide completely, unless one or both residues are Val, Leu and Ile (38). Correcting for the last amide had a negligible effect on the average BE values (21%), as all of our peptides have Lys or Arg at their C-terminus for which the D-retention is greater than 87% (except GK and GR at ∼76%). Correcting for the remaining middle amides had a significant effect on BE values, reducing the median to 4%. Strikingly, only 257 out of 519 peptides fell within the interquartile range, indicating that the experimental values roughly match predicted BE values for only 50% of the peptides. For those peptides, the amino acid sequence and, consequently, the intrinsic exchange rate are the primary contributors to BE. An example is depicted in Figure 3*B* (FEDENFILK, top), for which full BE correction results in 3% BE. Interestingly, the BE values of the remaining peptides were distributed from as low as −50% (*i.e.* PAAPPAPGPGQLTLR) to as high as 37% (*i.e.*, IWHHTFYNELR). For those peptides, it is evident that factors other than the amino acid sequence contribute to BE behavior.

A previous systematic investigation of factors influencing BE revealed an unexpected dependence of back exchange on ionic strength, while decreasing the peptide's residence time in the LC column by employing shorter gradients yielded negligible improvement (35). Given that no salt was used in our study, we focused our analysis on the physicochemical properties of the peptides and their potential correlation with their BE behavior. First, we divided our N-term corrected dataset into four groups based on % BE, with the same number of peptides in each group (Supplemental Fig. S3*A*). To determine whether a peptide property significantly influences BE, we searched 47 attributes (Supplemental Table S4) for significance using the Boruta package, which provides an unbiased and stable selection of important and non-important attributes from a dataset ((20), Experimental Procedures). 15 of these attributes are the most pertinent contributors to a discrepancy in the BE (Fig. 3*C* and Supplemental Fig. S3*B*). Hydrophobicity on the octanol scale (hydrophobicity_OS_), which is a whole-residue scale that includes both sidechains and peptide bonds (39), was the most significant variable with its z-score separated from other attributes. Plotting its median value across all four groups (0.2–0.4) revealed that hydrophobicity_OS_ and the percentage of basic and charged residues (39), strongly correlated with BE values (Supplemental Fig. S3*A*). In contrast, the length, molecular weight, *m/z* and % of proline content of a peptide were all negatively correlated with BE values.

To then investigate the properties of peptides that still had high positive or low negative BE values in the fully corrected dataset, we performed the same analysis, this time dividing the dataset into three groups: one with values close to 0% (peptides in this category reached 0% BE upon full correction and therefore matched theoretical values, *e.g.*, FEDENFILK in Fig. 3*B*), and the other two with positive (under-corrected, *e.g.*, EALQDVEDENQ) or negative (over-corrected, *e.g.*, IGPLGLSPK) values at the extremes (Supplemental Fig. S3*A*). In this analysis, 19 attributes were found to be significant, the majority of which were similar to those identified as significant in the N-term corrected dataset, albeit with lower z-scores (<10 *vs.* ∼20 for the N-term dataset), indicating that differences between variables were more subtle in the fully corrected dataset. Hydrophobicity_OS_ and the percentage of basic amino acids exhibited comparable trends, as in the N-term corrected dataset. Intriguingly, the percentage of charged amino acids was not identified as a significant variable in the fully corrected data set, suggesting that corrections across the entire peptide length account for this variable. Size-related peptide parameters (molecular weight, m/z, retention time, and peptide length) were comparable between groups 1 and 3, but not with group 2, indicating that these are not important contributors to over- (group 1) or under-corrected (group 3) BE values (Supplemental Fig. S3*A*). Finally, group 1 peptides showed a higher percent of non-polar and proline-containing residues compared to the other two groups. Example peptides indicative of these trends are depicted in Figure 3*B*. The peptide EALQDVEDENQ contains a high percentage of polar and charged residues, and in the fully corrected dataset, the BE value is at 19%, indicating faster D-loss than predicted. In juxtaposition, the peptide IGPLGLSPK, which contains a high percentage of small and non-polar residues, exhibits a fully corrected BE value of −25%, indicating that this peptide undergoes a slower D-loss than predicted. All proline-rich peptides result in negative BE values.

To evaluate the potential impact of secondary structure on BE rates, we analyzed the helical propensity of all peptides using AGADIR. This computational tool integrates multiple parameters including peptide sequence, temperature, ionic strength, and pH to predict secondary structure formation (40). Our analysis revealed that all peptides exhibited minimal helical propensity (<2.5%), suggesting the absence of stable helical conformations in solution (Supplemental Table S3). However, previous studies have demonstrated that peptide-column interactions during chromatographic separation can induce secondary structure formation, even in peptides with negligible predicted helical propensity in solution (41). Additional sequence characteristics, such as hydrophobicity and amphipathicity in specific regions of the peptide, may also influence how these structures form on-column. While some peptides in our study may experience such column-induced structural changes, the brief duration between quench and detection (less than 8 min) makes it unlikely that these interactions alone could account for the substantial BE observed in certain peptides. Although we cannot completely exclude solid-phase interactions as a contributing factor to the variations in BE values, their impact is likely secondary to other mechanisms, which is supported by the magnitude and pattern of BE differences observed across our peptide set.

Taken together, this analysis uncovered previously overlooked peptide properties that contribute either positively or negatively to the BE behavior of a peptide. Our analysis identifies specific residue classes that need to be considered in HX-MS studies aiming to investigate protein dynamics, quantify structure, or achieve single-residue resolution, and underline the necessity of revised models to take into account these parameters.

### The Observed Exchange Rate of Peptides is Strongly Affected by Lysine Acetylation but not by Serine/Threonine Phosphorylation

**Fig. 4:**
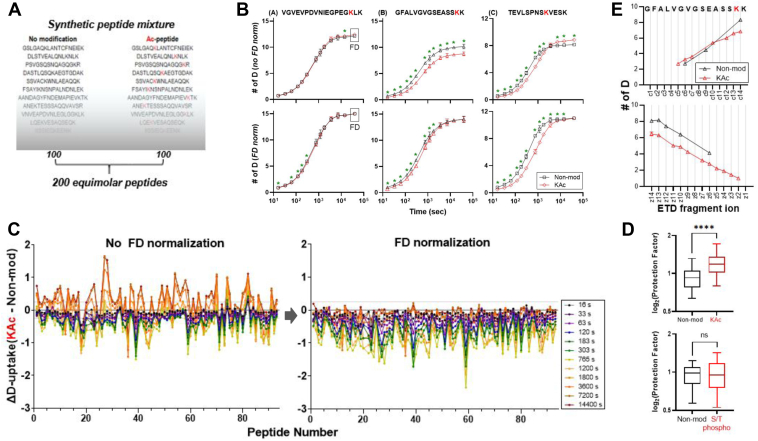
**Effects of K acetylation and S,T phosphorylation on exchange rates**. *A*, example peptide sequences of the non-modified and acetylated peptide pairs *B*, example D-uptake plots for peptides belonging to groups (*A*–*C*), before and after FD corrections; statistically significant differences are marked with an asterisk (Welch’s test). Confidence intervals are drawn by calculating a Welch’s unequal variances *t* test using all the replicates available for the best charge state of the peptide. *C*, Bird’s eye view of D-uptake differences before and after FD corrections. *D*, calculated PF (log2) for the non-modified and acetylated or phosphorylated peptide pairs following FD corrections. Statistically significant differences (paired *t* test) are detected for the acetylated peptide pairs only (*p* < 0.0001). *E*, Site-specific determination of amide exchange kinetics using ETD for GFALVGVGSEASSKK (+3).

Using our high-throughput workflow, we then set out to investigate in a systematic manner how PTMs affect D-measurements, a previously very challenging and time-consuming question to answer in the HX-MS field. First, we investigated lysine acetylation as the addition of an acetyl group to the primary amine of lysine will generate an amide bond that can retain some Ds. Further, the acetyl group may slow base-catalyzed HX reactions as acetylation removes the H-bond between the lysine ammonium group and the neighboring amide O. This is similar to the first amide of a peptide that exchanges significantly faster than the rest of amides, because the first amide hydrogen becomes more acidic due to the H-bonding between the N-terminal ammonium group and the first amide O (19). We combined a mixture of 100 synthetic peptides and their modified counterparts containing a single acetylated lysine into their sequences, resulting in a mixture of 200 peptide sequences (Fig. 4*A*). The peptides ranged in length from 9 to 20 residues, and acetylated lysines were incorporated at various positions along the peptide length (Supplemental Fig. S4*A*), providing a representative pool. All peptides were present at approximately equimolar concentrations in both states, accounting for ion suppression effects. Some peptides contained truncated synthetic impurities lacking the N-terminal amino acid, which were used to further validate the D-uptake measurements of those sequences.

All acetylated peptides eluted at a later retention time and were mostly identified with lower charge states compared to their unmodified counterparts, as acetylation neutralizes the positive charge of lysine (Supplemental Fig. S4, *B* and *C*). In total, we were able to obtain complete D-uptake curves for 94 peptide pairs. Prior to FD corrections, results were clustered into three major groups, with a representative example of each group shown in Figure 4*B*. The groups were: (a) peptides for which acetylation has a minimal effect on D-uptake (7 peptides); (b) peptides for which acetylation results in either lower (18 peptides) or higher (2 peptides) D-uptake for all time points; and (c) peptides for which acetylated peptides have lower D-uptake than their unmodified counterparts at earlier time points and higher D-uptake at longer time points (67 peptides, Supplemental Tables S5, S6, and Supplemental Fig. S5). For some peptides, the two curves intersect as early as 300 s and for others as late as 2 h, displaying a broad range of observed exchange rates.

Undocumented observations in the field indicate that D-retention across different charge states within a peptide can vary, and this can be instrument dependent (42), although a rationale is yet to be established. We observed a similar pattern, with higher charge states within a peptide resulting in lower D. In our dataset, we were able to compare D-uptake values for 65 peptide pairs using the same charge state, unambiguously assigning D-differences between unmodified and modified peptides to the addition of the acetyl group. We were unable to directly attribute differences in the remaining pairs to the addition of the acetyl group as different charge states were used across the two states. To correct BE values resulting from differences in charge states and retention time, while allowing for precise comparisons between the two groups, we next normalized D-uptake values to respective FD controls and selected the optimal charge state within each peptide sequence. For peptides in group (A), in which acetylation affected D-uptake levels minimally, FD normalization had a subtle effect (Fig. 4*B*). The acetylated lysine in these peptide sequences was either at the C-1 or C-2 position. For peptides in group (B), FD normalization resulted in minimal, albeit statistically significant, differences in D-uptake measurements between the unmodified and acetylated pairs. FD normalization for peptides in group (C) revealed that lysine acetylation results in a rather significantly lower D-uptake throughout the time course. A bird's-eye view of the D-uptake differences for all peptides, prior to and after FD normalization, is depicted in Figure 4*C*. It is shown that FD normalization substantially corrects measurements, and lysine acetylation results in slower observed exchange rates of modified peptides compared to their unmodified counterparts and, consequently, statistically significant higher PF values (Fig. 4*D*, top). Notably, D differences between the unmodified and acetylated peptides range up to 2 Da.

To investigate the influence of acetylated lysines on exchange rates of lysine and neighboring residues, we next employed ETD to obtain site-specific data for some of the residues (43) using a fully deuterated peptide mixture. We utilized the P1 peptide (HHHHHHIIKIIK) (44) to optimize parameters as described previously to establish low scrambling conditions (45). For efficient generation of c- and z-type ions, only peptide pairs of non-modified and acetylated peptides with higher charge states (+3) were selected for ETD fragmentation (Supplemental Fig. S4*D*). Unfortunately, the acetyl modification most often drastically altered fragmentation propensities and ultimately only a single peptide pair could be effectively compared with a set of c/z ions. Peptide GFALVGVSEASSK(ac)K for which the FD was higher in the unmodified and lower in the acetylated state (10.2 Da and 8.8 Da respectively, Fig. 4*B*) is shown in Figure 4*E*. The acetylated peptide exhibited nearly complete site-resolution as evidenced by the detection of numerous c- and z-fragment ions. By comparing those to fragment ions of its unmodified counterpart, the c-ions have similar levels of deuterium up through c10 but diverge in the c14 fragment. Correspondingly, all comparable z-ions indicate higher D content in the unmodified peptide. In total, the ETD data reveals the N-terminal half of the peptide has comparable levels of deuterium and the main differences in deuteration are specifically at the acetylated lysine and adjacent residues. While comparable c/z ions were sparse, ETD comparisons of three other unmodified/acetylated peptides showed a similar pattern (Supplemental Fig. S4*D*). In this example, the neutralization of a positive charge through acetylation, which eliminates the H-bonding ability of lysine, appears to accelerate the exchange rate of the proximal amides resulting in higher observed BE. While acetylation may cause other effects in other peptides, as suggested by the differences in maximum D content or observed rates, the consequence of acetylation can potentially affect several aspects of amide exchange leading to significant differences in observed exchange. Overall, in the case of this specific PTM, caution is strongly warranted when making comparisons between exchange profiles of modified and unmodified peptides.

We next investigated how phosphorylation affects D-uptake measurements, given that the phosphoryl group results in the addition of 80 Da to serine, threonine, or tyrosine residues and adds a negative charge to the peptide. Phosphorylated serine/threonine is similar to deprotonated glutamic acid in terms of charge that slightly decelerates base-catalyzed HX reactions, while unmodified serine/threonine slightly accelerates base-catalyzed HX reactions. Similar to the acetylated peptide mixture, we prepared a pool of phosphorylated and unmodified peptides and labeled them at low pH (Supplemental Fig. S4*E*). The mixture was composed of an equal number of phosphorylated serines (50 peptides) and threonines (50 peptides), with the majority of the phosphorylation sites towards the N-terminus (Supplemental Fig. S4*F*). The peptides ranged in length from 8 to 23 residues and were at approximately equimolar concentrations in both states. A marginal shift to earlier retention time was observed for phosphorylated peptides compared to their unmodified counterparts (Supplemental Fig. S4*B*). Applying our stringent quality criteria, we obtained complete D-uptake curves for 29 peptide pairs, mainly losing peptides due to low signal at late timepoints, likely associated with non-specific surface adsorption to the vials over time, a common problem with mass-limited phosphorylated peptides at the low nanogram level (46). Prior to FD corrections, for half of the peptides, phosphorylation resulted in similar D values throughout the time course. For the rest of the peptides, D values were lower or higher than those of their unmodified counterparts (Supplemental Tables S7 and S8). Normalizing deuterium measurements to FD controls revealed that phosphorylation had no effect on D-uptake, resulting in comparable observed exchange rates and, consequently, PFs (Fig. 4*D*, bottom). It is important to note that since these experiments were performed at pH 3, where unmodified S/T residues are neutral and phosphorylated S/T carry a −1 charge due to phosphoric acid's pKa values of 2, seven and 12. Our observation that phosphorylated and unmodified S/T exhibit similar back exchange properties at pH 3 suggests that the −1 charge state minimally impacts hydrogen bonding networks. However, this similarity may not hold at physiological pH 7.0 (typical HX conditions), where phosphorylated S/T exists predominantly in the −2 charge state. The additional negative charge could significantly alter local hydrogen bonding patterns and consequently affect HX behavior. Therefore, it is essential to include accurate FD reference samples in these experiments to make direct comparisons.

## Discussion

In the present study, we employed a peptide digest and performed D-measurements at low pH and high-time resolution so that we could accurately calculate *k*_*obs*_ for 563 unique peptides. Despite a 65% loss from the 1630 identified peptides, our stringent filtering criteria (including removal of peptides with missing timepoints or missed identifications in any undeuterated or FD samples) enabled the most robust analysis of the dataset. Comparing *k*_*obs*_ with the predicted *k*_*calc*_ derived from NMR measurements for hundreds of peptides, we show that a remarkable 97% of *k*_*obs*_ are within a 2-fold range of the predicted values. These results effectively validate the accuracy of NMR-derived exchange rates and establish HX-MS a high-throughput and reliable tool for the calculation of *k*_*obs*_.

Using experimental FD controls and upon fully correcting the D-uptake of each peptide based on its complete sequence we show that only 35% of the peptides reach 0% BE. The BE values for the remaining peptides are either positive (faster D-loss than anticipated, 45% of peptides) or negative (slower D-loss than anticipated, 20% of peptides), suggesting additional factors than the peptide sequence and experimental conditions contributing to BE. A meta-analysis of the peptide physicochemical properties revealed multiple features contributing to these discrepancies, such as hydrophobicity_os_, percentage of basic residues, and peptide size-related properties. Given the inherent bias of the tryptic dataset toward basic C-terminal residues, we would like to note that future investigations would benefit from analyzing peptides with diverse C-terminal amino acid properties to draw more comprehensive conclusions. Several promising approaches could address this, including the implementation of HX-specific enzymes like Neprosin, a selective prolyl endoprotease (47) or the utilization of alternative specific proteases that function at non-acidic pH conditions, such as AspN, GluC, or chymotrypsin, to generate a diverse peptide library with varied C-terminal compositions. These methodological refinements would undoubtedly enhance the breadth and depth of future analyses.

The relationship between measured exchange kinetics and BE requires careful consideration. While our on-exchange kinetics align well with theoretical predictions (97% within 2-fold), the BE levels show greater variance. This may be explained by the different sensitivities of these measurements. Exchange rate constants (*k*_*obs*_) are determined across multiple timepoints, while BE measurements reflect absolute deuteration levels at a single timepoint and are therefore more sensitive to peptide-specific physicochemical properties that affect D retention. Additionally, the experimental conditions differ - on-exchange occurs in D2O solution while BE happens during quenching and chromatographic separation where peptide-specific interactions with the stationary phase may play a larger role (discussed in detail in Sheff *et al* (41) and in the Results section above). These factors may help explain why exchange kinetics can closely match predictions even when BE levels show greater variance.

Our results show that in studies lacking experimental FD controls, BE corrections for N-terminal losses (1-Da) and first amide losses (2-Da), followed by uniform 20 to 30% BE corrections across peptide sets, may produce inaccurate quantitative values and necessitate revised models incorporating these parameters. The large deviation from predicted BE across such a large portion of the peptides examined here further strengthens the notion that subtractive analysis of overlapping peptides should be treated with caution (41). While differential HX-MS experiments between multiple states dominate the field, BE corrections remain critical in several scenarios, particularly when comparing regions with distinct intrinsic exchange rates, such as wild type *versus* mutant proteins. This was recently demonstrated in our work comparing N-terminal deletion mutations in the LSD1 intrinsically disordered region (IDR), where full deuteration controls were essential for normalizing deuterium uptake between variants (48). Additionally, BE corrections are especially crucial for IDRs, which experience pronounced back exchange leading to reduced D-uptake measurements and diminished dynamic range (48, 49). BE corrections are also vital when comparing non-concurrent experiments or determining absolute deuterium levels for structural model validation and thermodynamic calculations, as these corrections account for experimental variations in exchange conditions, enable accurate cross-experimental comparisons, and provide the precise deuterium incorporation measurements needed for deriving protection factors and other biophysical parameters that inform protein structure-function relationships (5, 50).

Further, as the role of PTMs is increasingly appreciated in protein/structure function, we sought to determine whether the presence of common PTMs, such as acetylation and phosphorylation, impact *k*_*obs*_. Using FD controls, we show that lysine acetylation leads to faster BE of lysine and neighboring residues than their unmodified counterparts. The effect of acetylation is similar to the N-terminus inductive effect that results in fast deuterium loss from the first amide, as discussed earlier and in previous studies (1, 19). This observation complicates the interpretation of acetylated regions in studies investigating the impact of acetylation on *k*_*obs*_, and requires the execution of additional controls, beyond FDs. Phosphorylation of serine and threonine residues had a limited impact on the observed exchange rate, but as discussed earlier, may vary depending on experimental conditions and FD controls are essential for these comparisons as well.

Overall, as the first comprehensive analysis of its kind examining complete exchange profiles of unstructured peptides and modified matched pairs, this work provides valuable insight into peptide behavior in HX-MS studies and establishes a foundation for future studies to build upon, ultimately helping refine models that utilize HX-MS data to inform computational protein models.

## Data Availability

The raw mass spectrometry data have been deposited in the public proteomics repository MassIVE and are accessible at ftp://massive.ucsd.edu/v07/MSV000094510/. If requested, also provide the username: MSV000094510.

## Supplemental data

This article contains supplemental data.

## Conflict of interest

The authors declare that they have no conflicts of interest with the contents of this article.
