# Supplementary material for: High-Throughput Determination of Exchange Rates of Unmodified and PTM-Containing Peptides Using HX-MS
Source: Mol Cell Proteomics. 2025 Jan 7;24(2):100904. doi: 10.1016/j.mcpro.2025.100904 (PMC11875167; doi:10.1016/j.mcpro.2025.100904)

Supplemental Data for:

High-throughput determination of exchange rates of unmodified and post-translationally modified peptides using HX-MS

Short title:
High-throughput evaluation of H/D exchange rates

Authors:
Jamie Moroco[1,#], Alvaro Sebastian Vaca Jacome[1,#,$], Pierre Michel Jean Beltran[1,&], Andrew Reiter[1], Charlie Mundorff[2], Miklos Guttman[2], Jeff Morrow[3], Stephen Coales[3], Leland Mayne[4], Yoshitomo Hamuro[5], Steven A. Carr[1,*] & Malvina Papanastasiou[1,*]

Affiliations:
[1]Broad Institute of MIT & Harvard, Cambridge, MA
[2]Department of Medicinal Chemistry, University of Washington, Seattle, WA
[3]Trajan Scientific and Medical, Morrisville, NC
[4]Department of Biochemistry and Biophysics, Perelman School of Medicine at the University of Pennsylvania, Philadelphia, MA
[5]Janssen Research and Development, Spring House, PA

Corresponding author information:
* Corresponding author: malpap@broadinstitute.org; scarr@broad.mit.edu

Other author footnotes:
[#] These authors contributed equally to this work
[$] Current address: BioNTech, Cambridge, MA
[&] Current address: Pfizer, Cambridge, MA

Supplemental Tables:

Table 1: Protein identifications using PAL-Orbitrap HF (Figure 1B)
Table 2: D-uptake values of the cell digest (Figure 2A)
Table 3: Sum of squares, PFs, % Helicity (Figure 2C)
Table 4: List of attributes (peptides package and manually added)
Table 5: D-uptake values of acetylated and non-mod peptides
Table 6: Grouped peptides based on D-uptake of uncorrected data
Table 7: D-uptake values of phoshporylated and non-mod peptides
Table 8: Grouped peptides based on D-uptake of uncorrected data
Table 9: Jurkat peptide identifications using PAL-Orbitrap HF

Supplemental Figures:

Supplemental Figure 1:

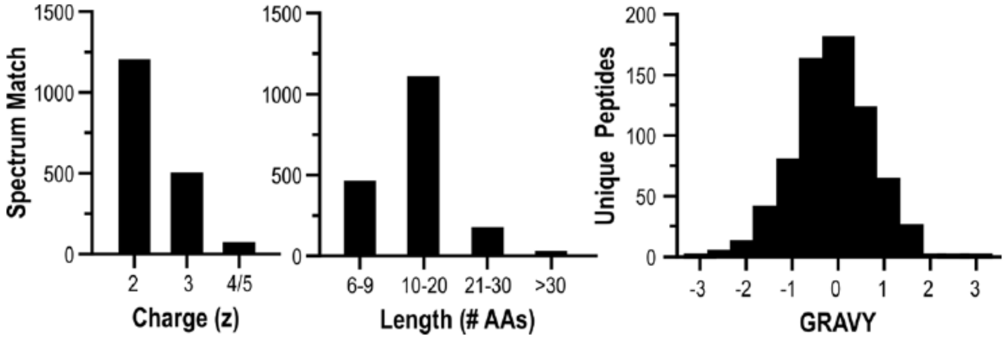

Characteristics of peptides detected from Jurkat cells (charge, length and GRAVY)

Supplemental Figure 2:

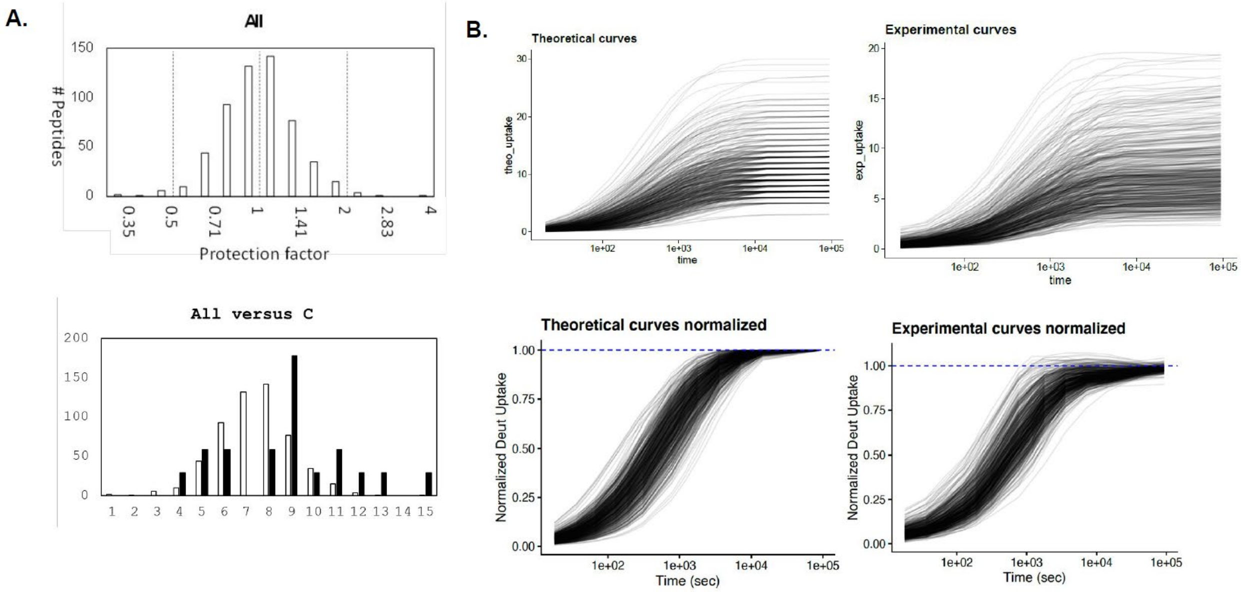

A. Distribution of Protection Factor for all peptides detected in our dataset (563 peptides) and Cysteine-containing peptides (14 peptides).
B. Theoretical and experimental curves before and after normalization to the maximum theoretical number of exchangeable amides (for theoretical curves) and to experimental FD controls (for experimental curves)

Supplemental Figure 3:

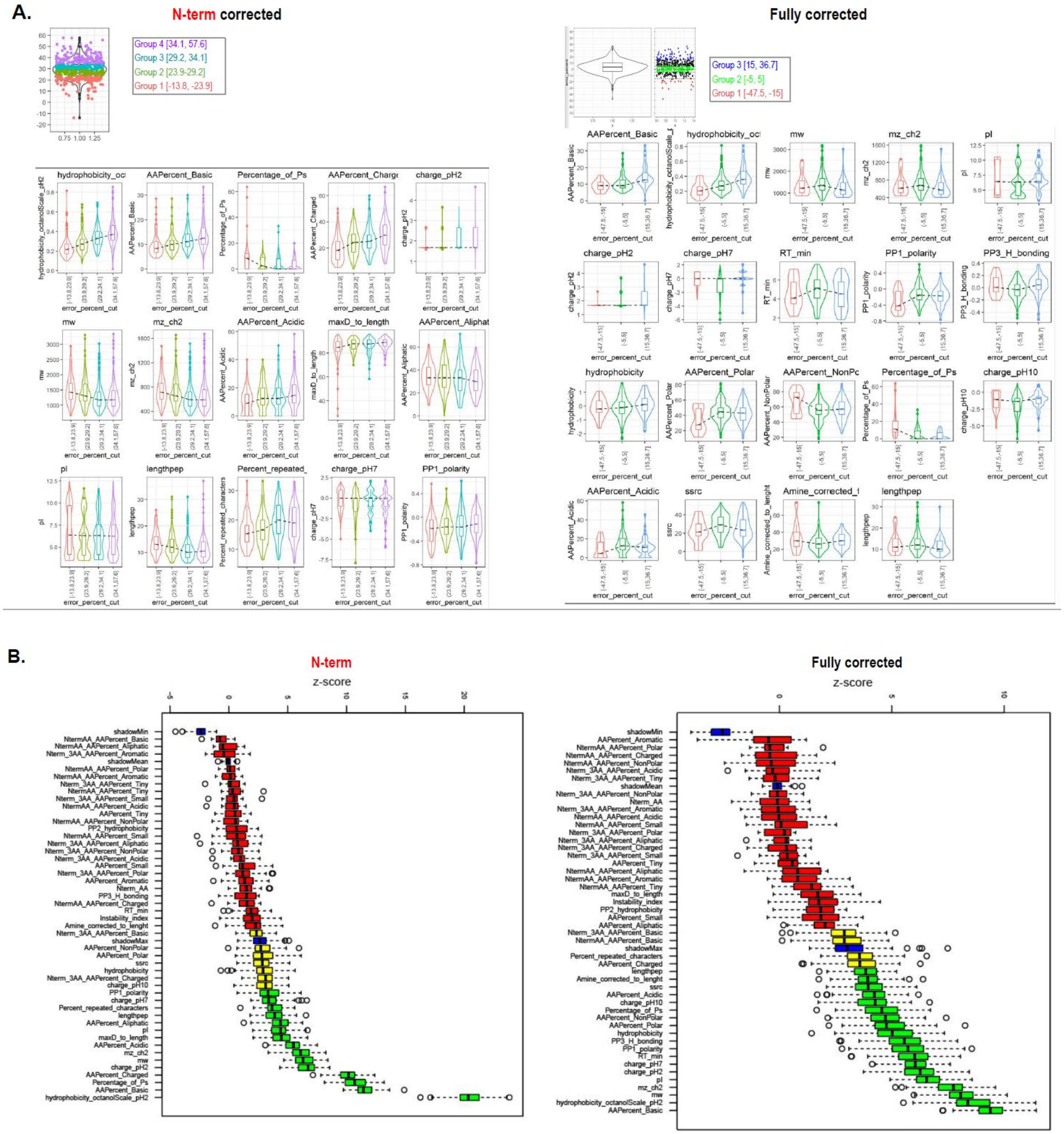

A. Correlation between peptide properties and increasing BE values for the N-term- and Fully-corrected across the full peptide length (see text for details)
B. Full view of Boruta result plots for the N-term- and Fully-corrected BE values

Supplemental Figure 4:

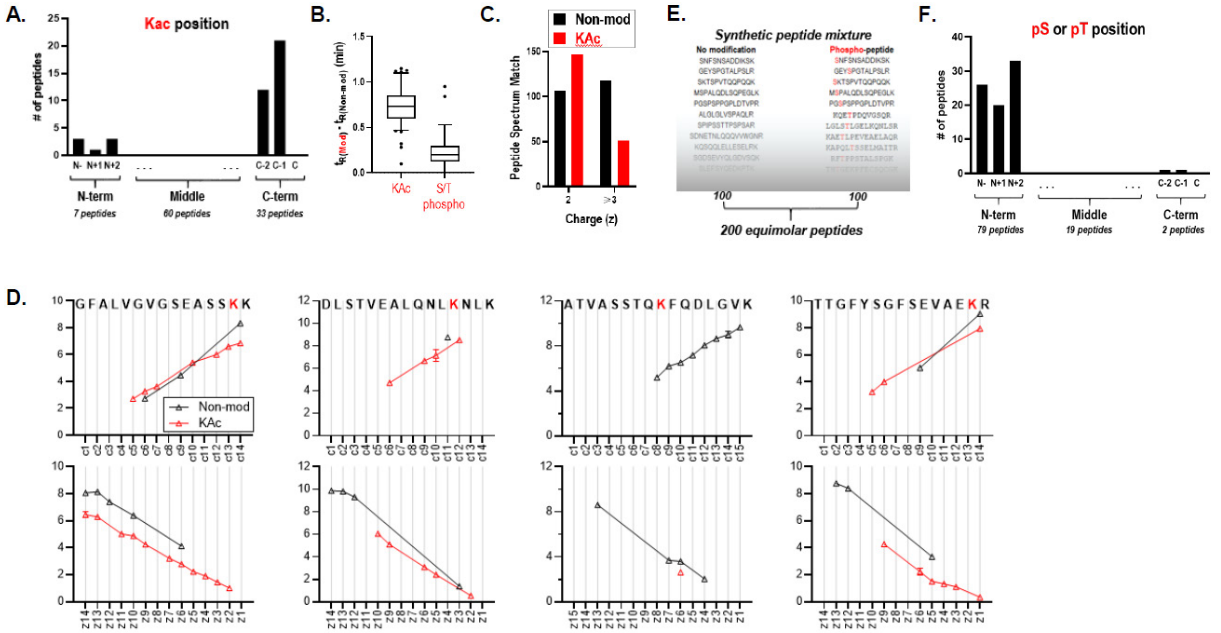

A. Distribution of lysine acetylation across peptide sequences
B. Chromatographic retention time differences between modified and non-modified acetylated (KAc) and phosphorylated (S/T phosphor) peptides
C. Charge state distributions of the non-modified and acetylated peptides (full list of peptide Sequence are appended in Supplemental Table S5)
D. Site-specific determination of amide exchange kinetics using ETD for three peptides. Acetylated lysines are shown in red.
E. Example peptide sequences of the non-modified and phosphorylated peptide pairs
F. Distribution of serine or threonine phosphorylation across peptide sequences

Supplemental Figure 5A: D-uptake plots of non-modified and acetylated peptides (not normalized)

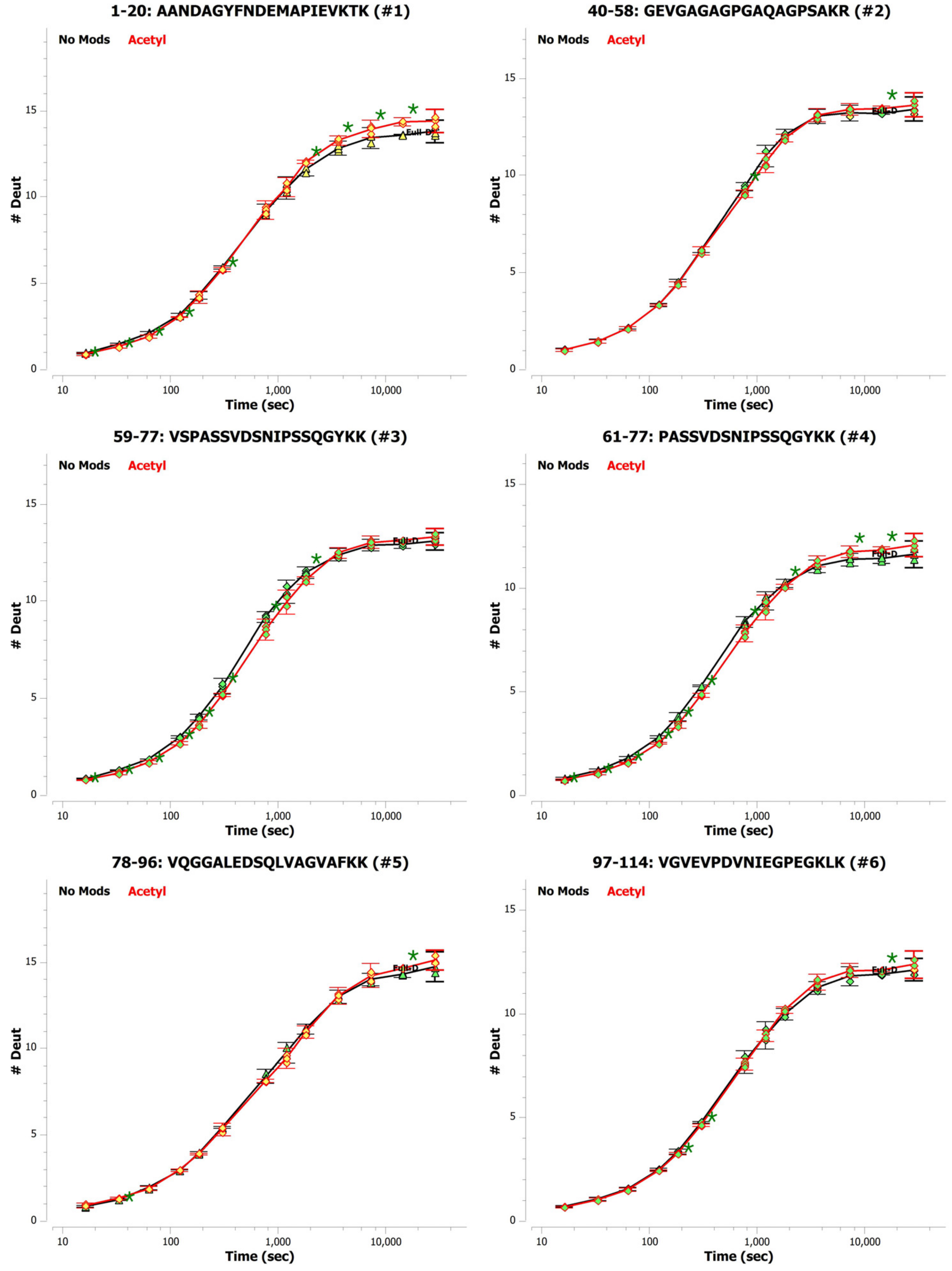

98-114: GVEVPDVNIEGPEGKLK (#7)

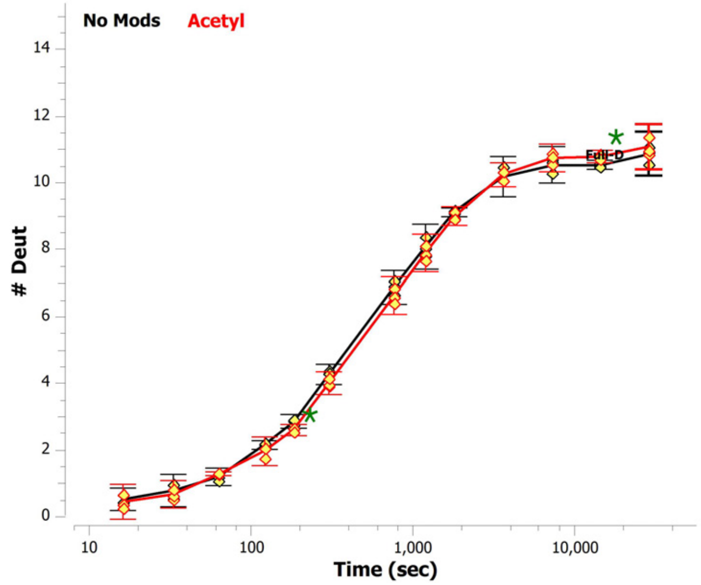

115-132: VDVEVPDVSLEGPEGKLK (#8)

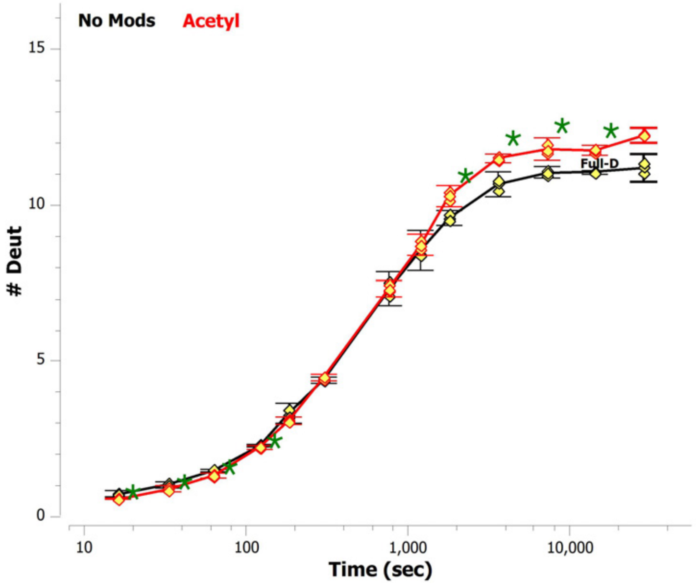

133-150: FSAYIKNSNPALNDNLEK (#9)

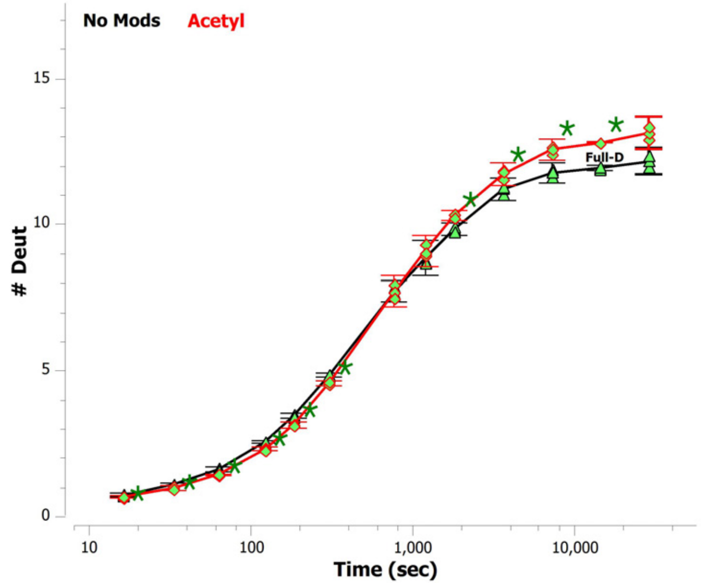

187-204: MGSKSPGNTSQPPAFFSK (#10)

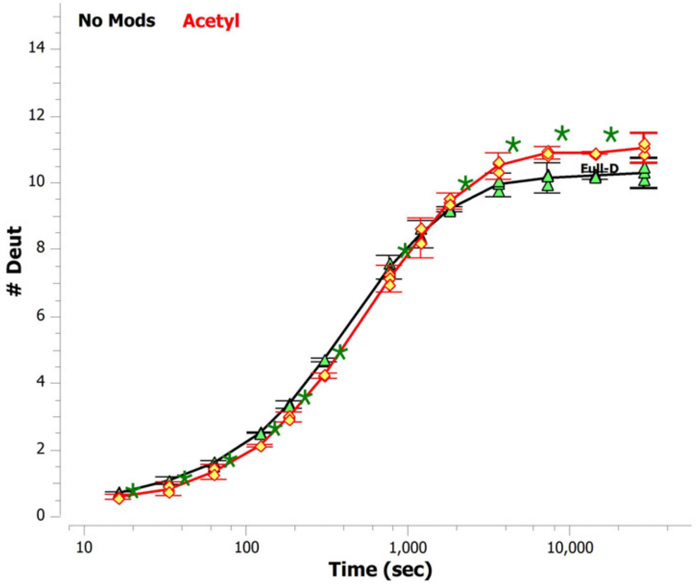

205-222: GSLGAQKLANTCFNEIEK (#11)

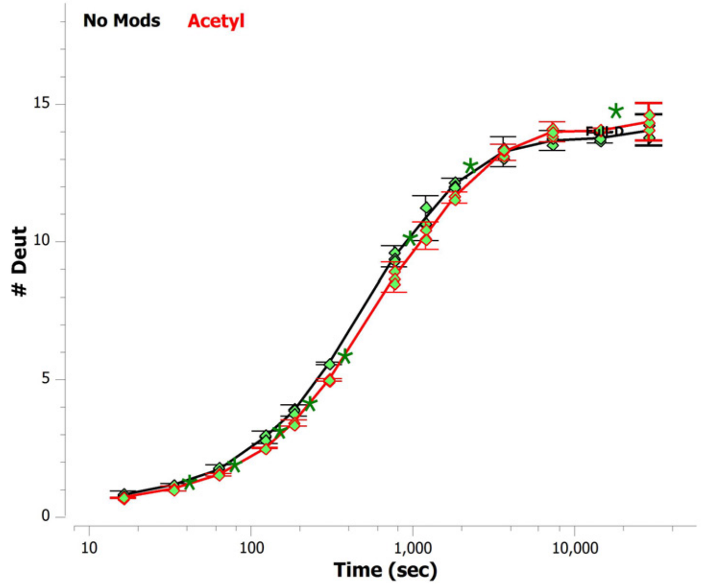

223-240: GTVPDDAVEALADSLGKK (#12)

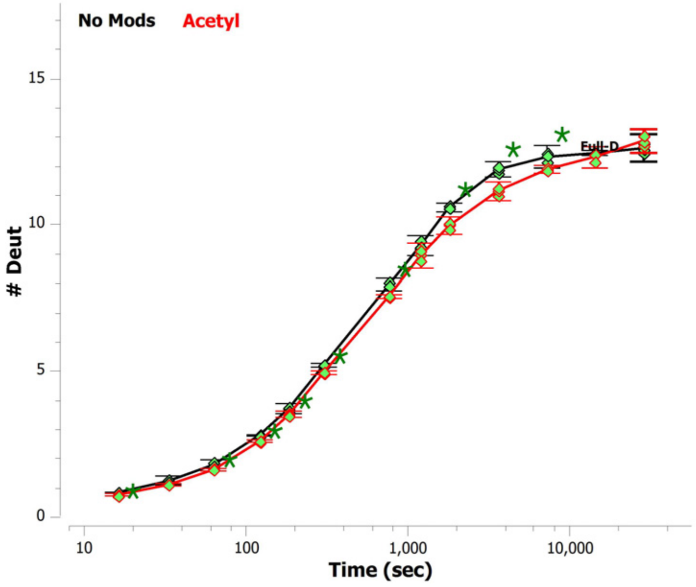

241-258: VNVEAPDVNLEGLGGKLK (#13)

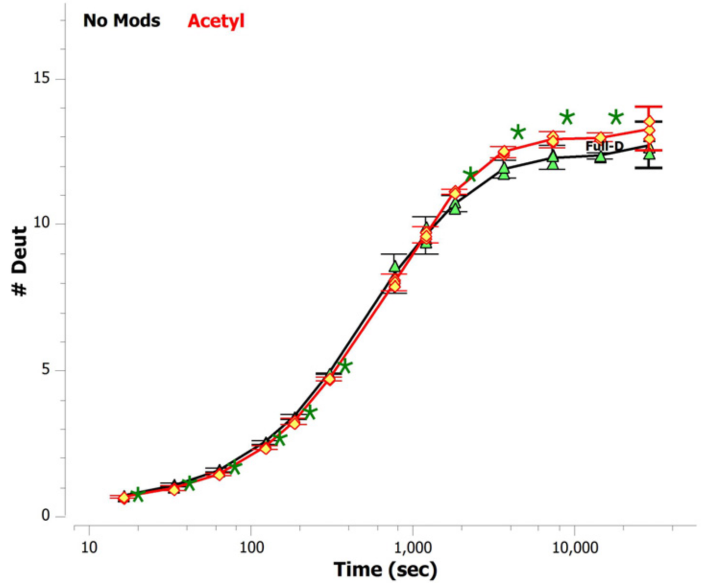

259-275: DASTLQSQKAEGTGDAK (#14)

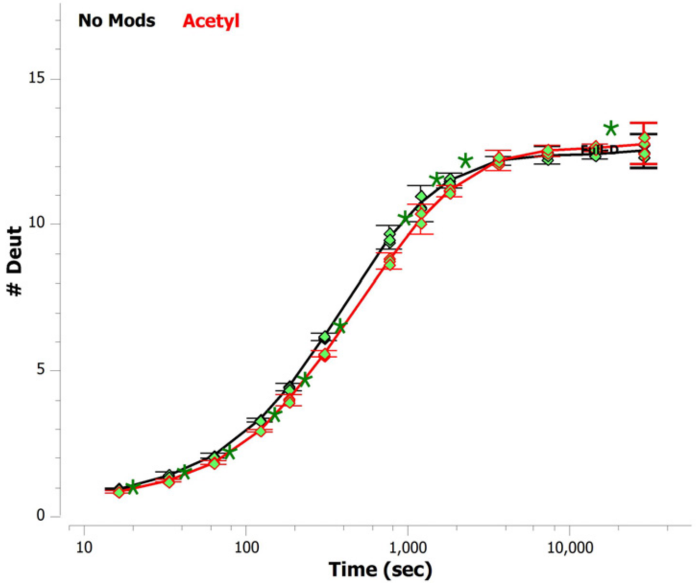

276-292: ANEKTESSSAQQVAVSR (#15)

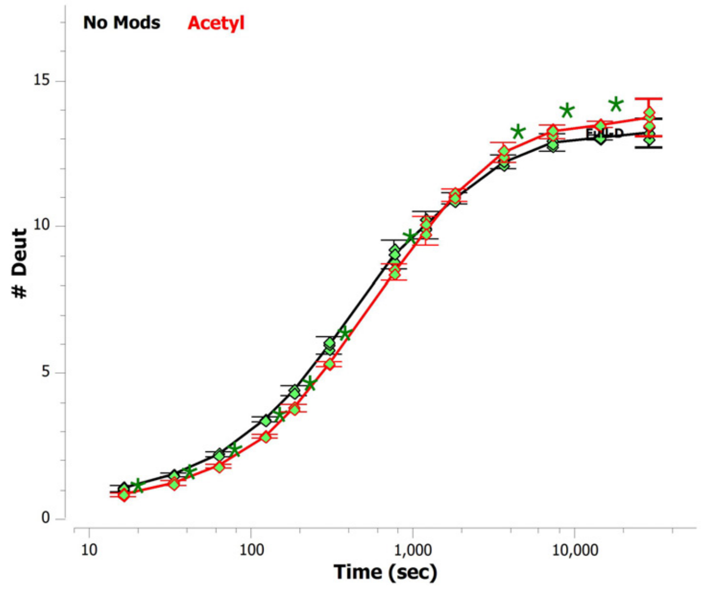

293-309: GTISAPGKVVTAAAQAK (#16)

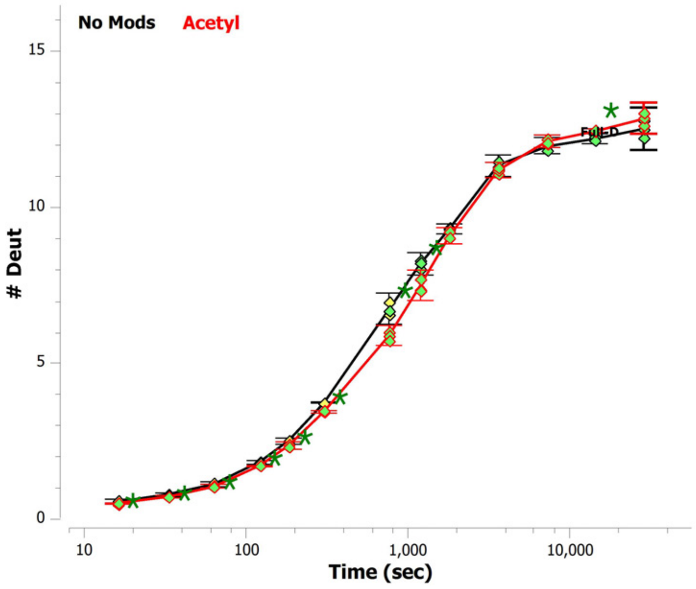

310-326: YEQEHAAIQDKLFQVAK (#17)

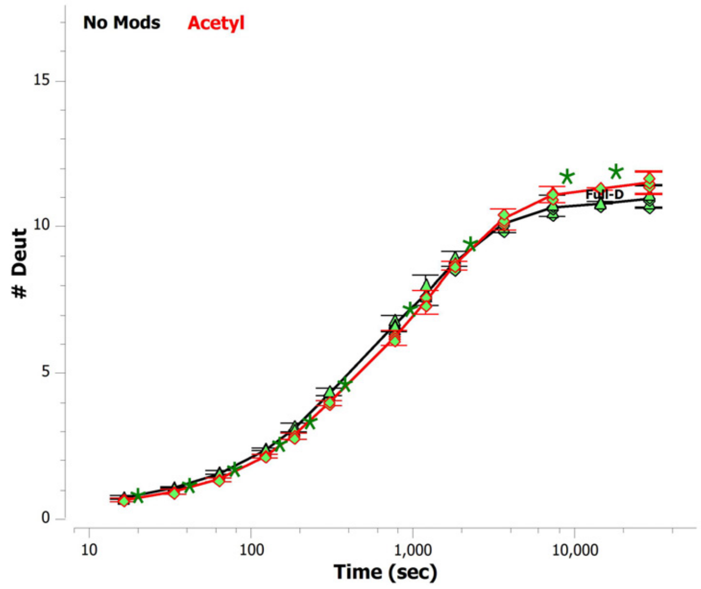

327-343: IINEPTAAAIAYGLDKK (#18)

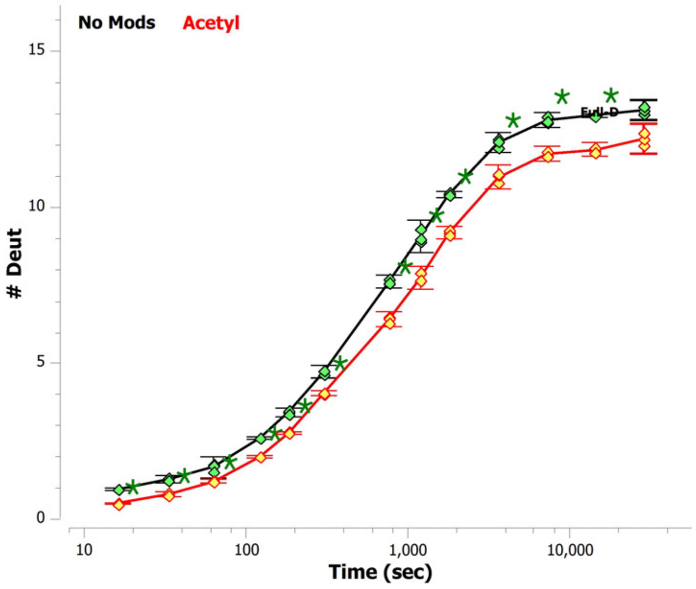

344-359: AANMLQQSGSKNTGAK (#19)

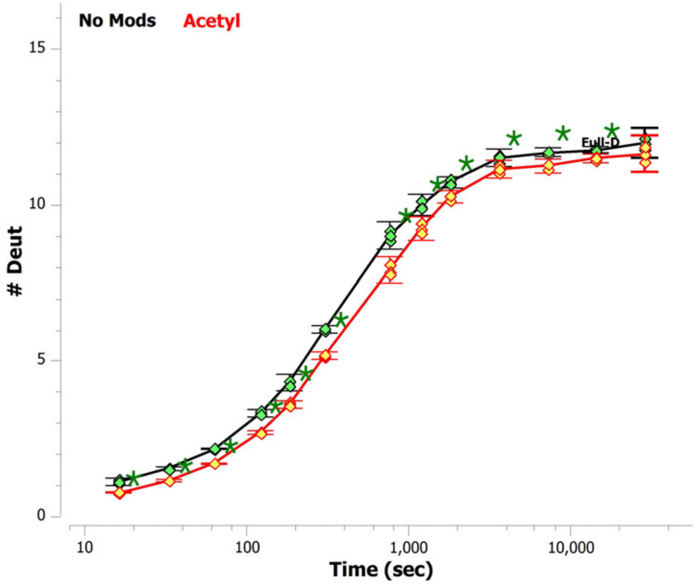

376-391: DYSSGFGGKYGVQADR (#20)

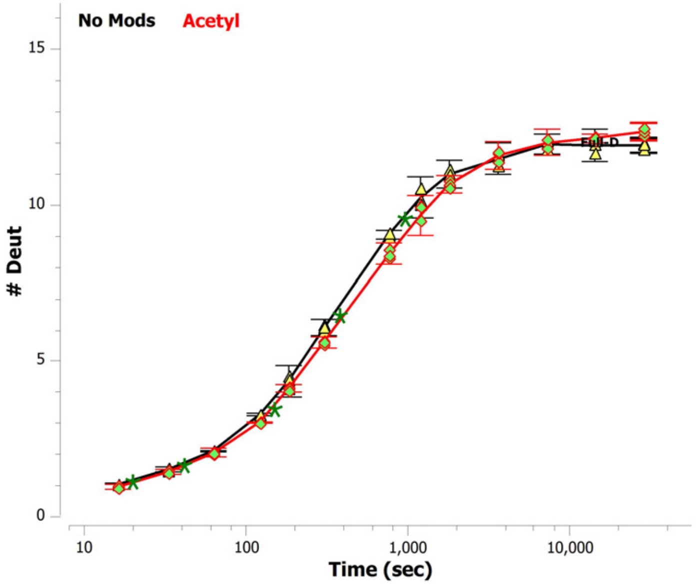

392-407: TGASWTDNIMAQKCSK (#21)

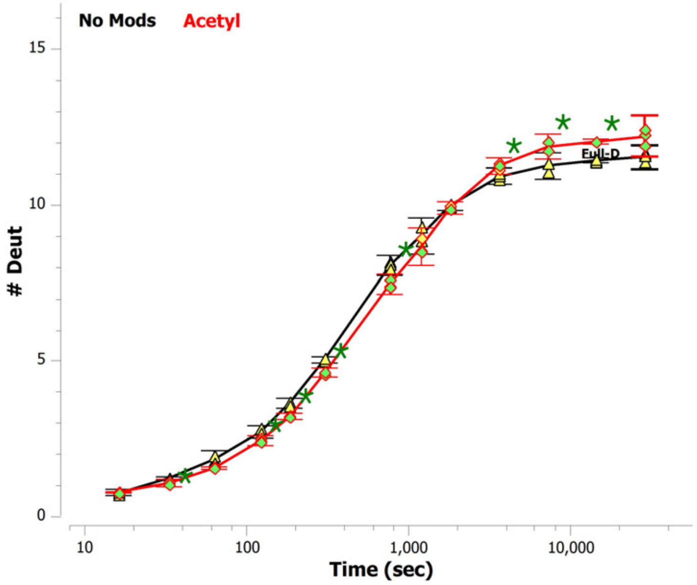

408-423: GWLKSNVSDAVAQSTR (#22)

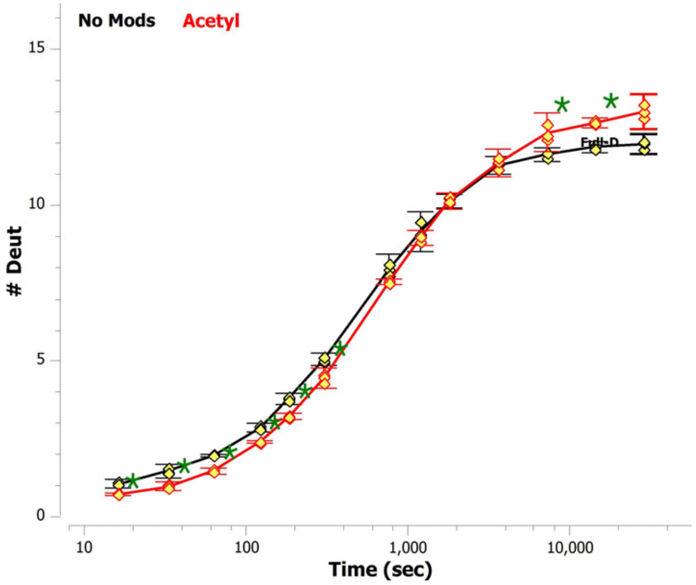

424-439: VLTANSNPSSPSAAKR (#23)

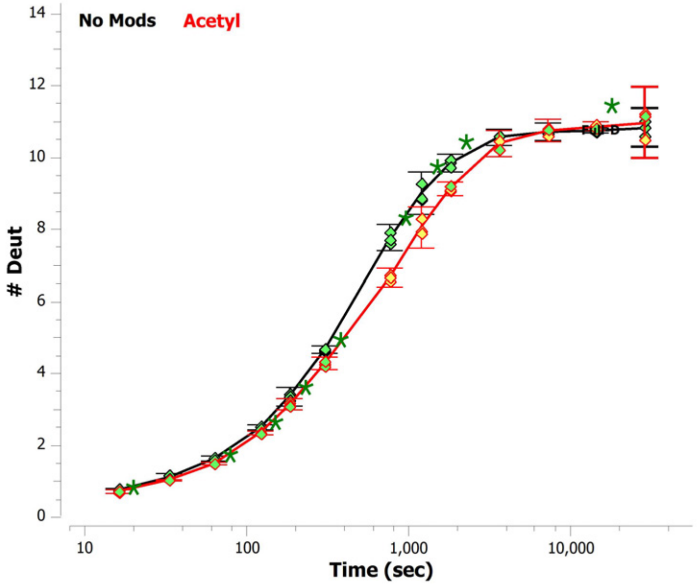

440-454: DYSAPVNFISAGLKK (#24)

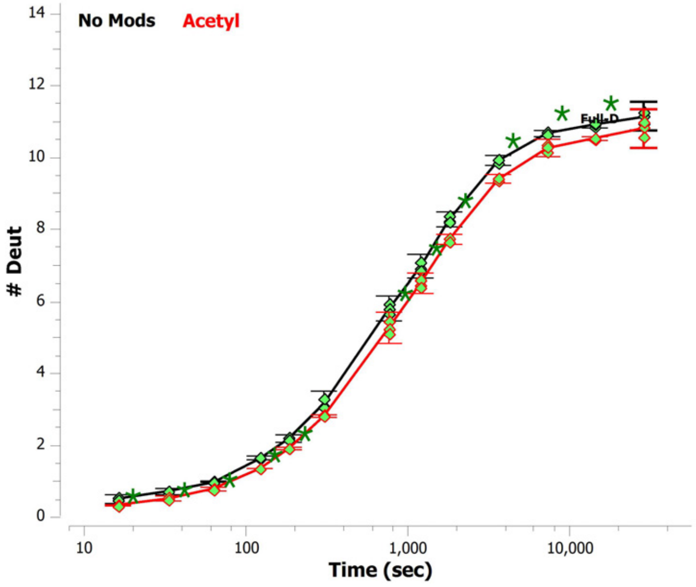

455-469: SSGPGGQNVNKVNSK (#25)

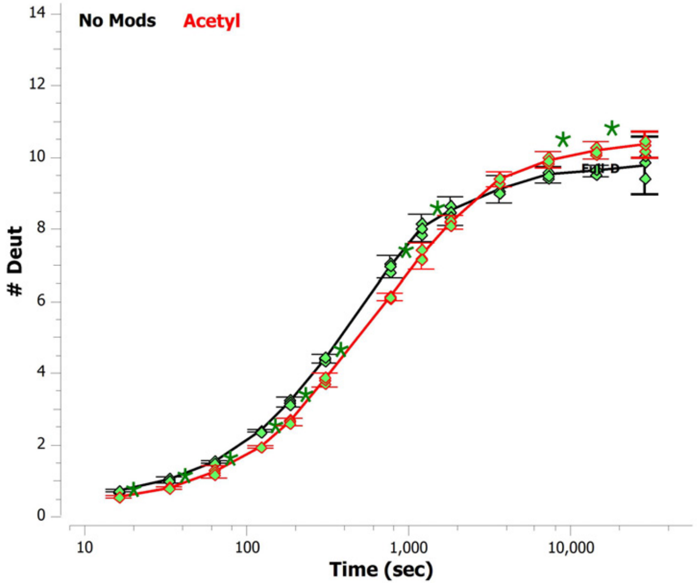

456-469: SGPGGQNVNKVNSK (#26)

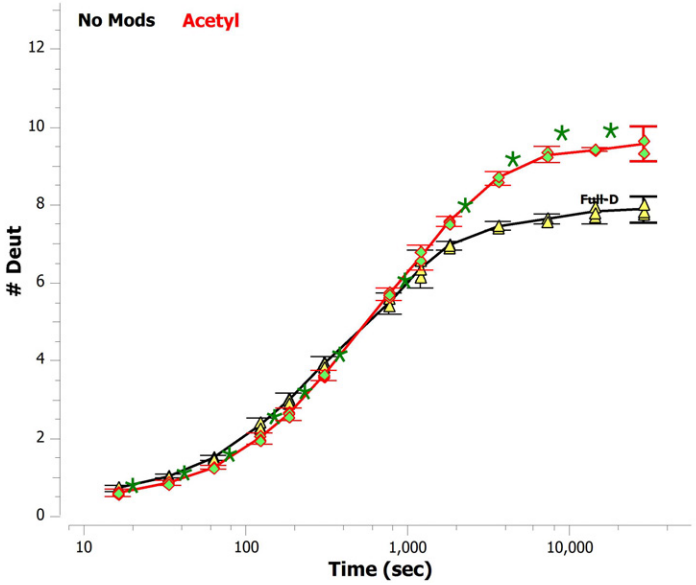

470-484: SSVACKWNLAEAQQK (#27)

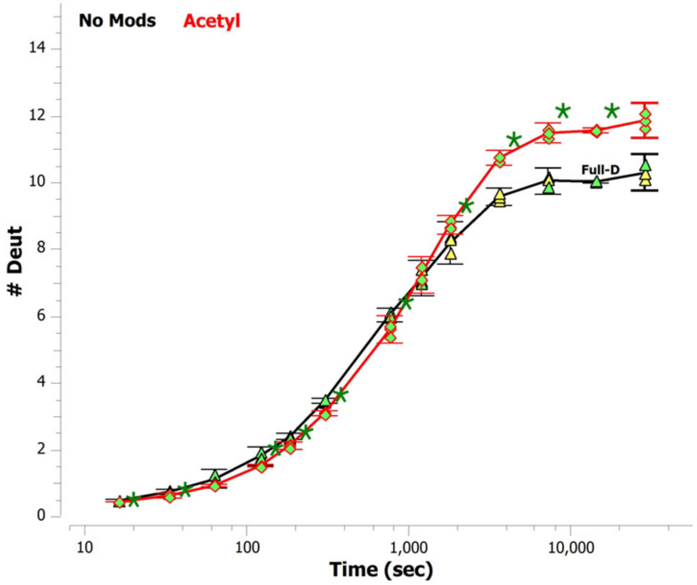

500-514: TEDEVLTSKGDAWAK (#28)

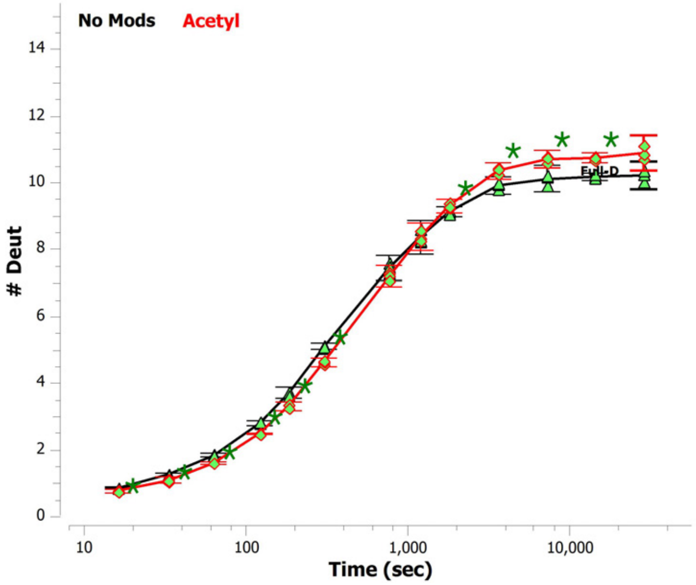

515-529: TQAYQDQKPGTSGLR (#29)

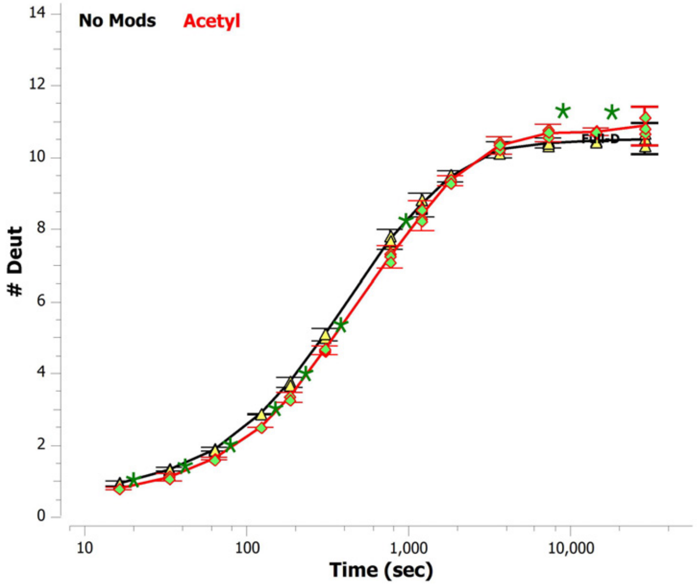

530-544: GFALVGVGSEASSKK (#30)

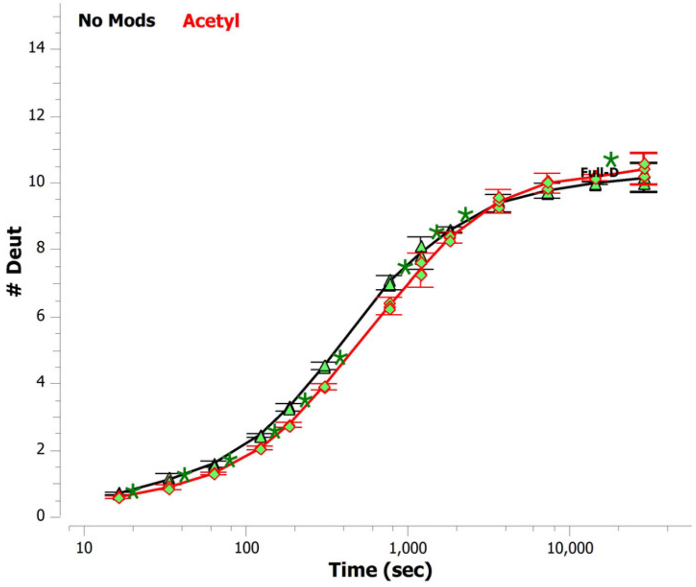

545-559: GFCFITYTDEEPVKK (#31)

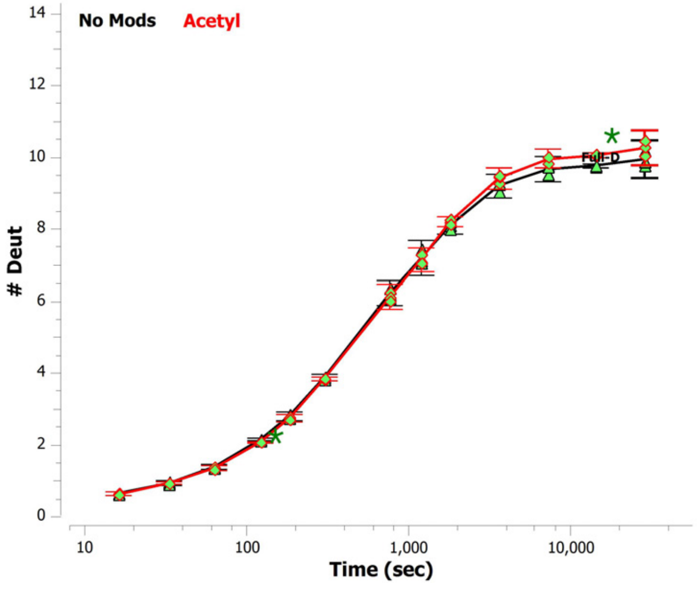

560-574: TTGFYSGFSEVAEKR (#32)

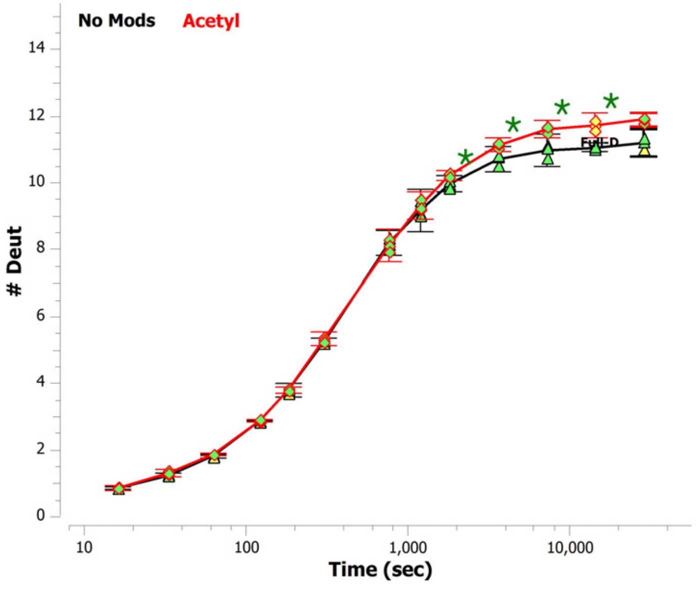

561-574: TGFYSGFSEVAEKR (#33)

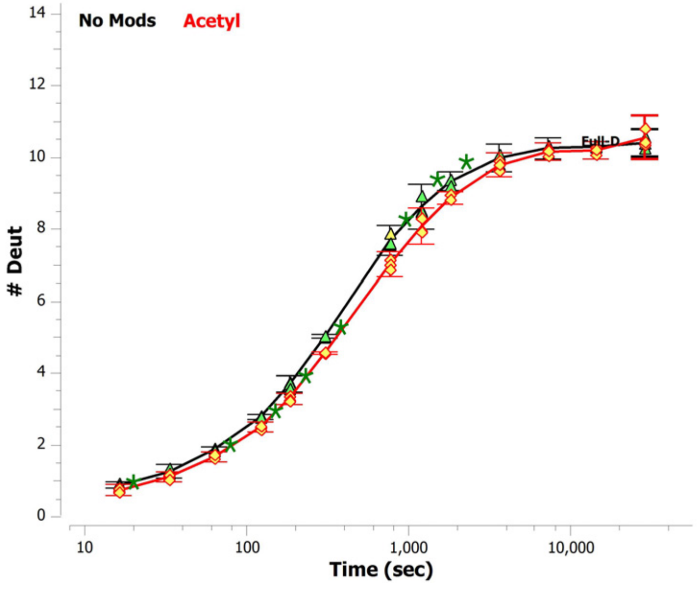

575-589: MMSKPQTSGAYVLNK (#34)

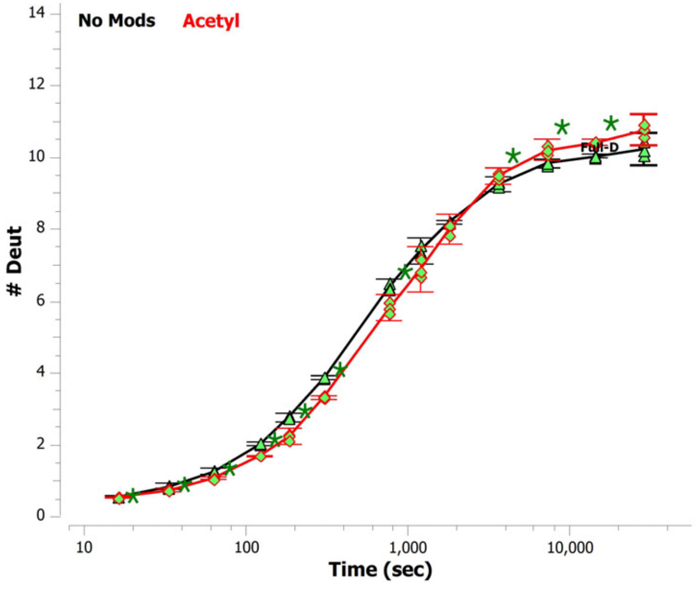

605-619: DLSTVEALQNLKNLK (#35)

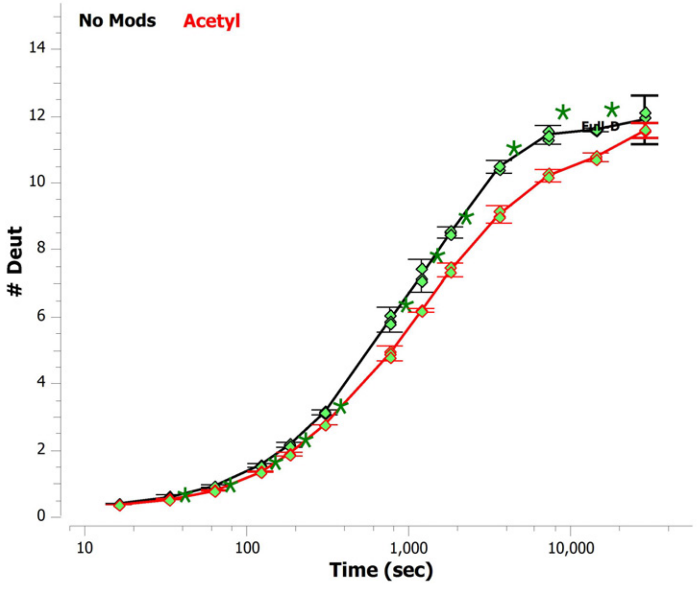

620-634: LQAAYAGDKADDIQK (#36)

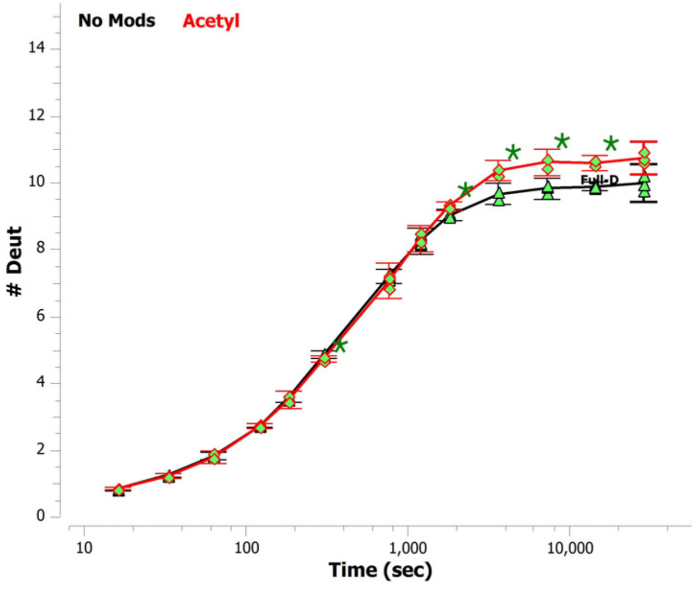

635-649: PSVGSQSNQAGQGKR (#37)

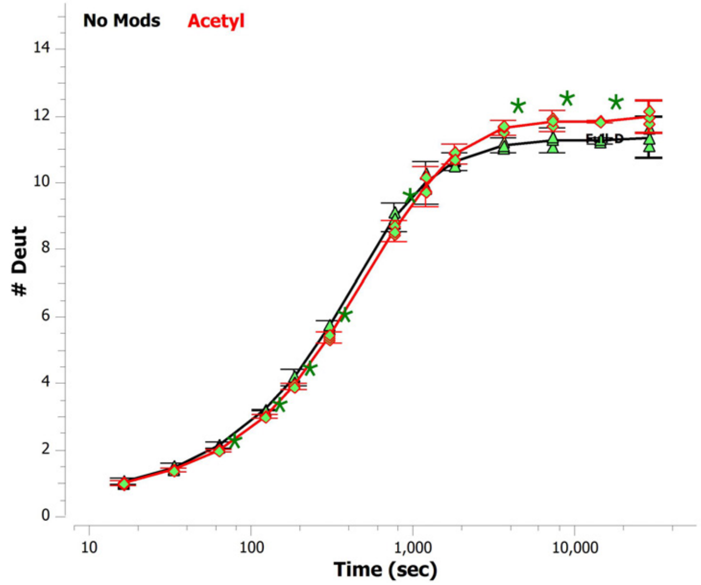

665-679: DTMGIADKTENTLER (#38)

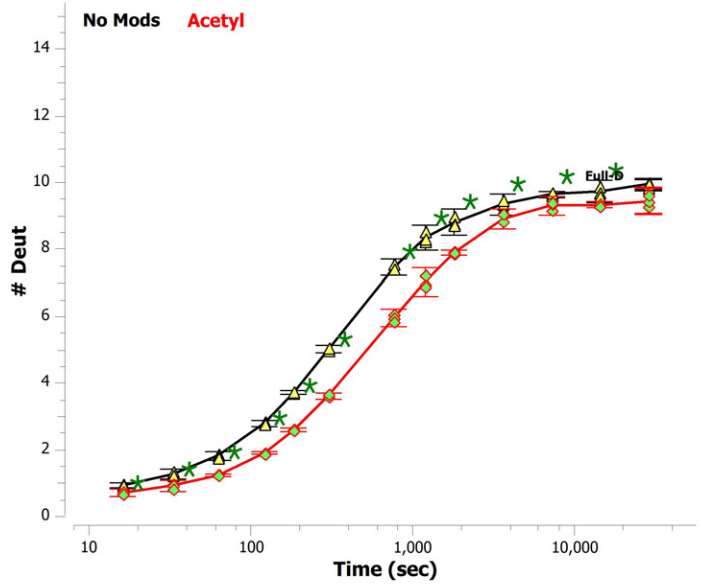

680-694: DTPTSAGPNSFNKGK (#39)

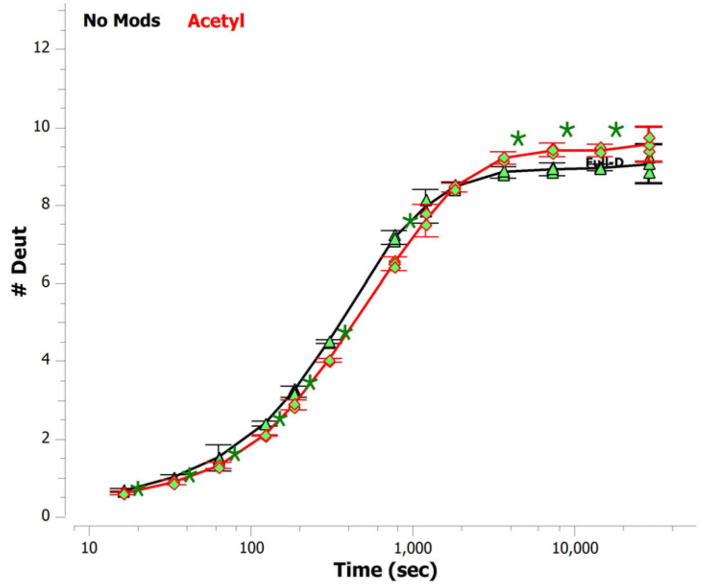

695-708: AGPNASIISLKSDK (#40)

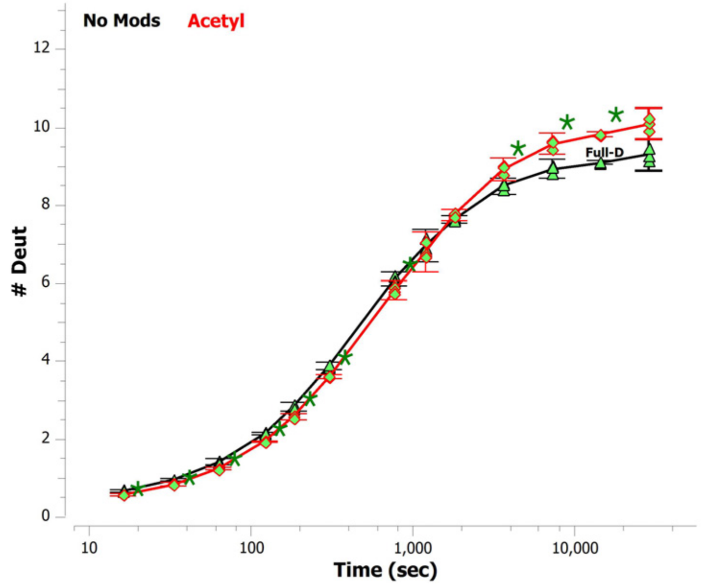

709-722: SDSGKPYYYNSQTK (#41)

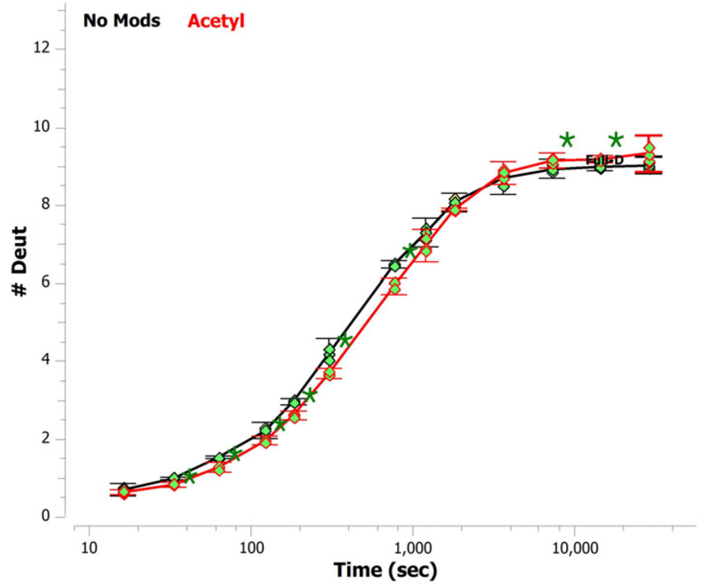

723-736: ITESVAETAQTIKK (#42)

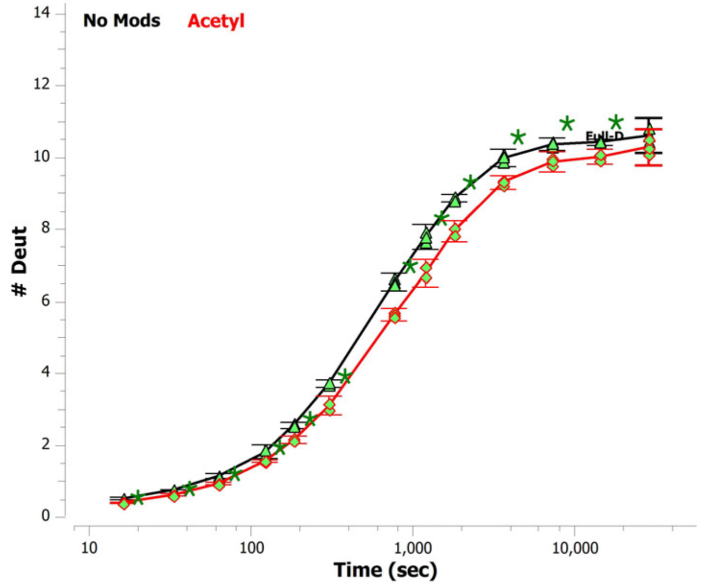

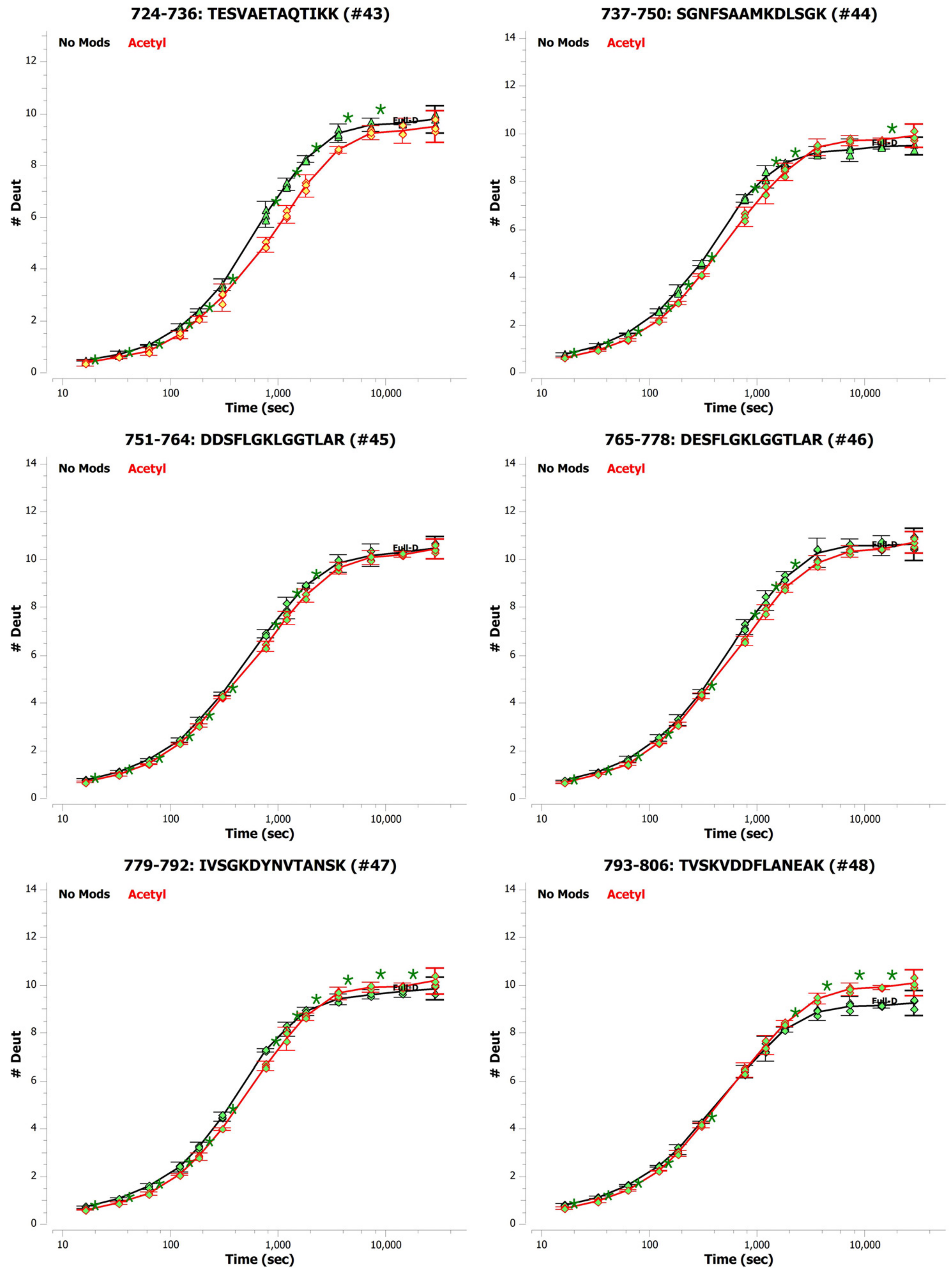
724-736: TESVAETAQTIKK (#43)
737-750: SGNFSAAMKDLSGK (#44)
751-764: DDSFLGKLGGTLAR (#45)
765-778: DESFLGKLGGTLAR (#46)
779-792: IVSGKDYNVTANSK (#47)
793-806: TVSKVDDFLANEAK (#48)
No Mods
Acetyl
Full-D
Deut
Time (sec)
0
2
4
6
8
10
12
14
10
100
1,000
10,000

807-820: FLQEFYQDDELGKK (#49)

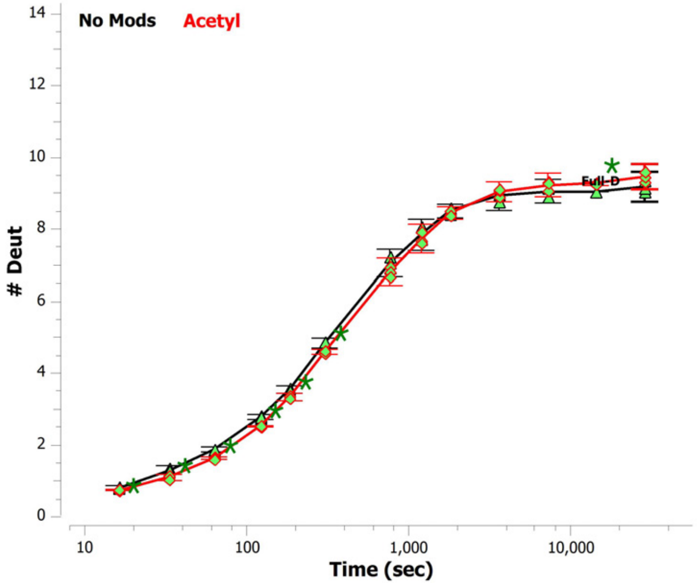

821-834: PANEKATDDYHYEK (#50)

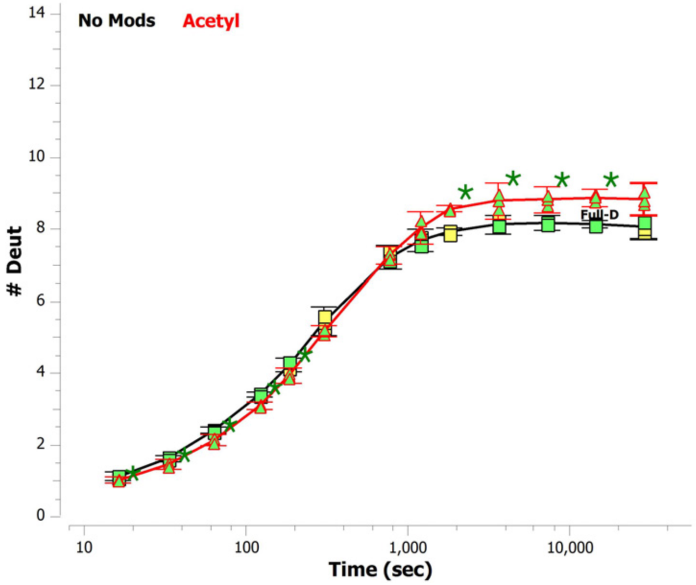

835-848: PSKGPLQSVQVFGR (#51)

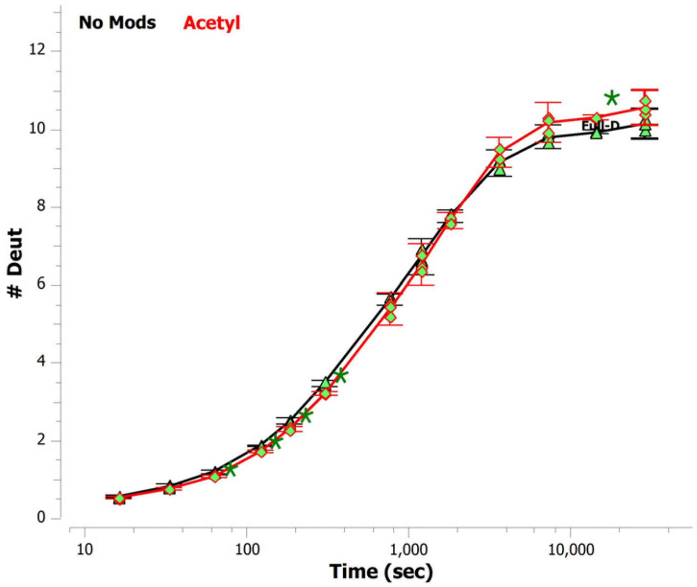

849-862: ISSNFSSIIAEKLR (#52)

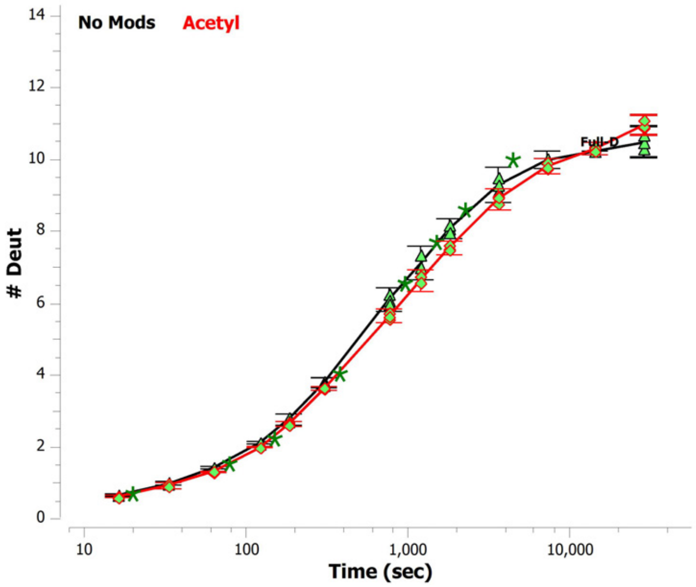

877-890: GTITVSAQELKDNR (#53)

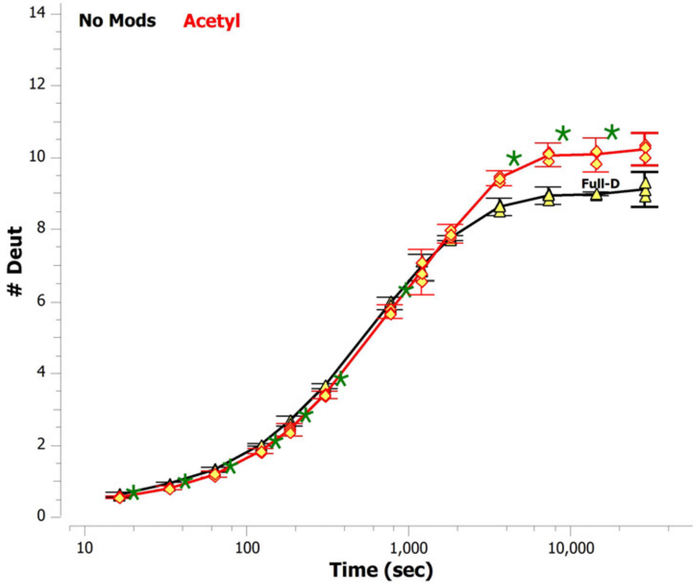

891-903: AGQKLIDVNHYAK (#54)

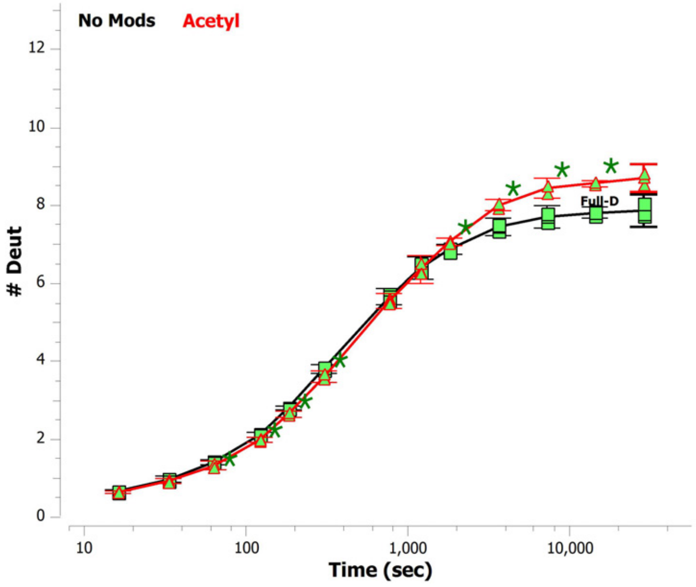

893-903: QKLIDVNHYAK (#55)

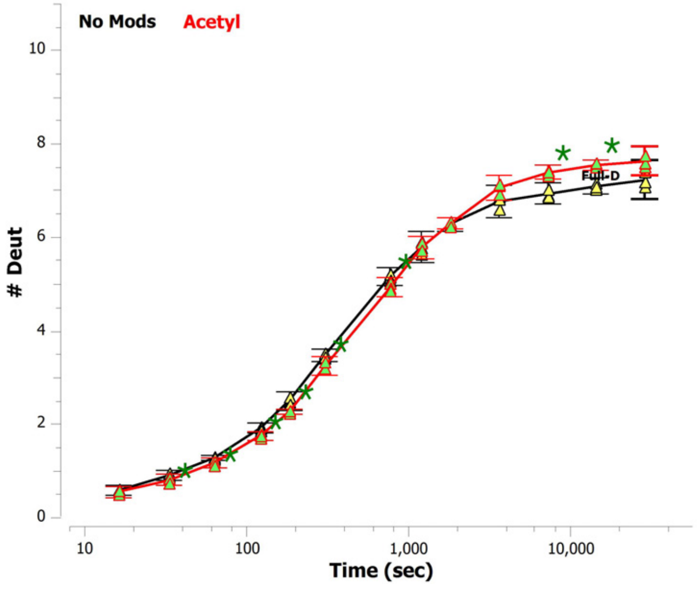

917-929: DLGKFQVATDALK (#56)

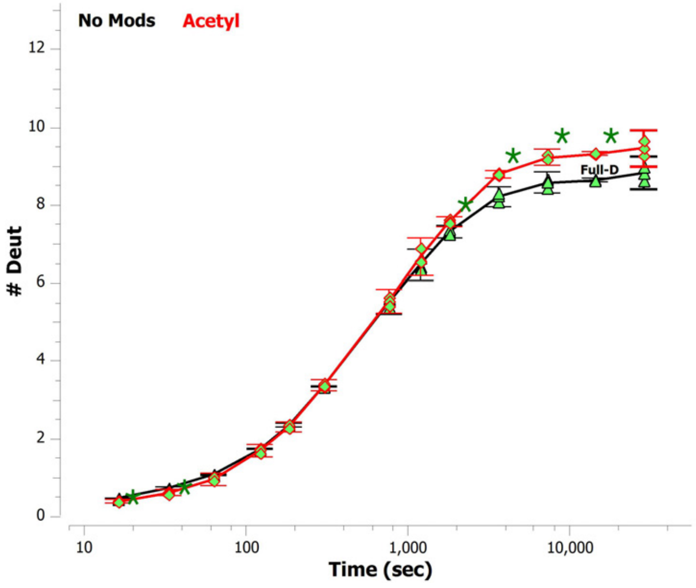

943-955: AAINQKLIETGER (#57)

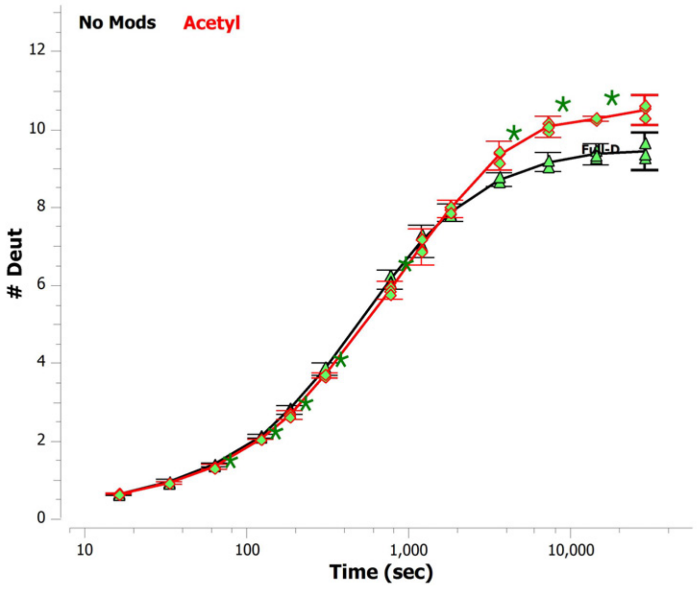

956-968: TEVLSPNSKVESK (#58)

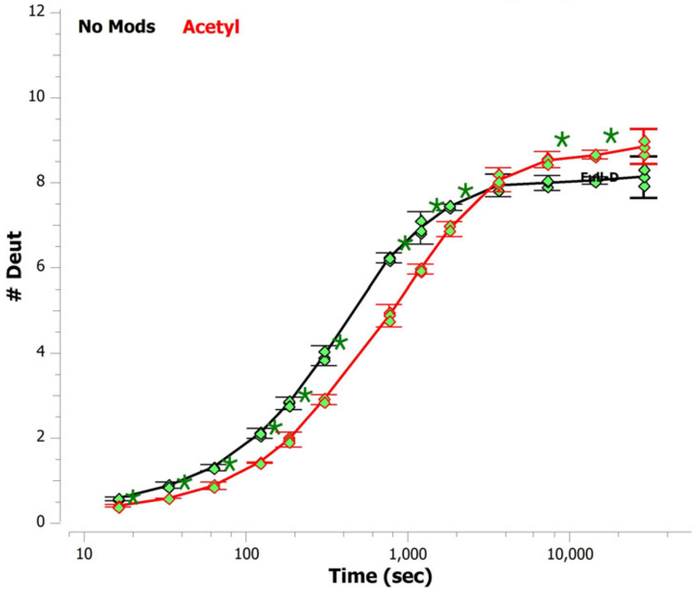

969-981: GETASKLQSEISR (#59)

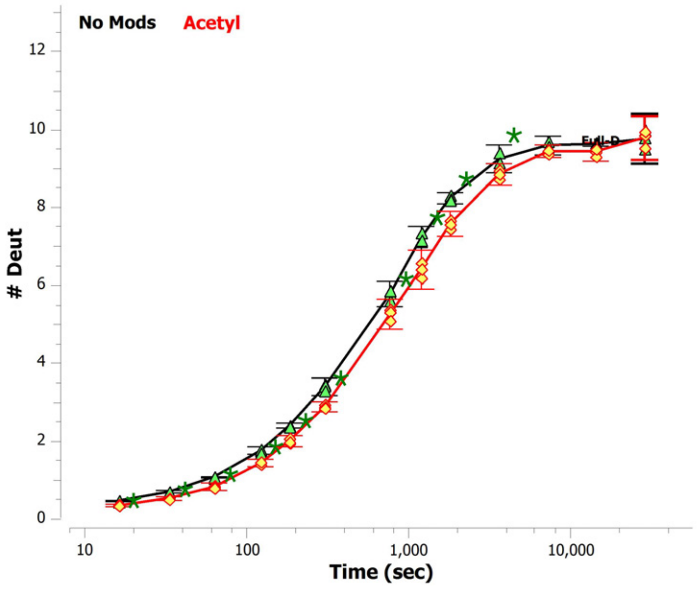

995-1007: LQEKVESAQSEQK (#60)

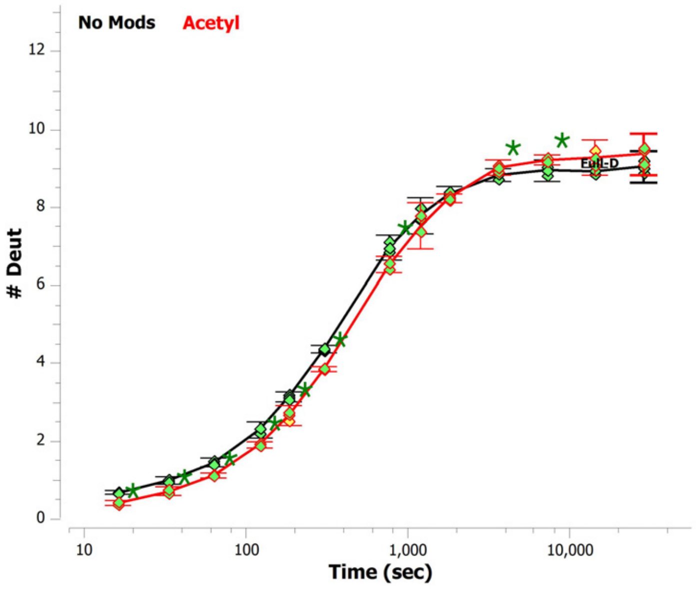

1008-1020: SQNTDMVQKSVSK (#61)

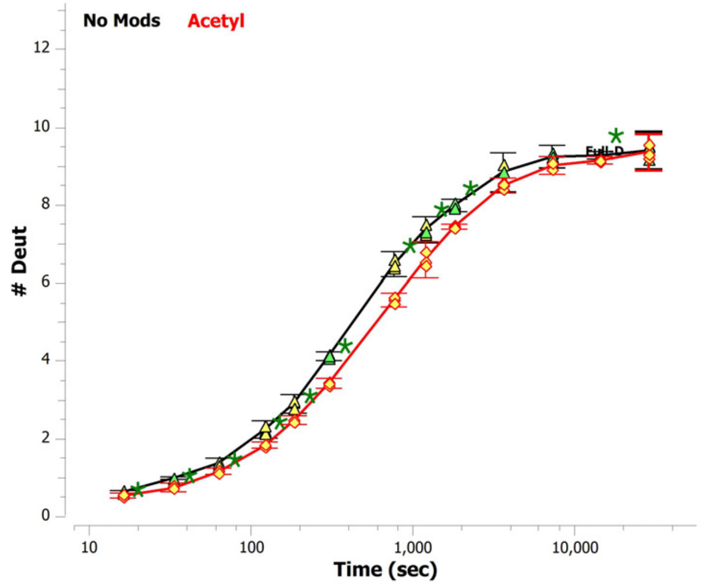

1021-1033: LQDVSGQLSSSKK (#62)

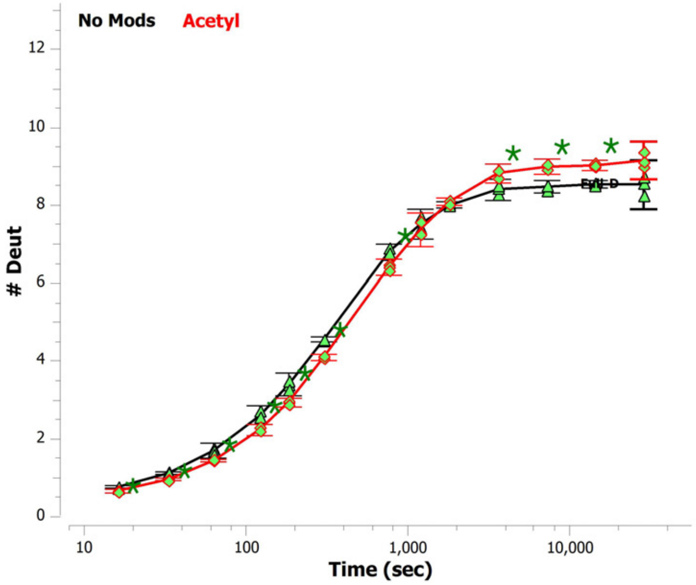

1047-1059: TPGQNAQKWIPAR (#63)

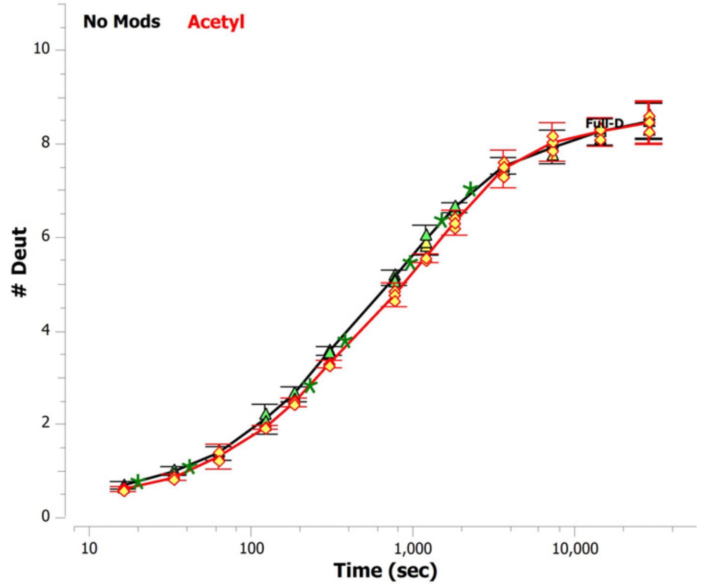

1060-1072: YEEIVKEVSTYIK (#64)

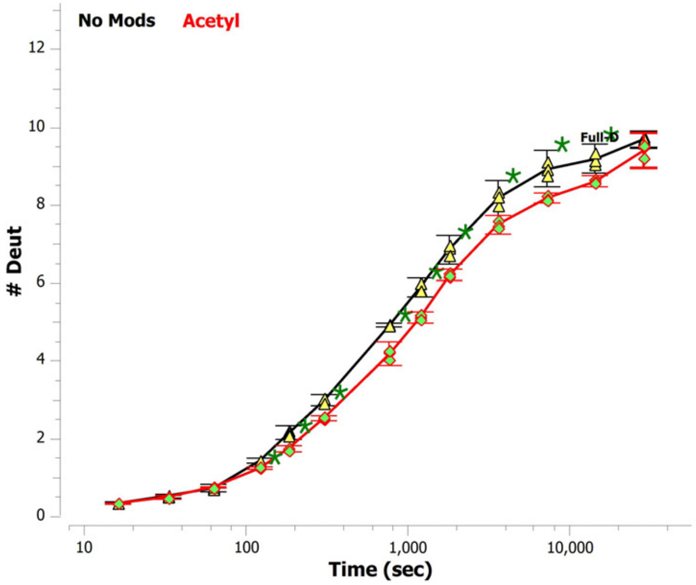

1073-1084: LTEKELAEAASK (#65)

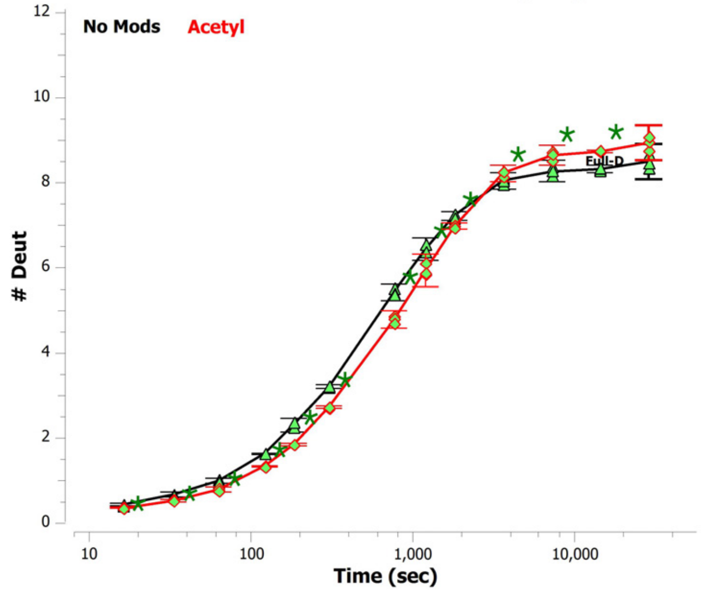

1085-1096: LLFSNTAAQKLR (#66)

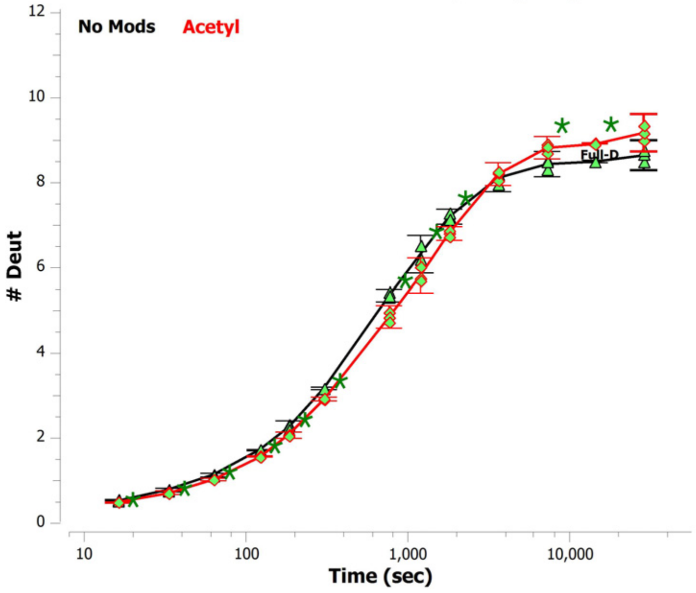

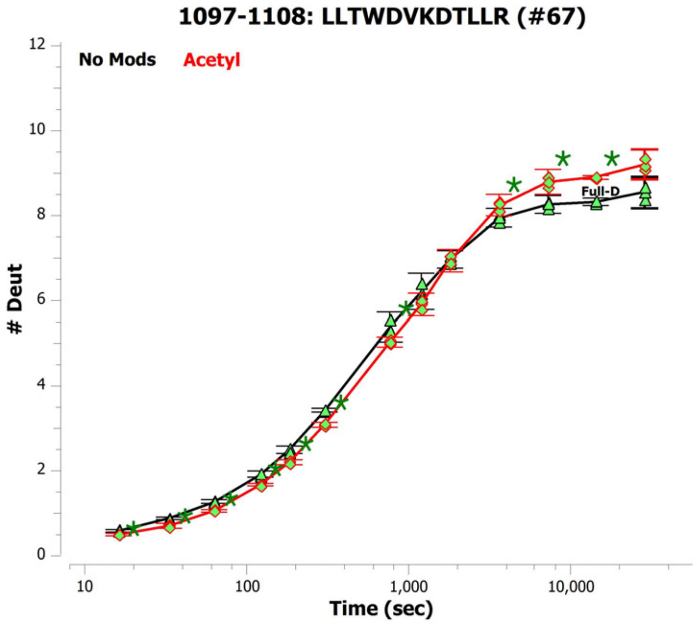
1097-1108: LLTWDVKDTLLR (#67)
No Mods
Acetyl
Full-D
Deut
Time (sec)
0
2
4
6
8
10
12
10
100
1,000
10,000

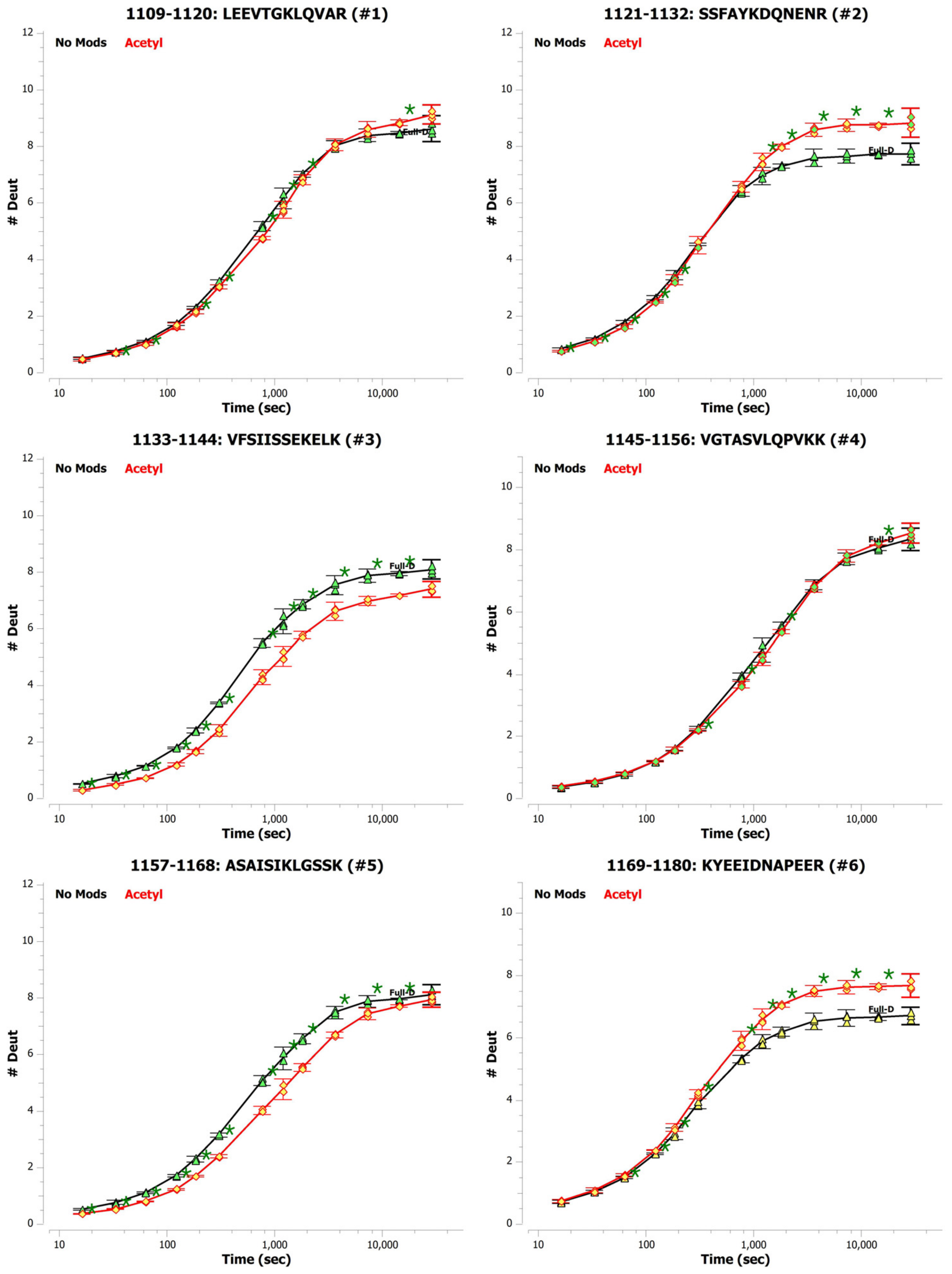
1109-1120: LEEVTGKLQVAR (#1)
1121-1132: SSFAYKDQNENR (#2)
1133-1144: VFSIISSEKELK (#3)
1145-1156: VGTASVLQPVKK (#4)
1157-1168: ASAISIKLGSSK (#5)
1169-1180: KYEEIDNAPEER (#6)
No Mods
Acetyl
Full-D
Deut
Time (sec)
0
2
4
6
8
10
12
10
100
1,000
10,000

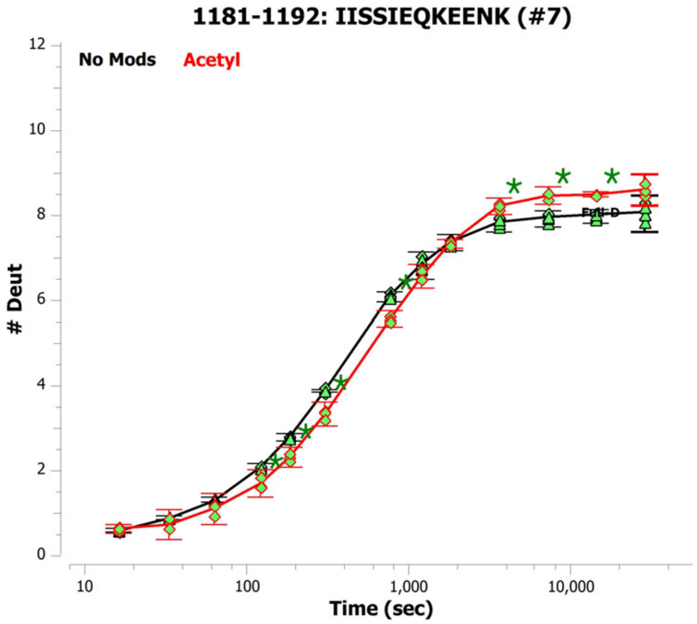
1181-1192: IISSIEQKEENK (#7)
No Mods
Acetyl
Full-D
Deut
Time (sec)

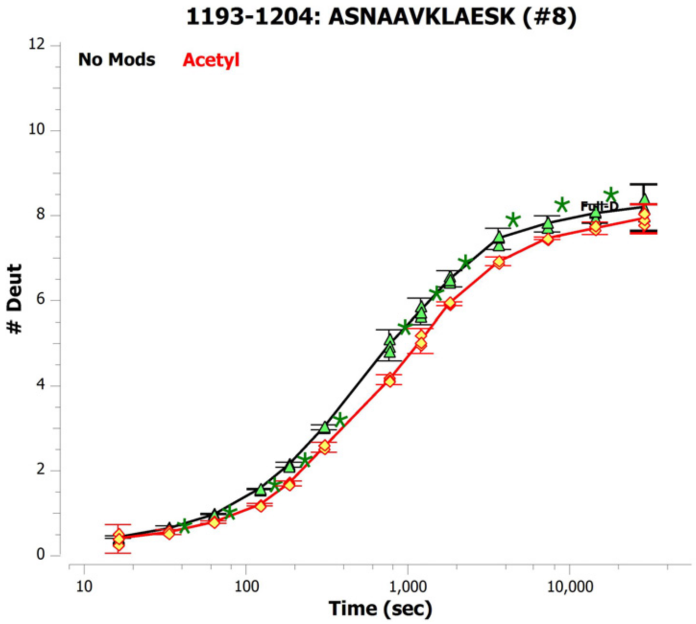
1193-1204: ASNAAVKLAESK (#8)
No Mods
Acetyl
Full-D
Deut
Time (sec)

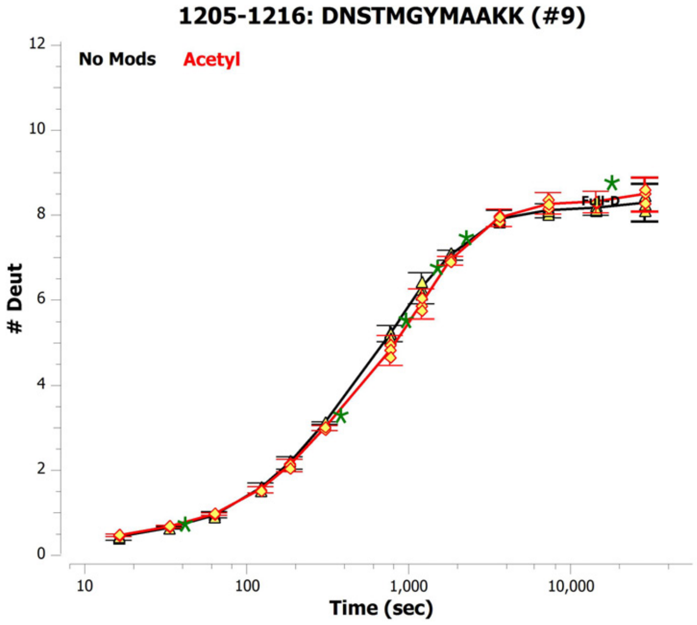
1205-1216: DNSTMGYMAAKK (#9)
No Mods
Acetyl
Full-D
Deut
Time (sec)

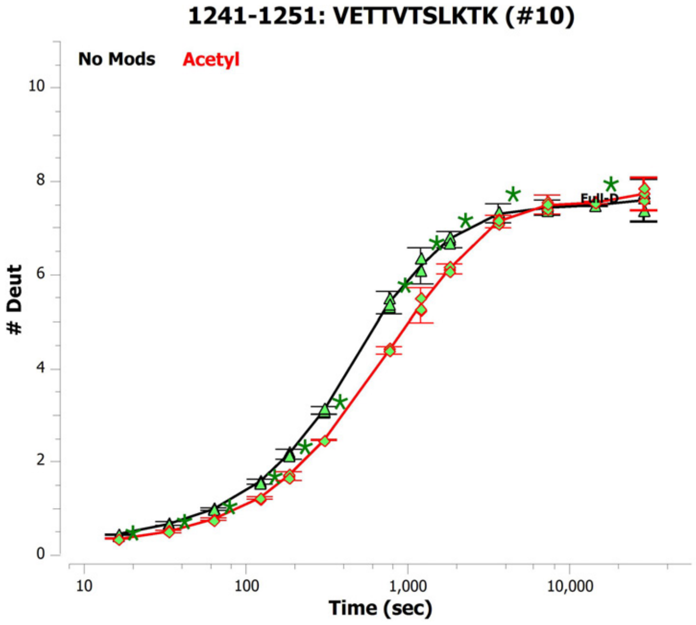
1241-1251: VETTVTSLKTK (#10)
No Mods
Acetyl
Full-D
Deut
Time (sec)

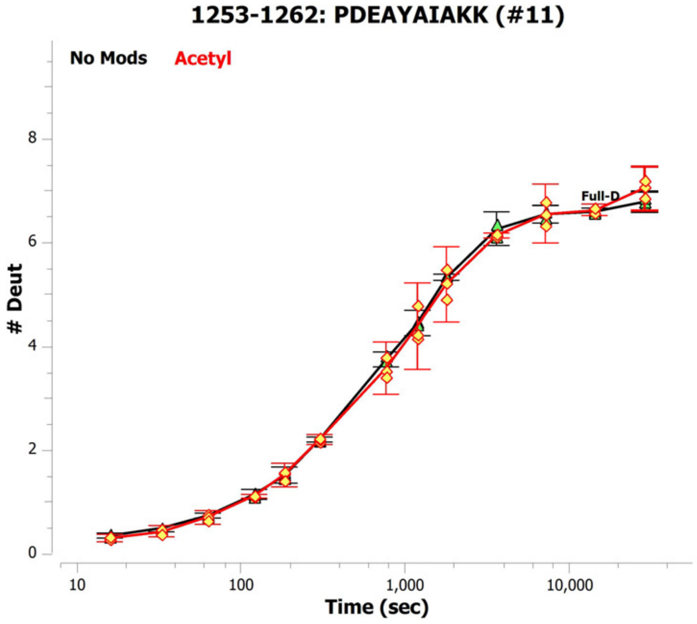
1253-1262: PDEAYAIAKK (#11)
No Mods
Acetyl
Full-D
Deut
Time (sec)

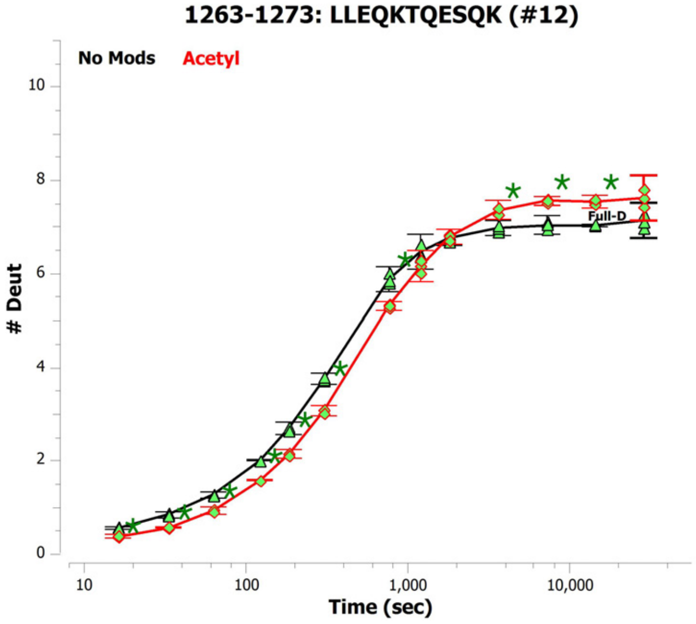
1263-1273: LLEQKTQESQK (#12)
No Mods
Acetyl
Full-D
Deut
Time (sec)

1274-1284: LEKTIDDLEEK (#13)

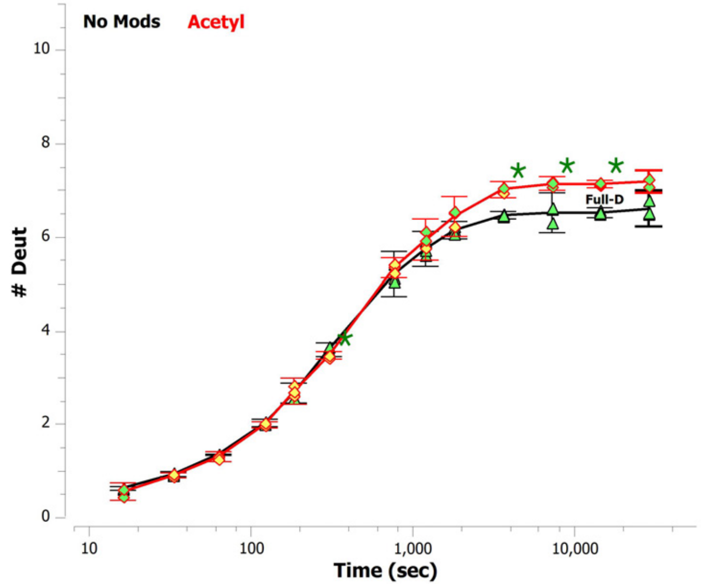

1285-1295: DFDTALKHYDK (#14)

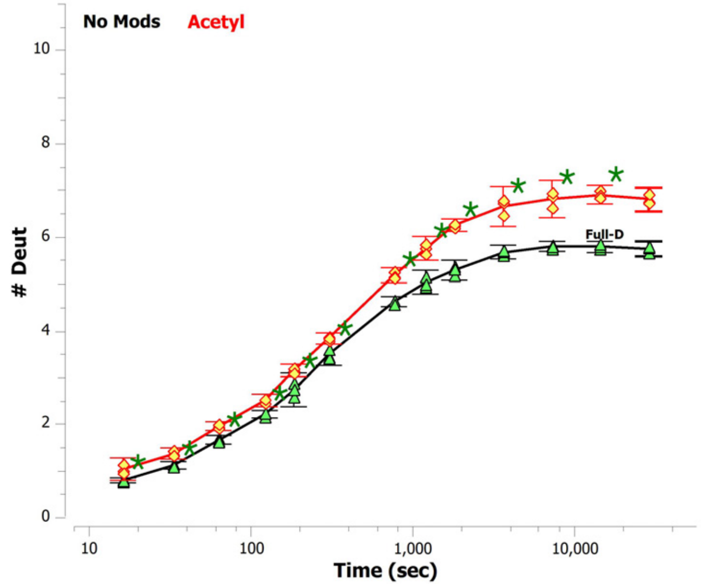

1296-1306: ITGKNQVTATK (#15)

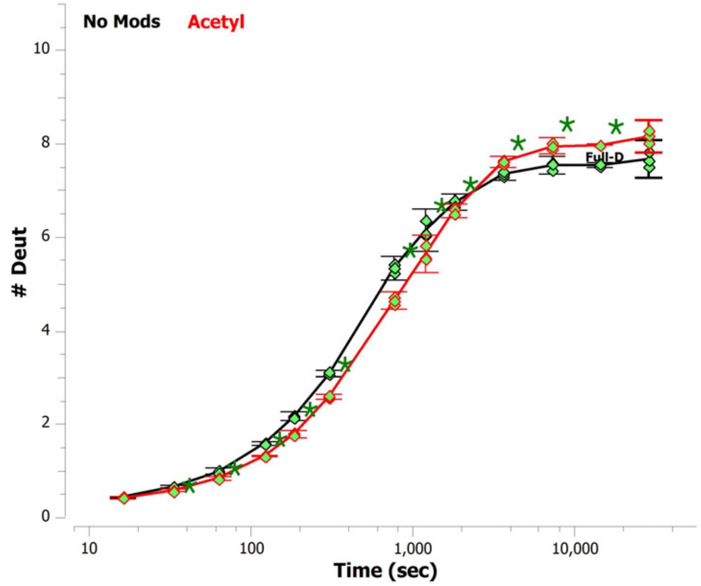

1297-1306: TGKNQVTATK (#16)

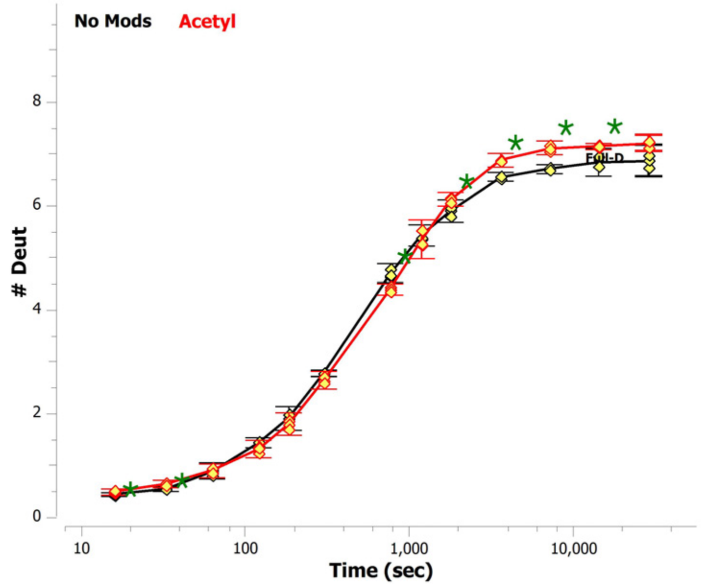

1307-1316: ASEALLKQLK (#17)

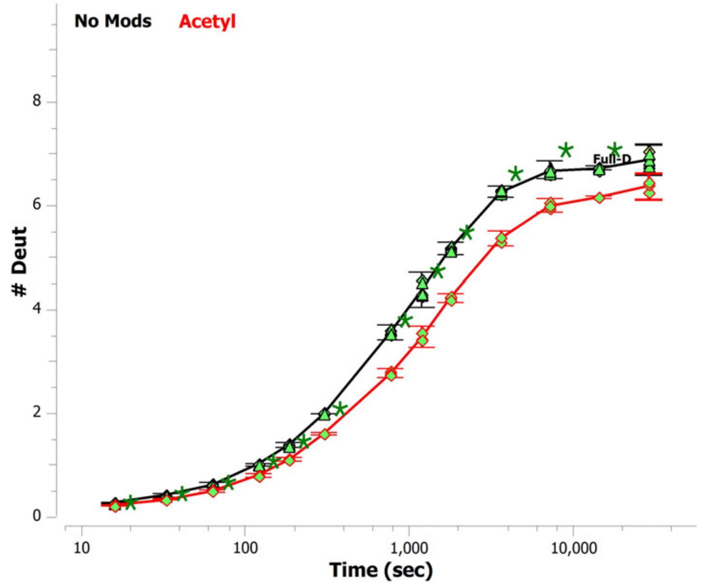

1317-1326: LLEEEDSKLK (#18)

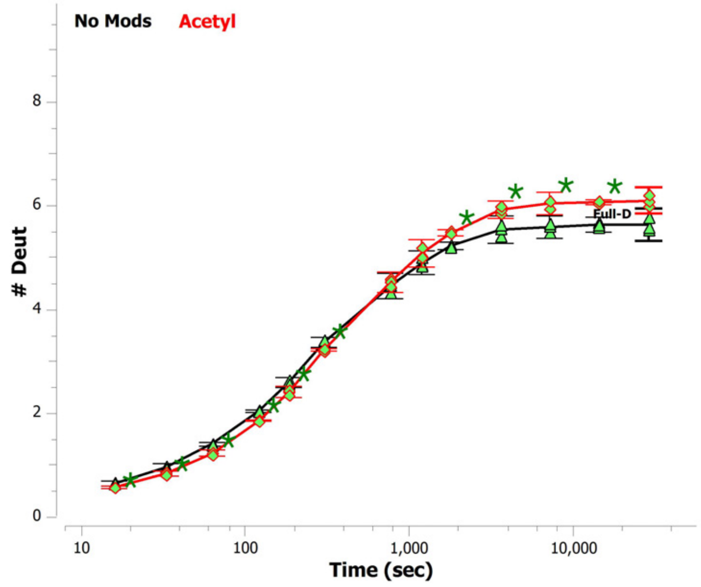

1327-1336: ISALQGKLSK (#19)

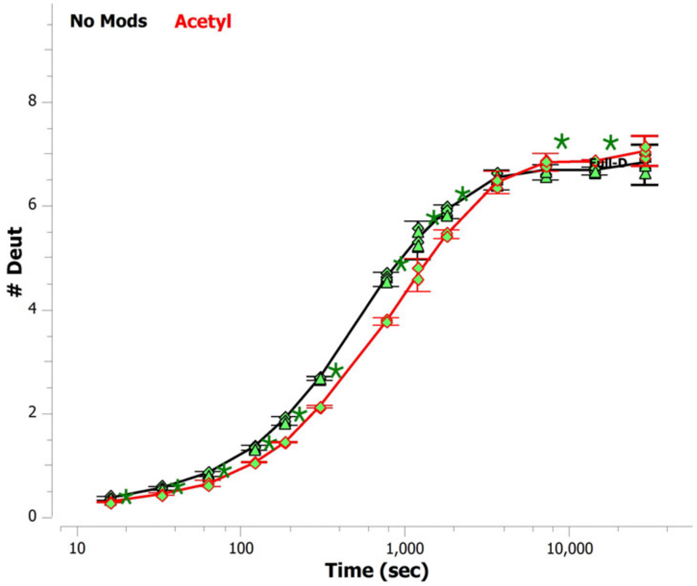

1337-1346: VNALKNLQVK (#20)

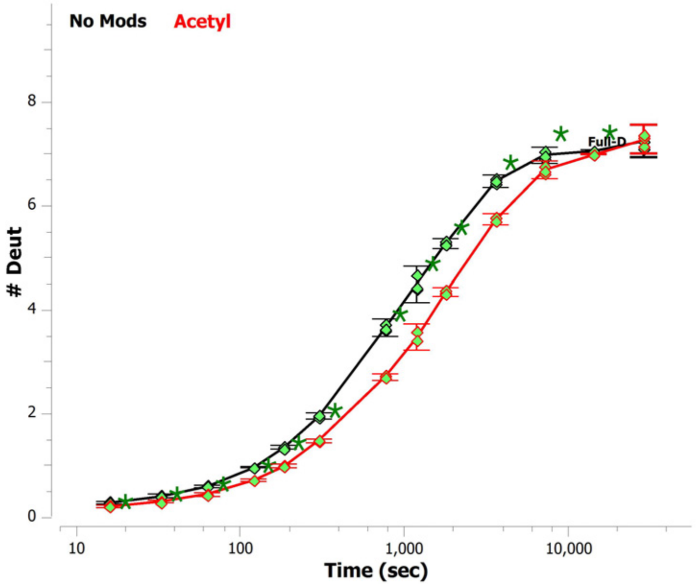

1347-1356: TPNLYIYSKK (#21)

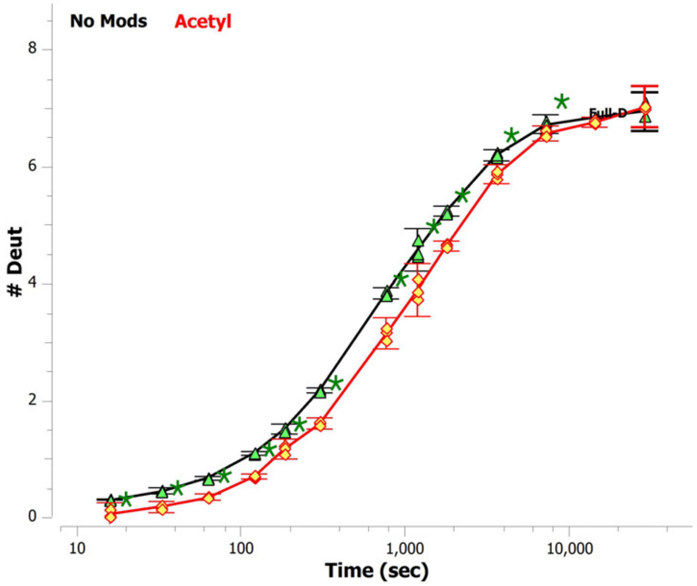

1357-1366: DQEALMKSVK (#22)

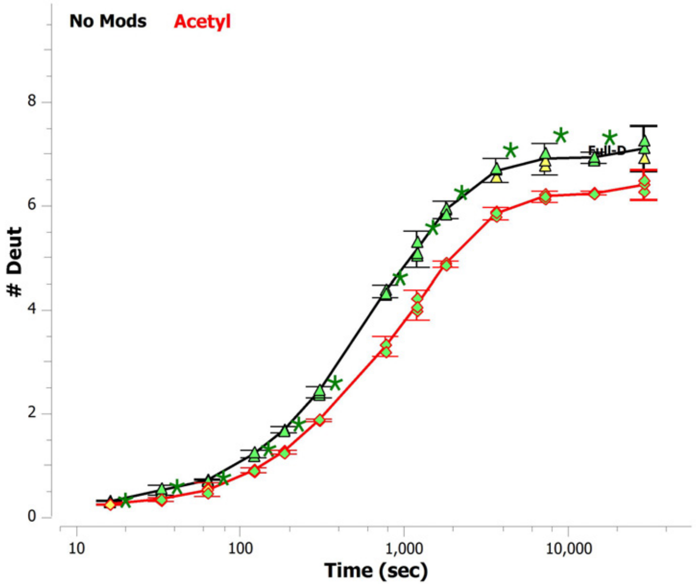

1367-1376: KFGVLSDNFK (#23)

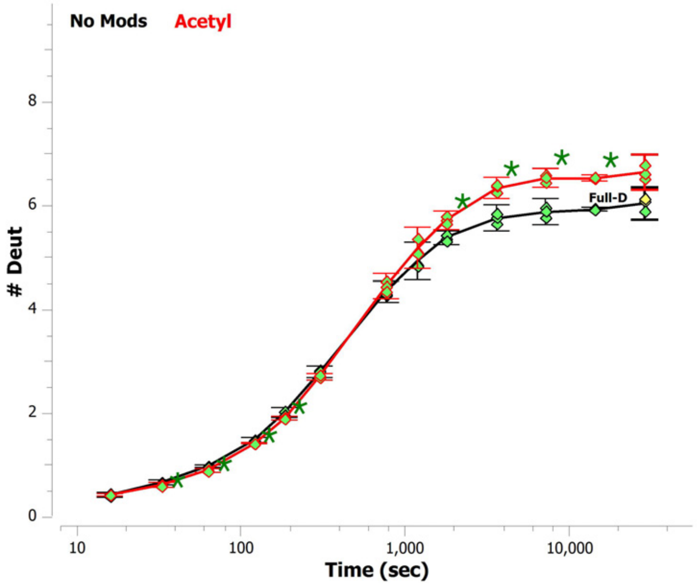

1377-1386: IQQELQTAKK (#24)

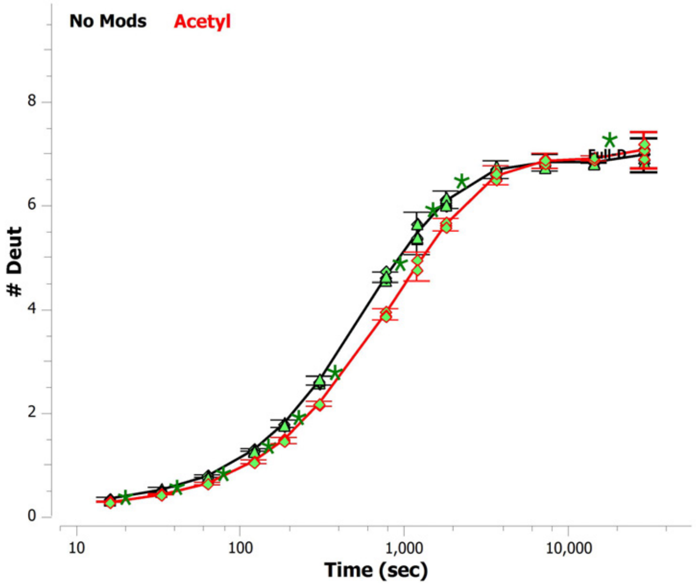

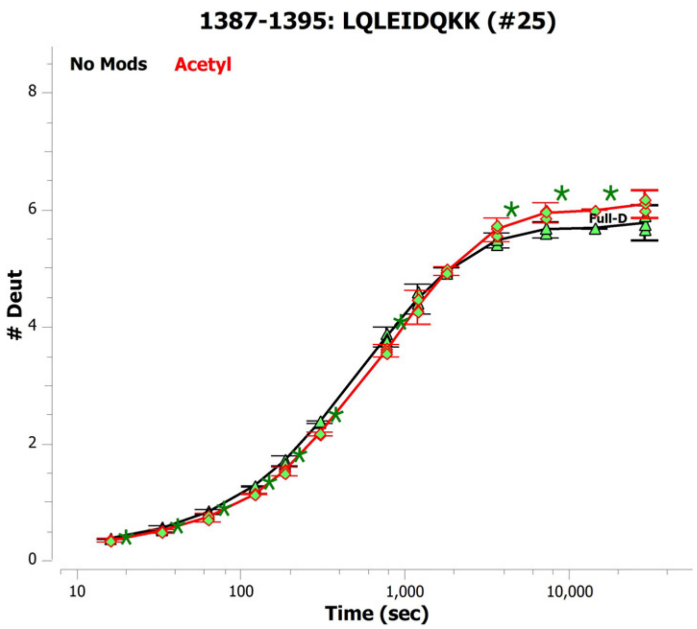

1387-1395: LQLEIDQKK (#25)
No Mods
Acetyl
Deut
Time (sec)
Full-D
0
2
4
6
8
10
100
1,000
10,000

Supplemental Figure 5B: D-uptake plots of non-modified and acetylated peptides (FD normalized)

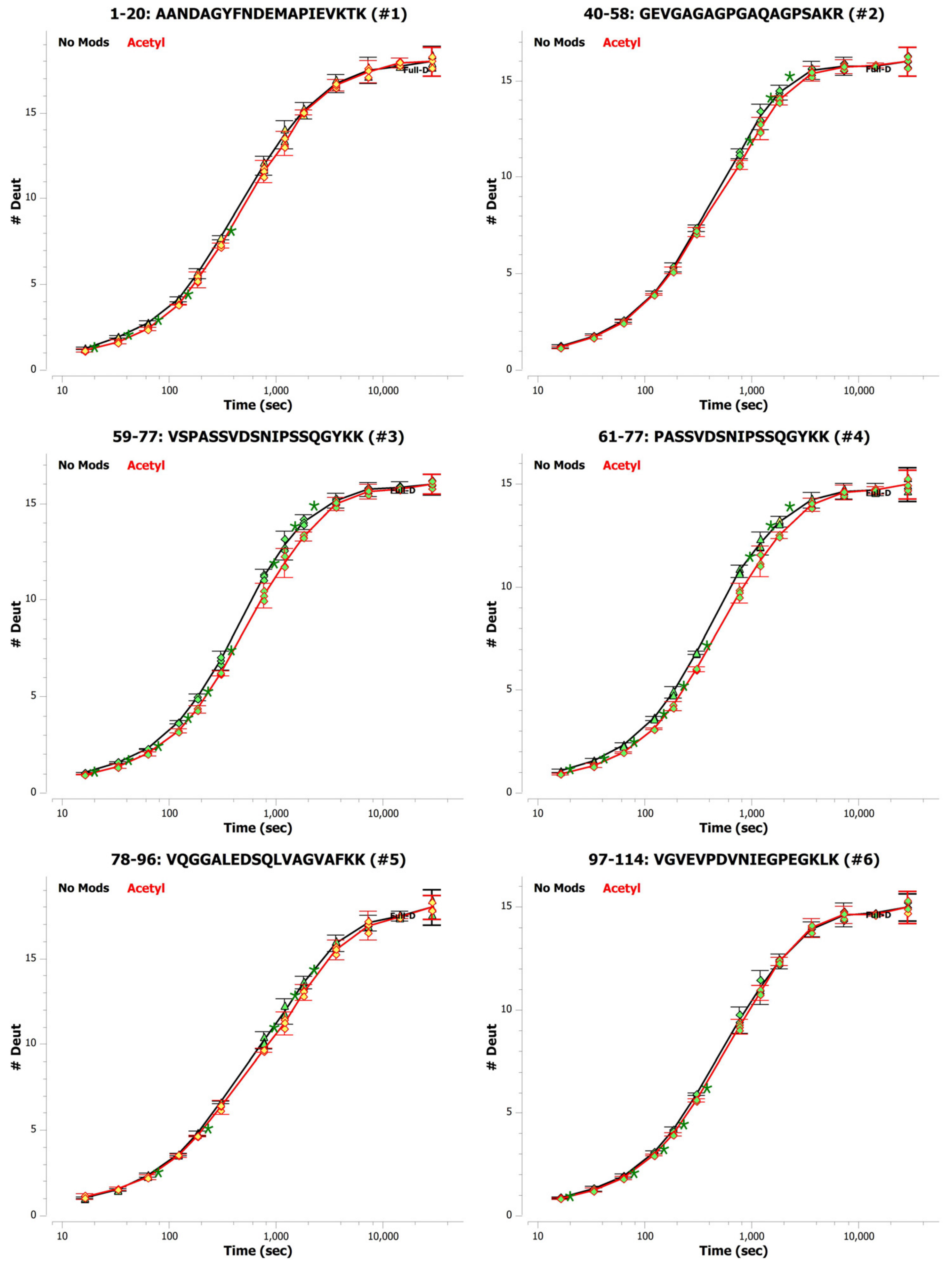

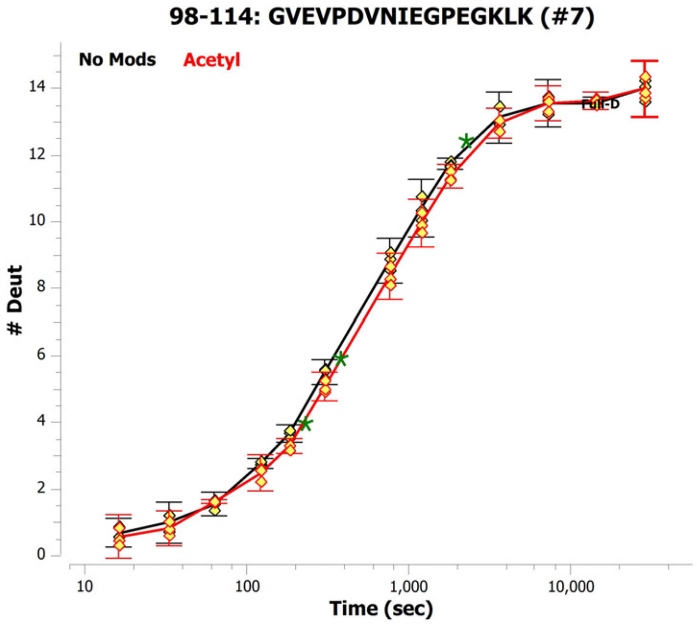
98-114: GVEVPDVNIEGPEGKLK (#7)
No Mods
Acetyl
Full-D
Deut
Time (sec)
0
2
4
6
8
10
12
14
10
100
1,000
10,000

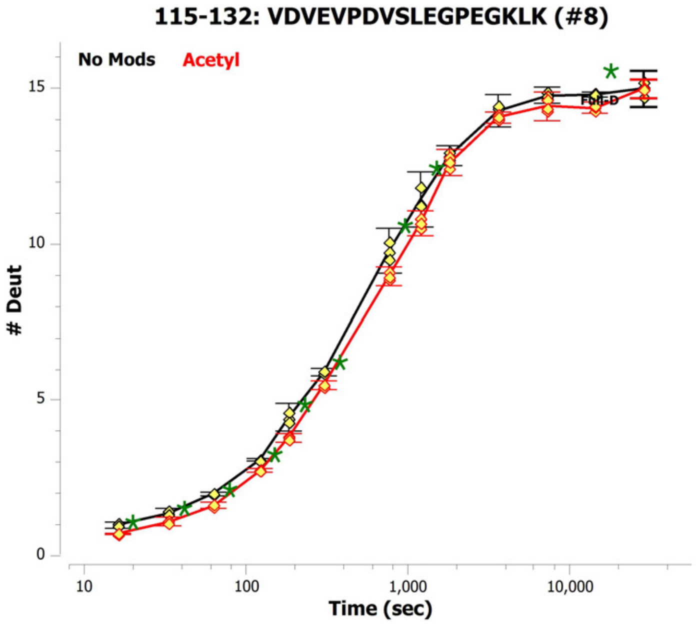
115-132: VDVEVPDVSLEGPEGKLK (#8)
No Mods
Acetyl
Full-D
Deut
Time (sec)
0
5
10
15
10
100
1,000
10,000

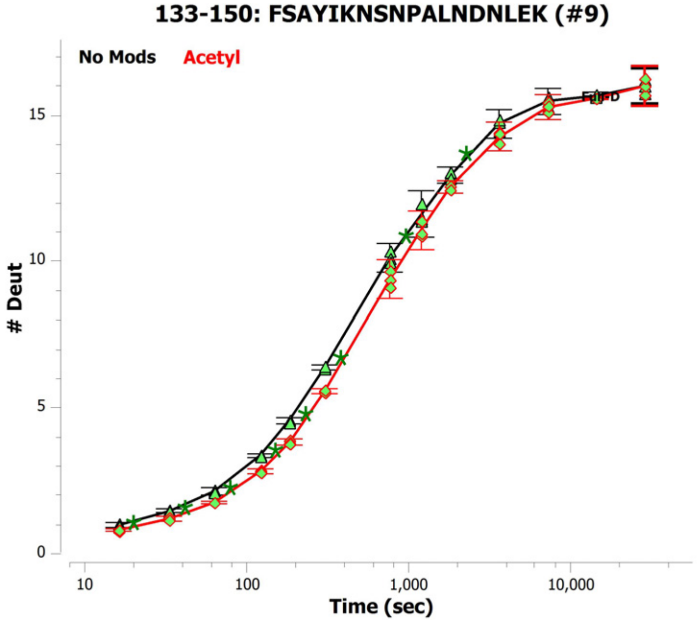
133-150: FSAYIKNSNPALNDNLEK (#9)
No Mods
Acetyl
Full-D
Deut
Time (sec)
0
5
10
15
10
100
1,000
10,000

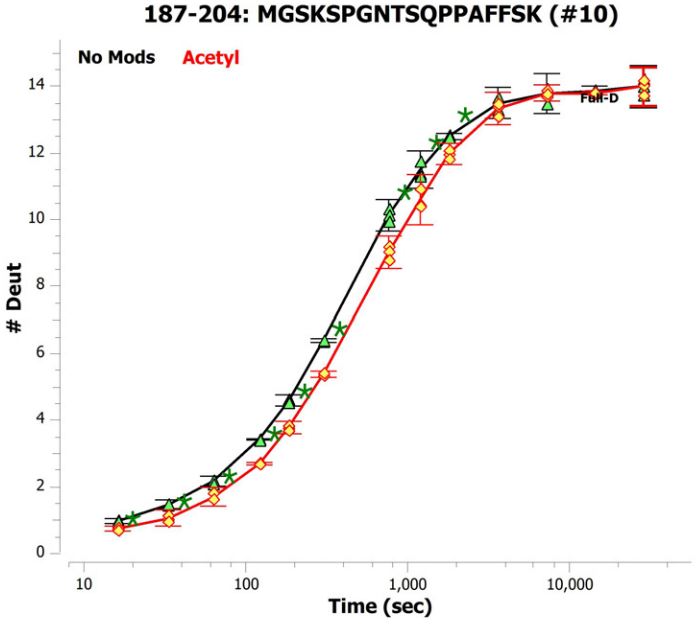
187-204: MGSKSPGNTSQPPAFFSK (#10)
No Mods
Acetyl
Full-D
Deut
Time (sec)
0
2
4
6
8
10
12
14
10
100
1,000
10,000

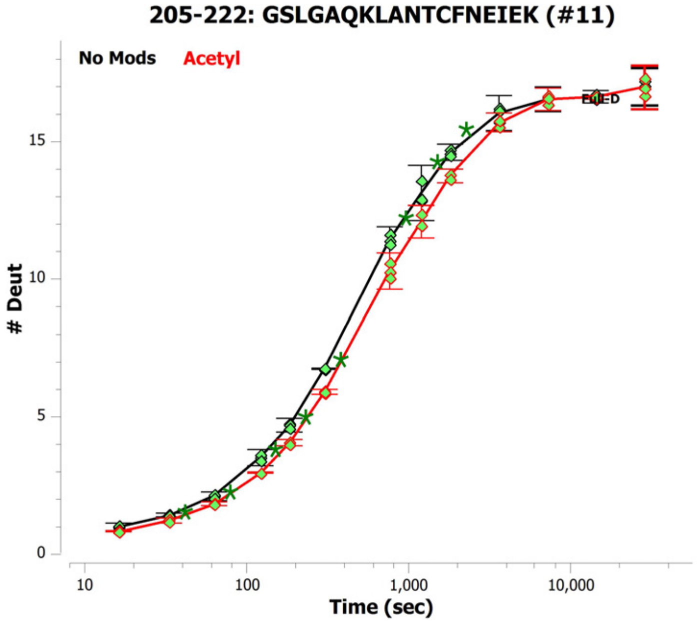
205-222: GSLGAQKLANTCFNEIEK (#11)
No Mods
Acetyl
Full-D
Deut
Time (sec)
0
5
10
15
10
100
1,000
10,000

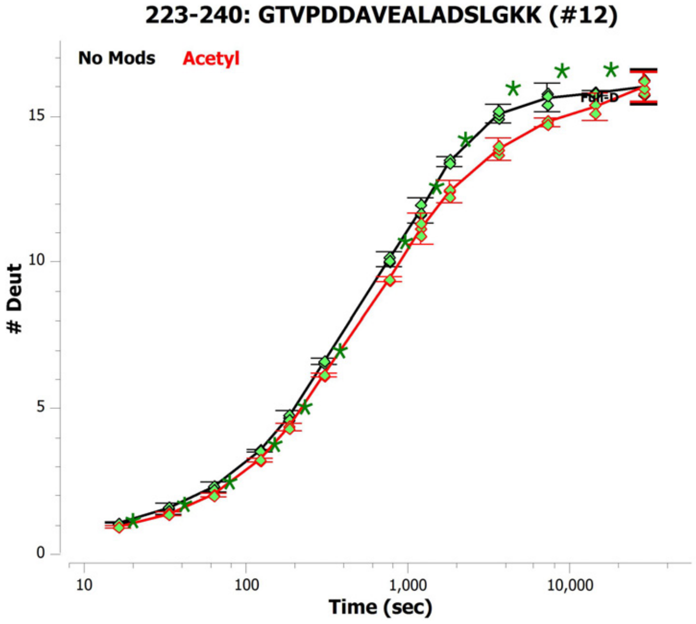
223-240: GTVPDDAVEALADSLGKK (#12)
No Mods
Acetyl
Full-D
Deut
Time (sec)
0
5
10
15
10
100
1,000
10,000

241-258: VNVEAPDVNLEGLGGKLK (#13)

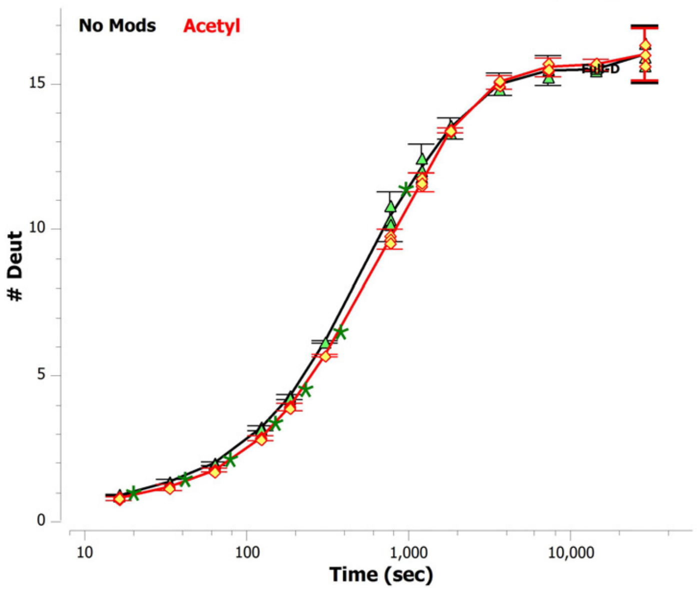

259-275: DASTLQSQKAEGTGDAK (#14)

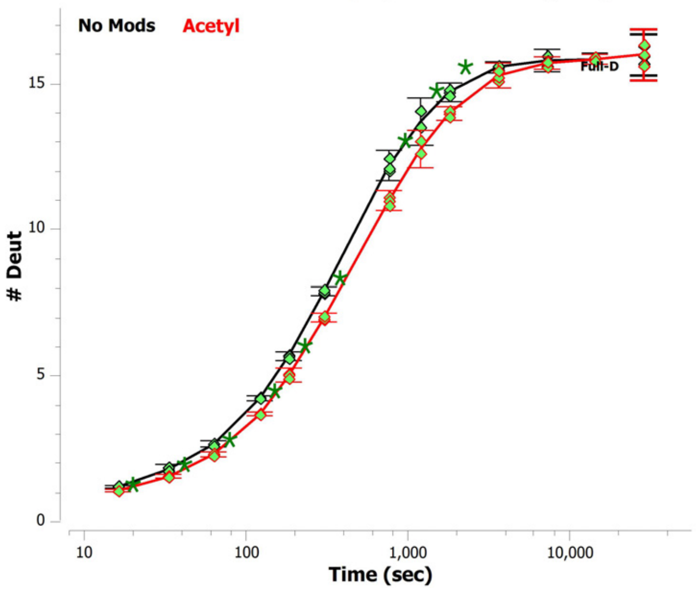

276-292: ANEKTESSSAQQVAVSR (#15)

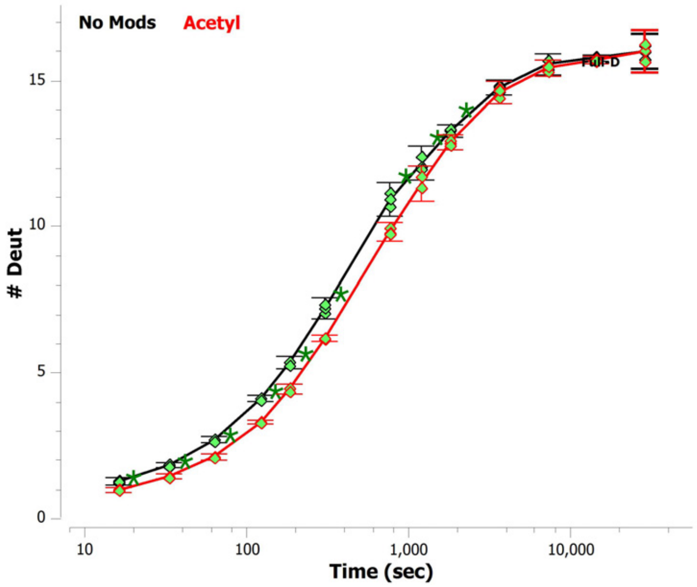

293-309: GTISAPGKVVTAAAQAK (#16)

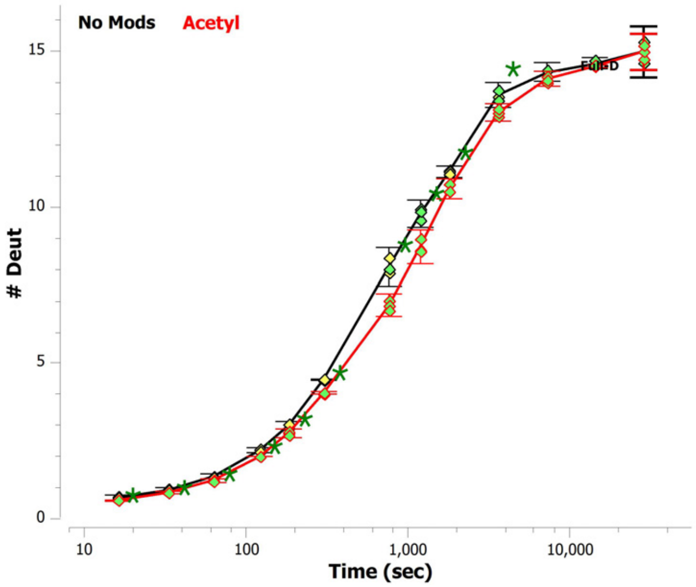

310-326: YEQEHAAIQDKLFQVAK (#17)

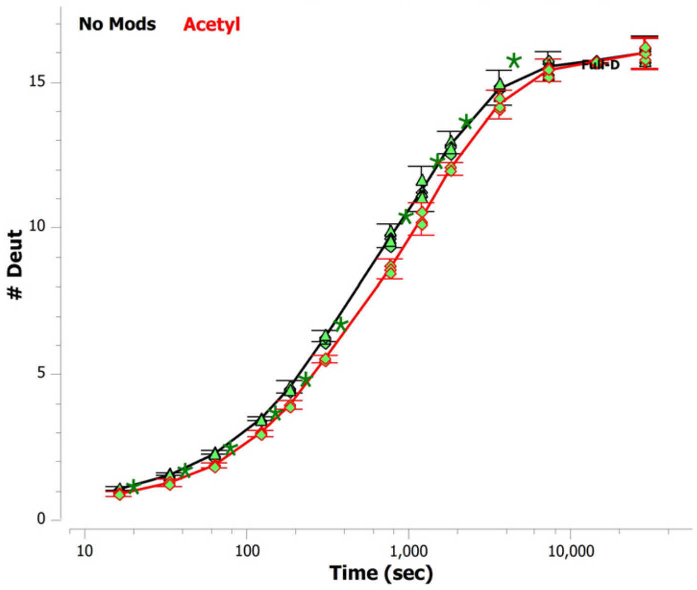

327-343: IINEPTAAAIAYGLDKK (#18)

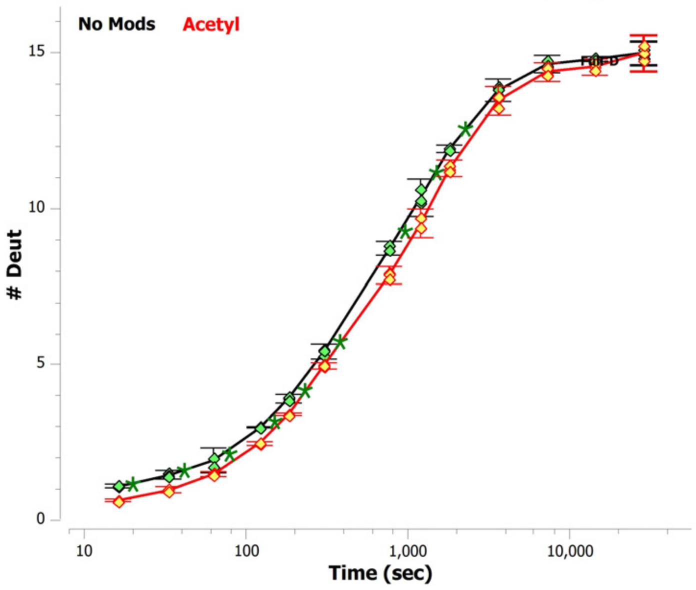

344-359: AANMLQQSGSKNTGAK (#19)

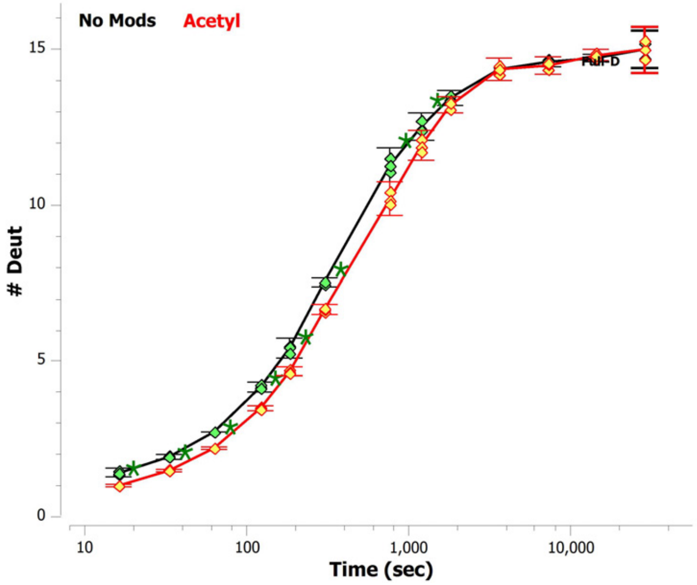

376-391: DYSSGFGGKYGVQADR (#20)

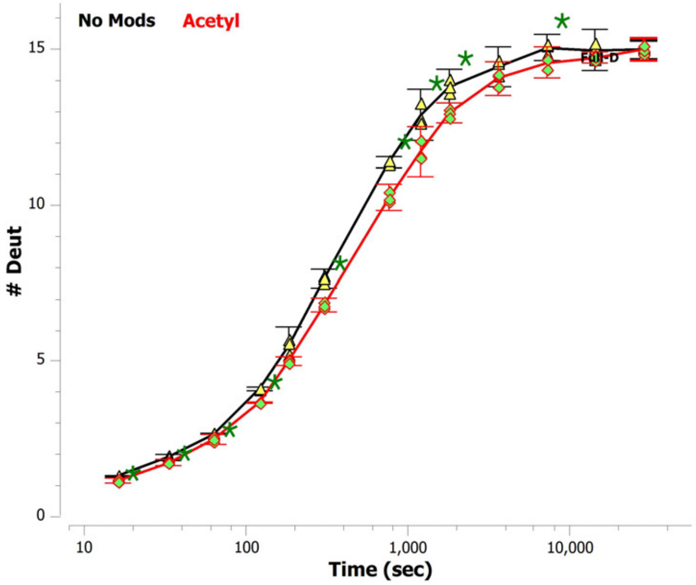

392-407: TGASWTDNIMAQKCSK (#21)

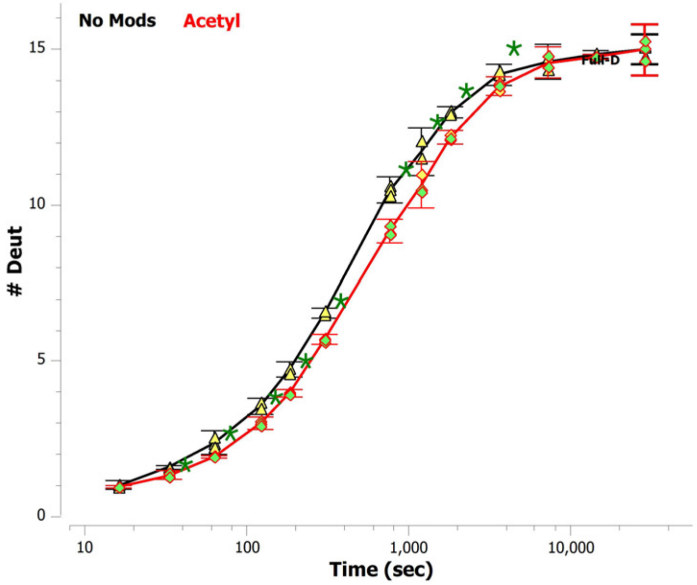

408-423: GWLKSNVSDAVAQSTR (#22)

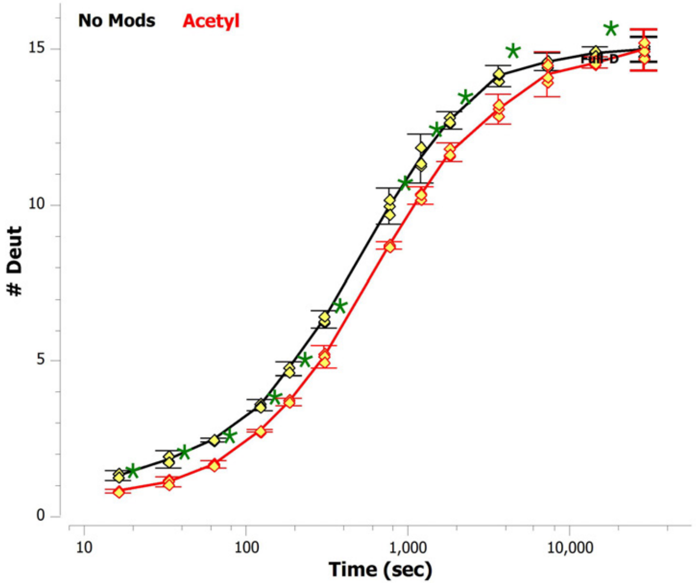

424-439: VLTANSNPSSPSAAKR (#23)

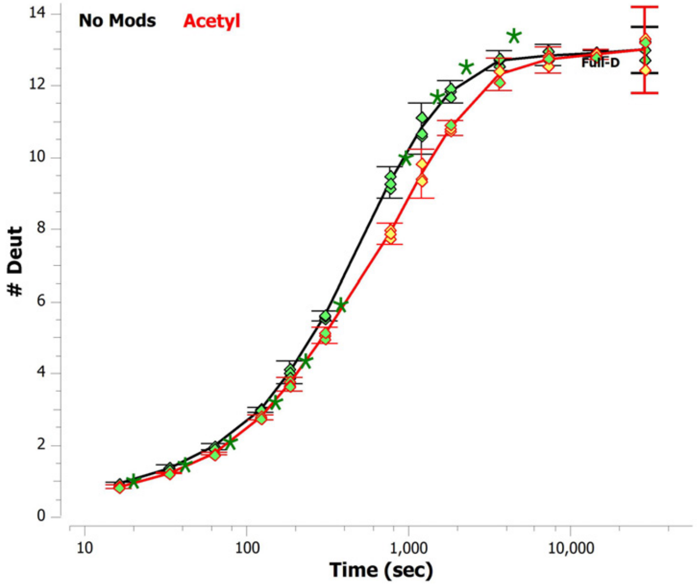

440-454: DYSAPVNFISAGLKK (#24)

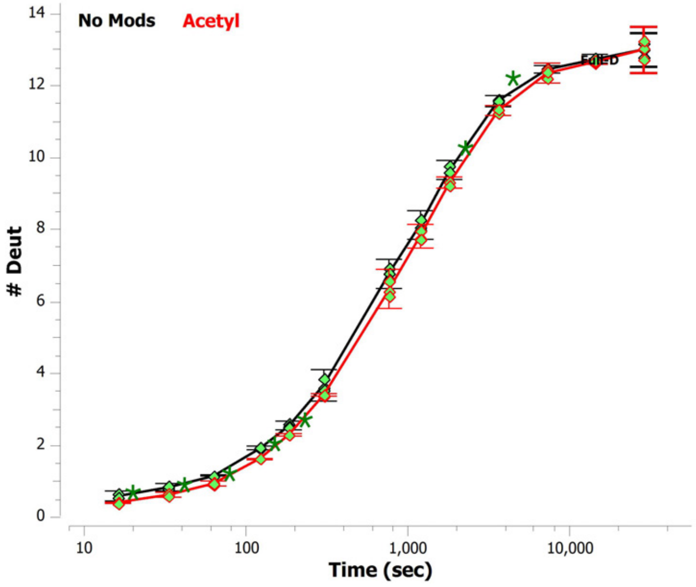

455-469: SSGPGGQNVNKVNSK (#25)

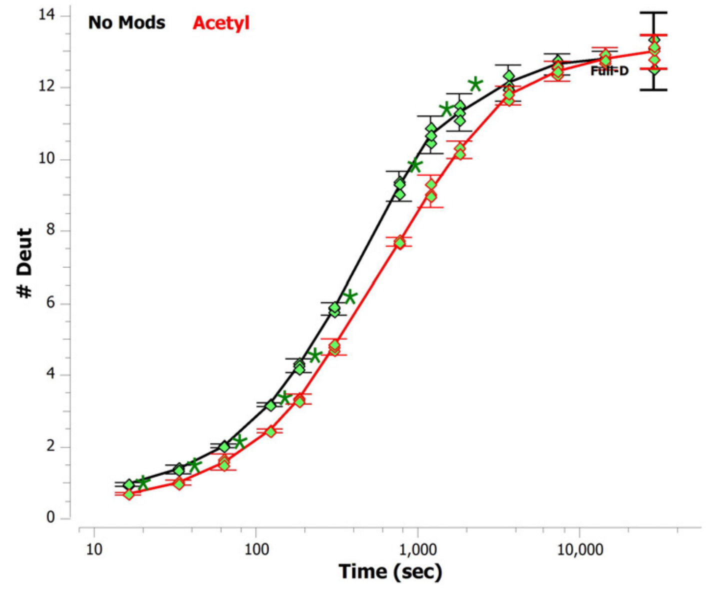

456-469: SGPGGQNVNKVNSK (#26)

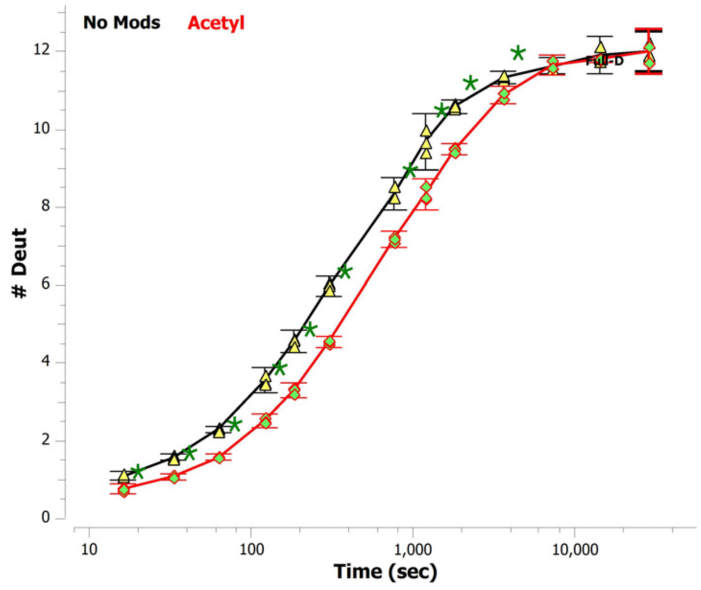

470-484: SSVACKWNLAEAQQK (#27)

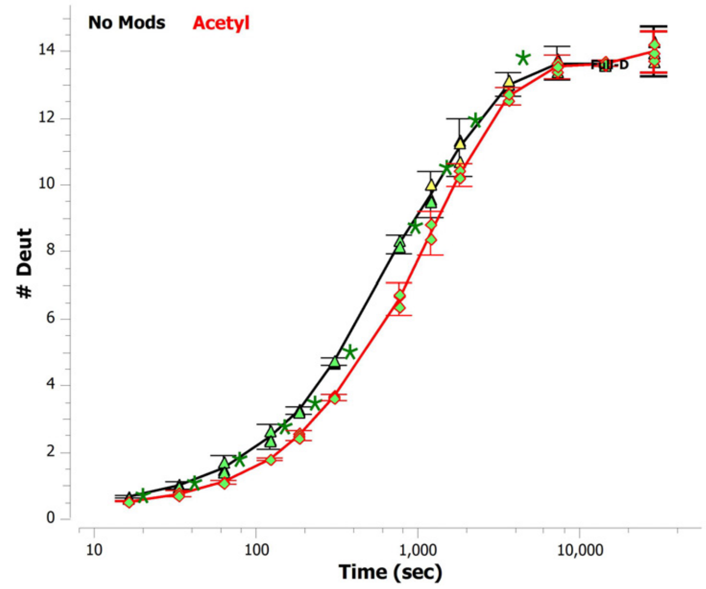

500-514: TEDEVLTSKGDAWAK (#28)

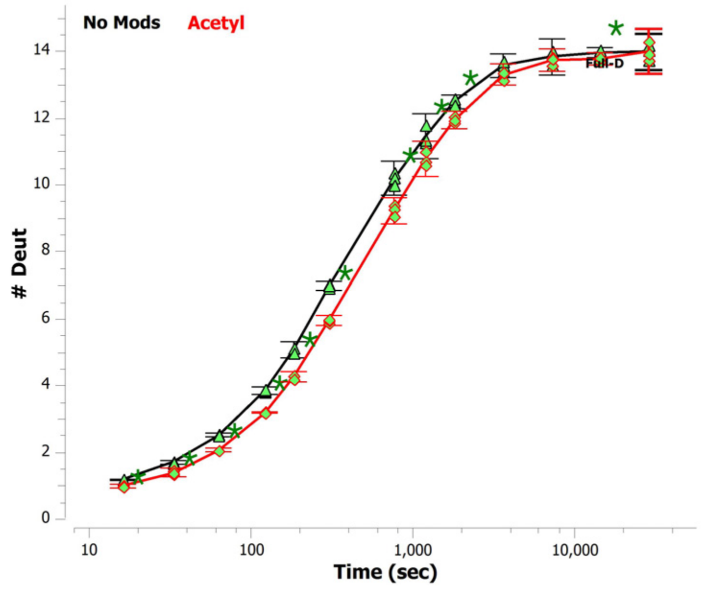

515-529: TQAYQDQKPGTSGLR (#29)

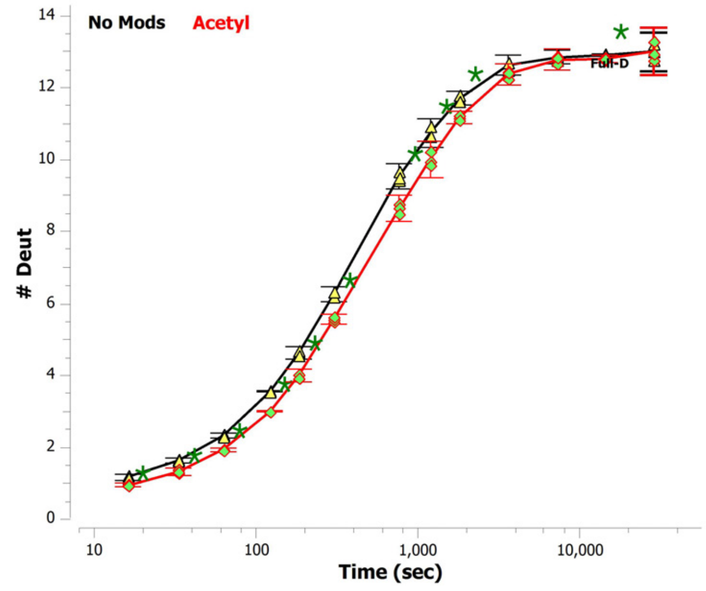

530-544: GFALVGVGSEASSKK (#30)

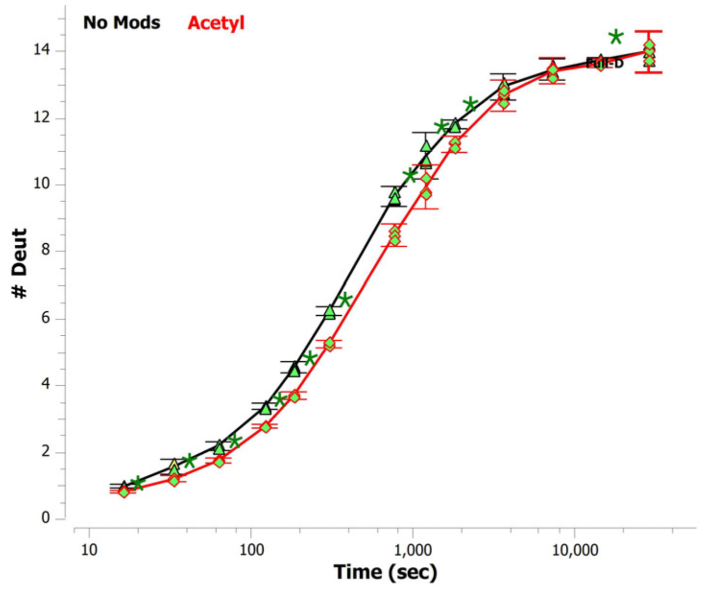

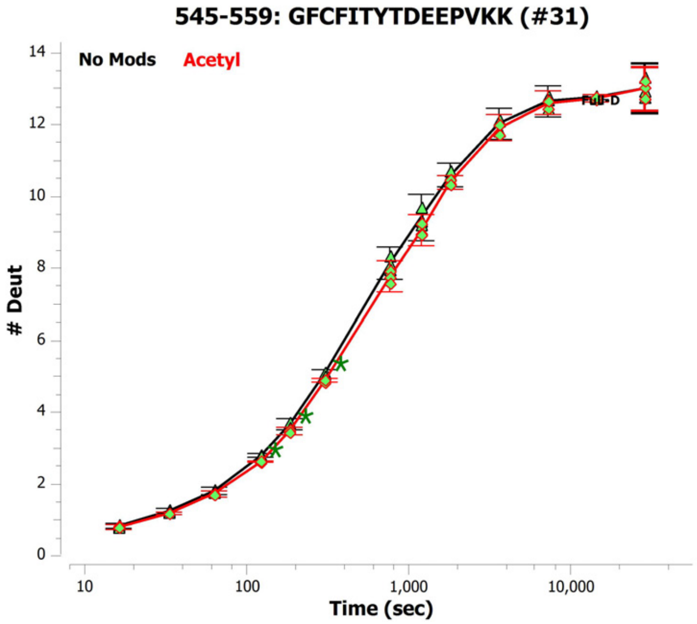
545-559: GFCFITYTDEEPVKK (#31)
No Mods
Acetyl
Deut
Time (sec)
Full-D

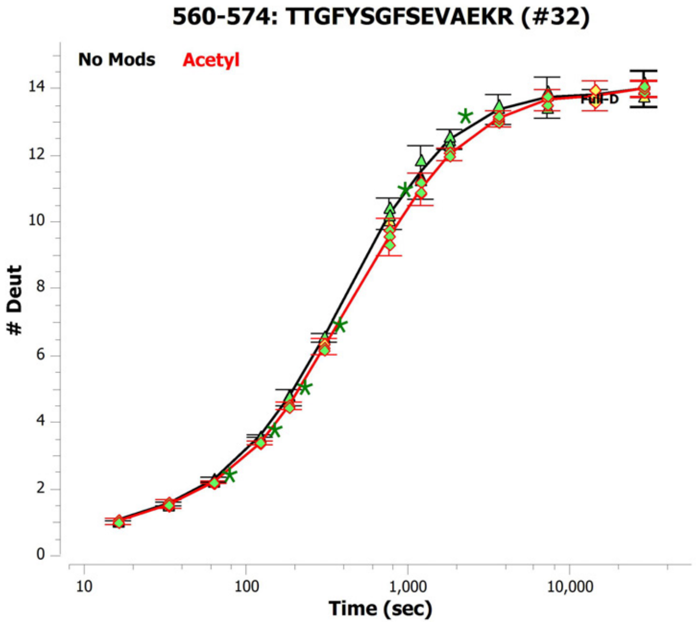
560-574: TTGFYSGFSEVAEKR (#32)
No Mods
Acetyl
Deut
Time (sec)
Full-D

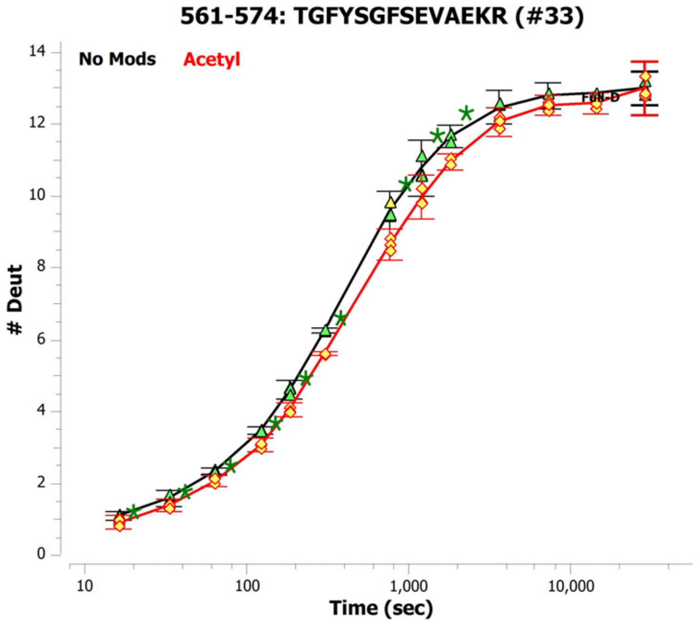
561-574: TGFYSGFSEVAEKR (#33)
No Mods
Acetyl
Deut
Time (sec)
Full-D

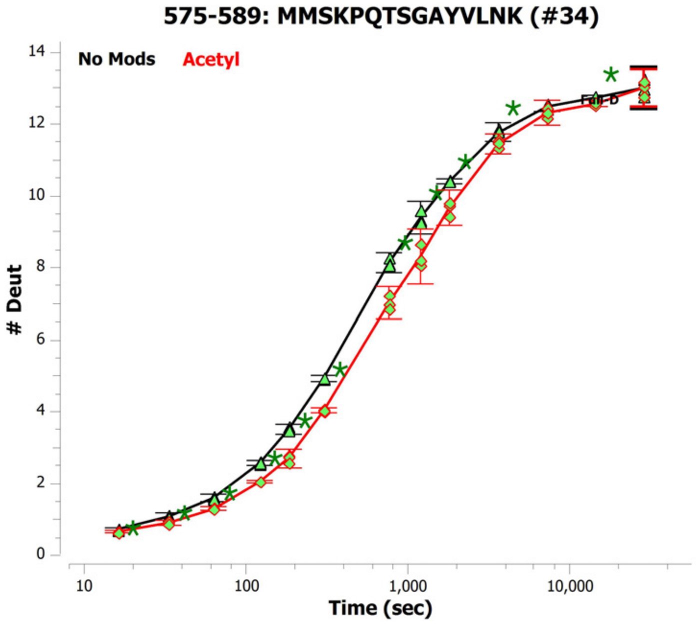
575-589: MMSKPQTSGAYVLNK (#34)
No Mods
Acetyl
Deut
Time (sec)
Full-D

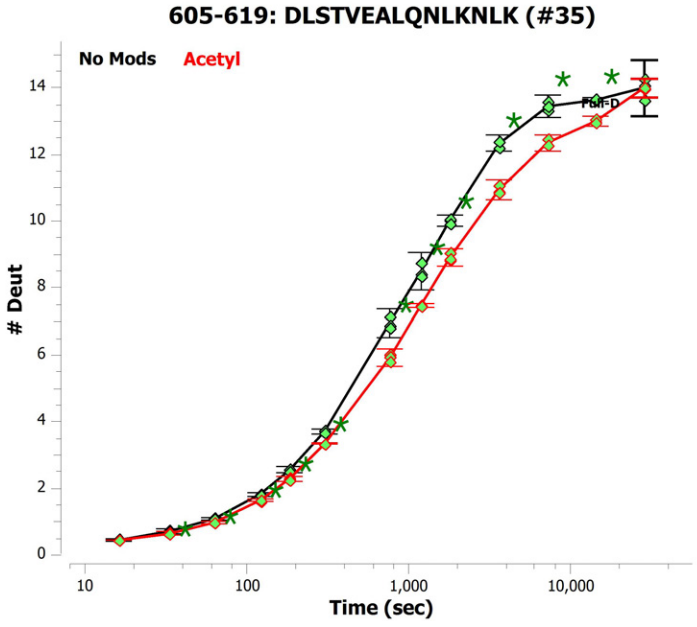
605-619: DLSTVEALQNLKNLK (#35)
No Mods
Acetyl
Deut
Time (sec)
Full-D

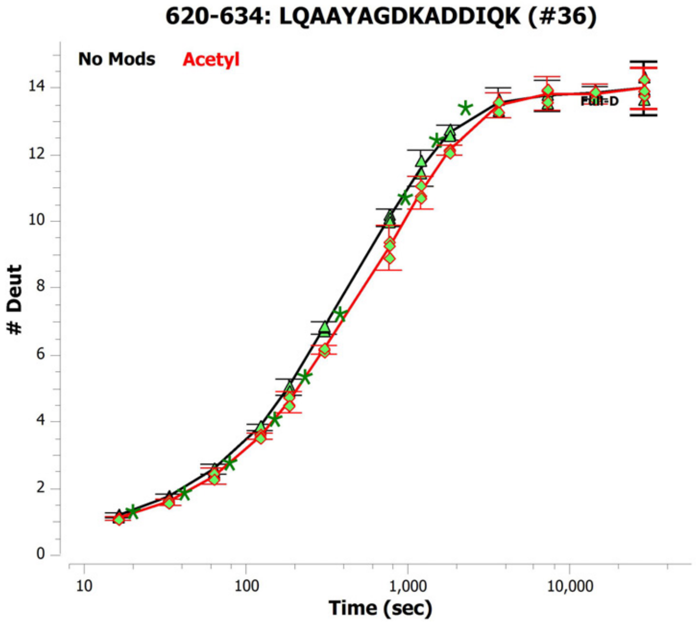
620-634: LQAAYAGDKADDIQK (#36)
No Mods
Acetyl
Deut
Time (sec)
Full-D

635-649: PSVGSQSNQAGQGKR (#37)

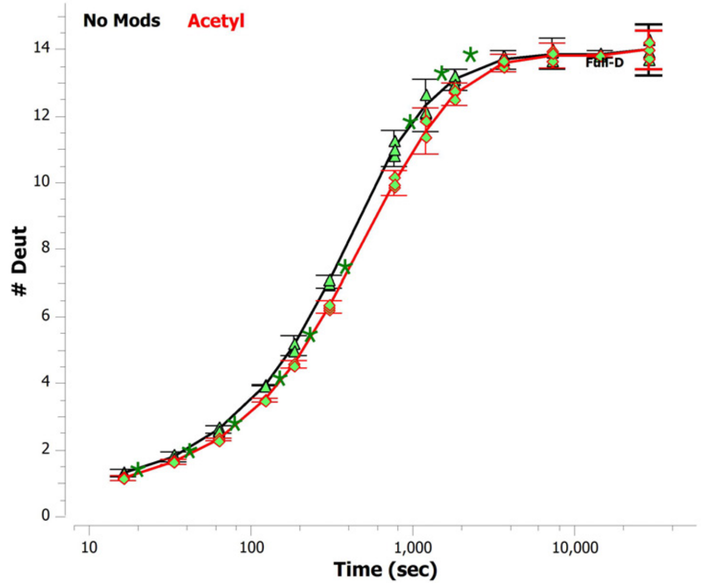

665-679: DTMGIADKTENTLER (#38)

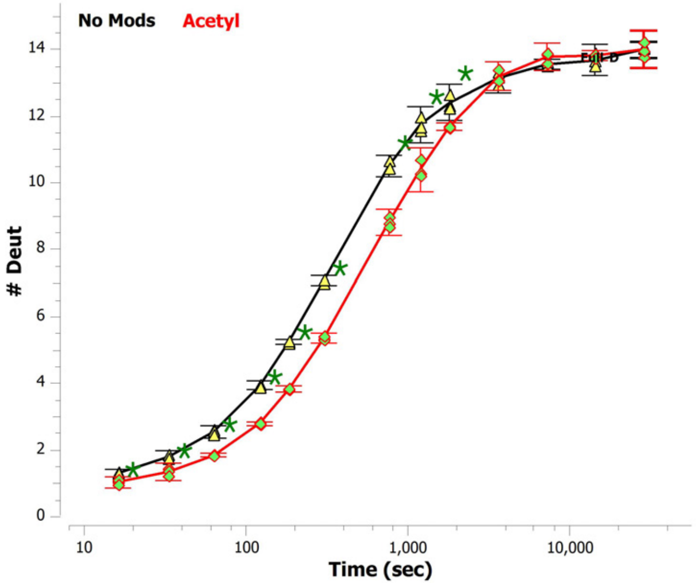

680-694: DTPTSAGPNSFNKGK (#39)

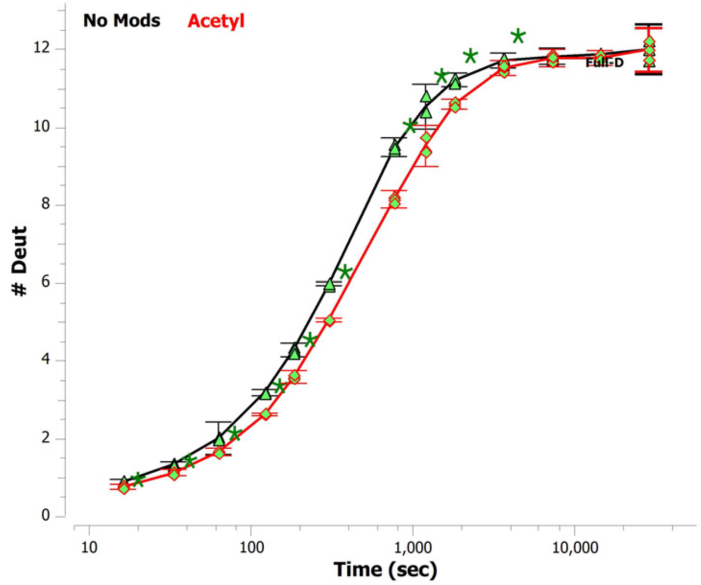

695-708: AGPNASIISLKSDK (#40)

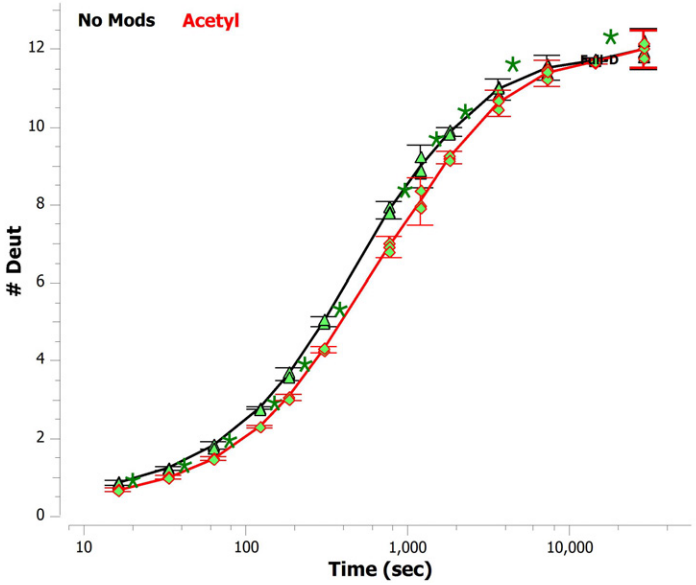

709-722: SDSGKPYYYNSQTK (#41)

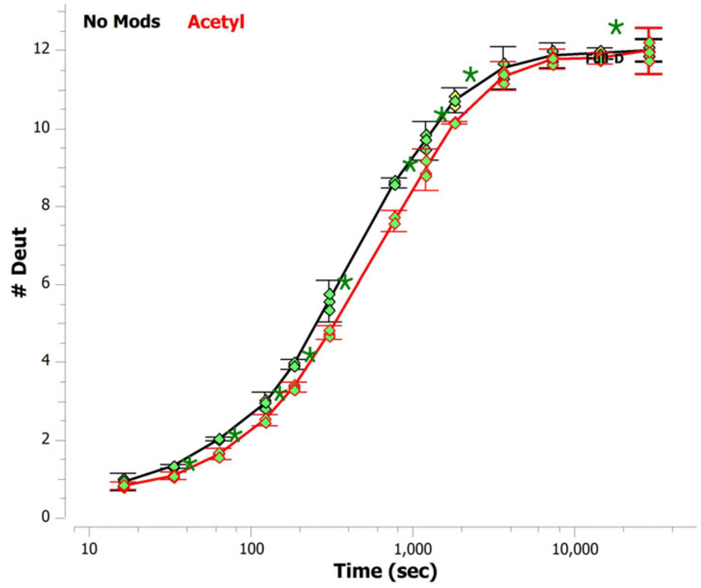

723-736: ITESVAETAQTIKK (#42)

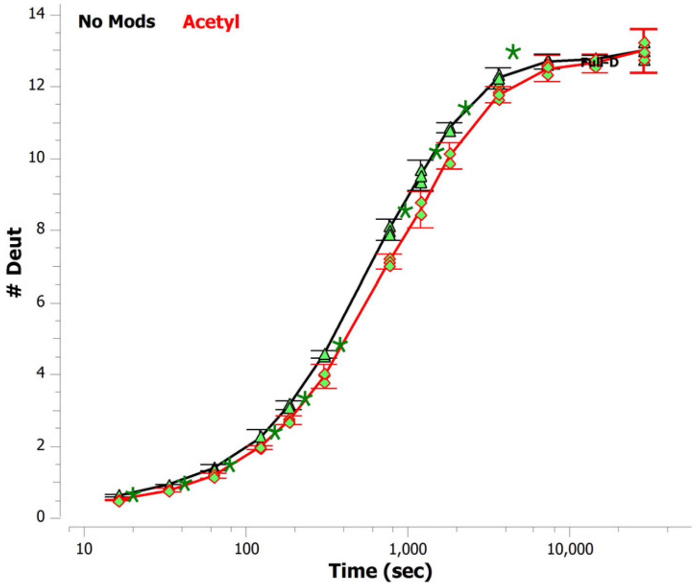

724-736: TESVAETAQTIKK (#43)

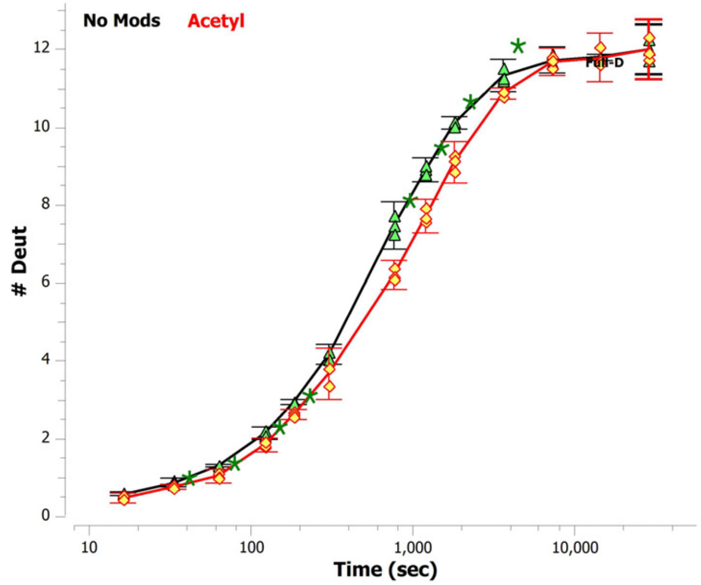

737-750: SGNFSAAMKDLSGK (#44)

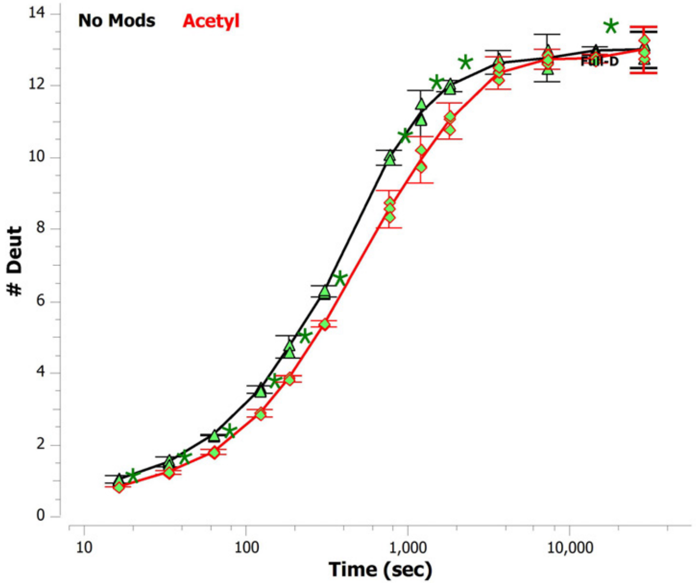

751-764: DDSFLGKLGGTLAR (#45)

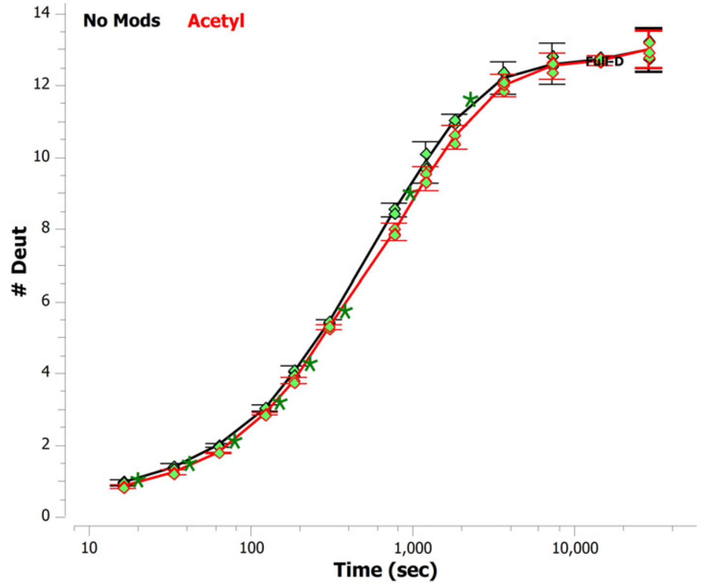

765-778: DESFLGKLGGTLAR (#46)

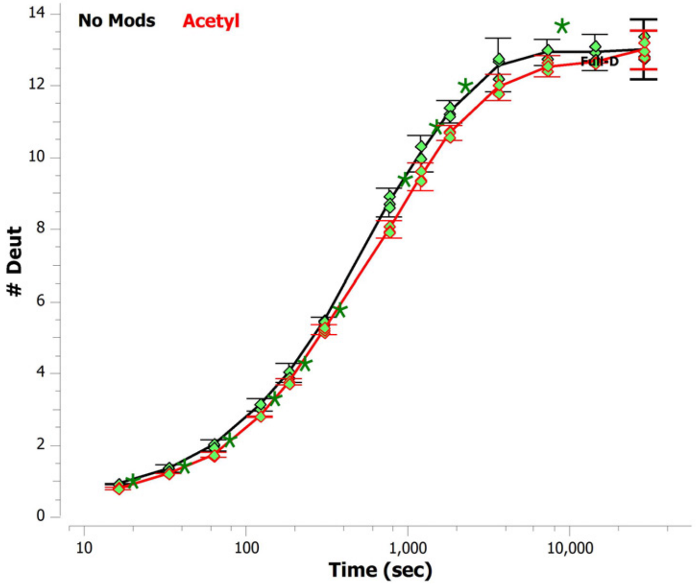

779-792: IVSGKDYNVTANSK (#47)

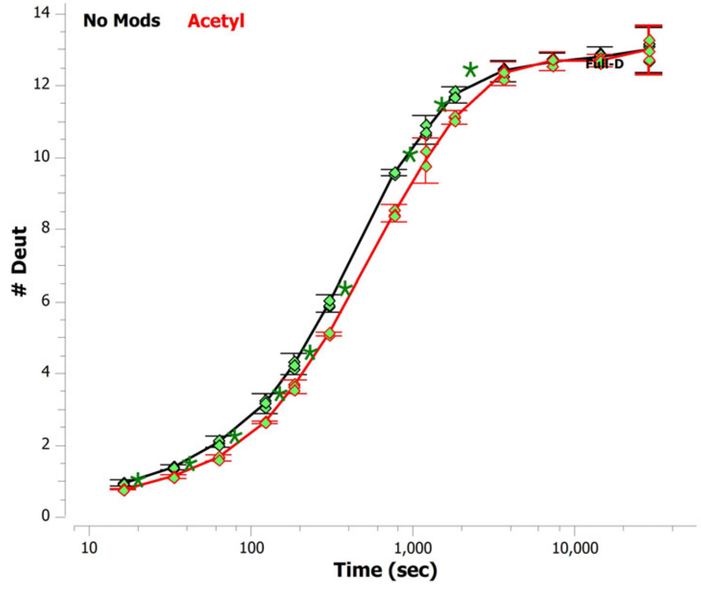

793-806: TVSKVDDFLANEAK (#48)

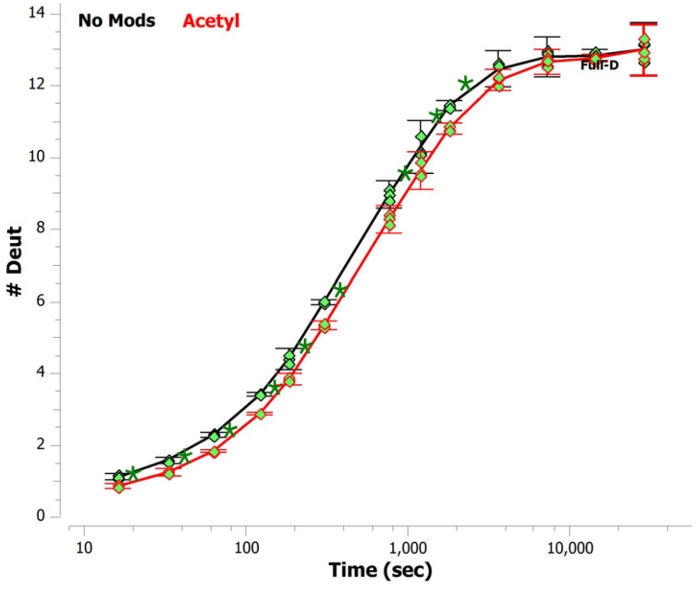

807-820: FLQEFYQDDELGKK (#49)

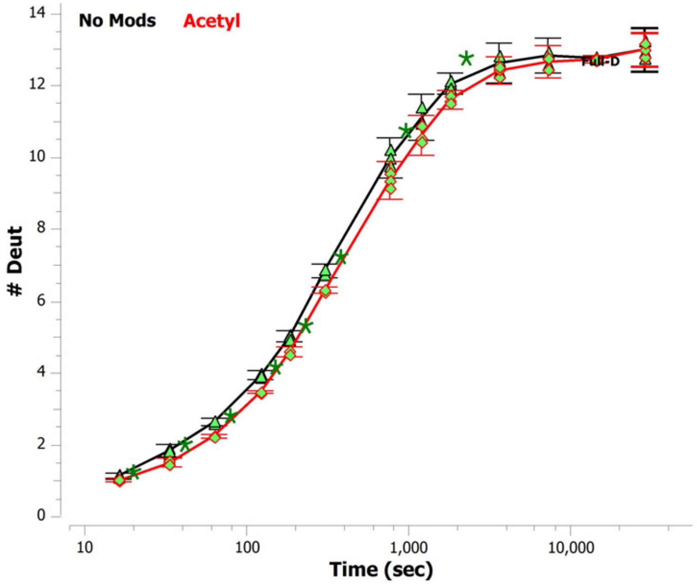

821-834: PANEKATDDYHYEK (#50)

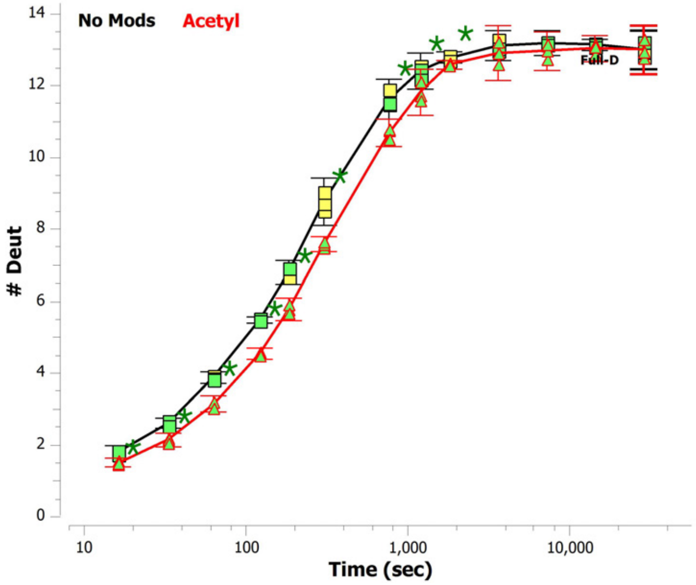

835-848: PSKGPLQSVQVFGR (#51)

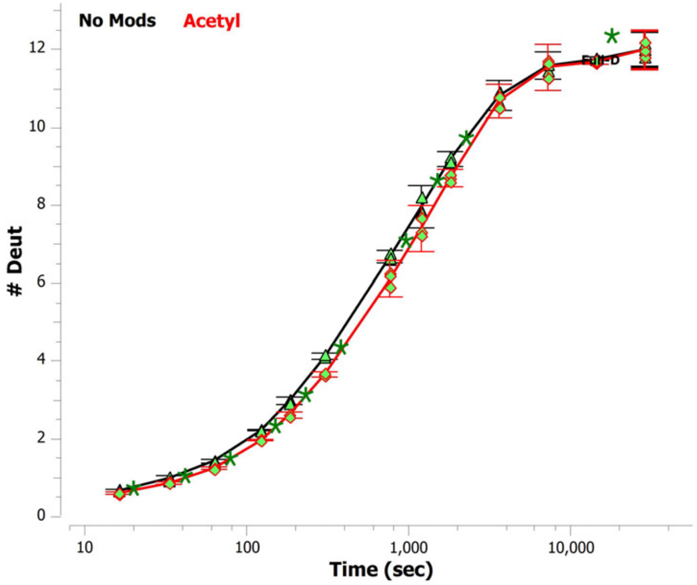

849-862: ISSNFSSIIAEKLR (#52)

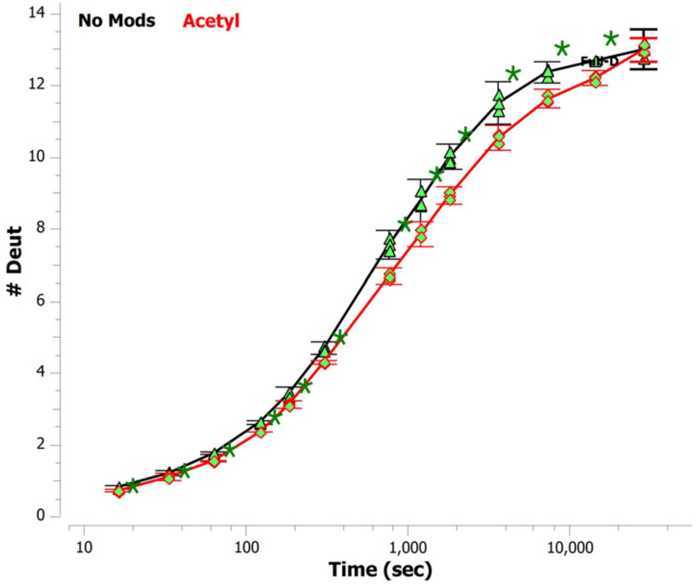

877-890: GTITVSAQELKDNR (#53)

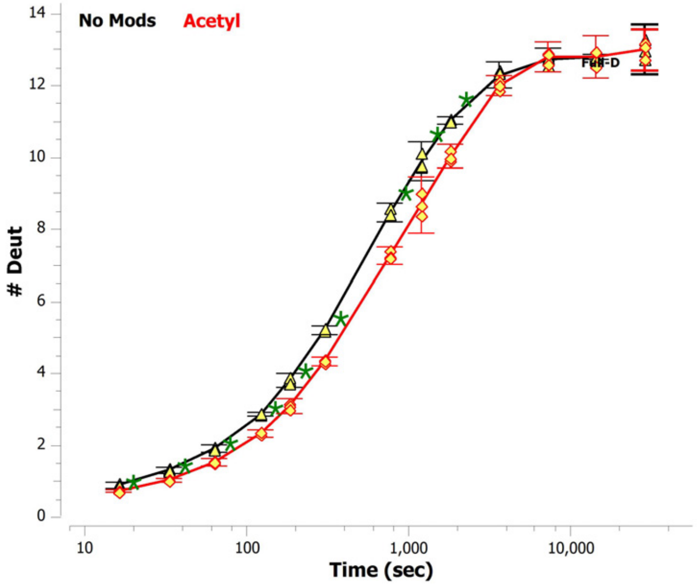

891-903: AGQKLIDVNHYAK (#54)

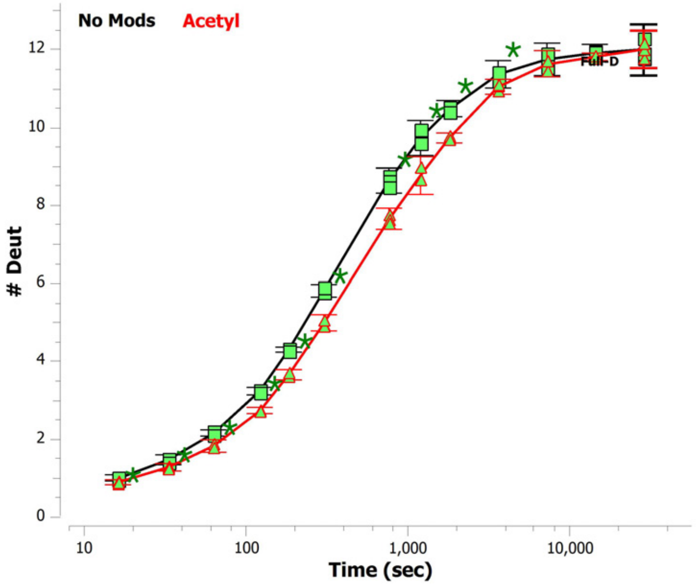

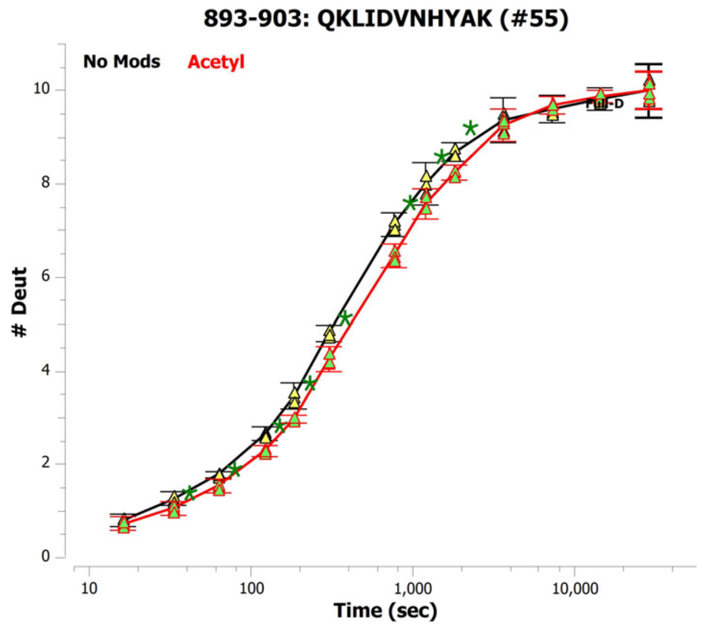
893-903: QKLIDVNHYAK (#55)
No Mods
Acetyl
Full-D
Deut
0
2
4
6
8
10
Time (sec)
10
100
1,000
10,000

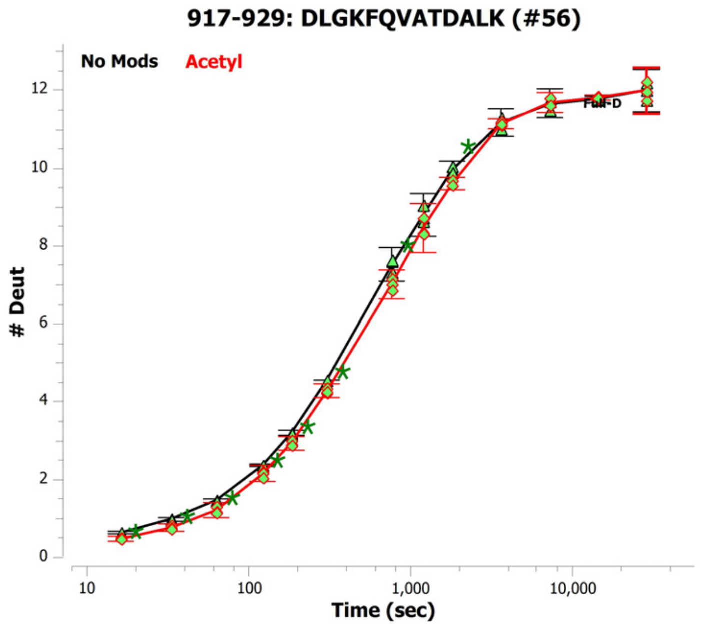
917-929: DLGKFQVATDALK (#56)
No Mods
Acetyl
Full-D
Deut
0
2
4
6
8
10
12
Time (sec)
10
100
1,000
10,000

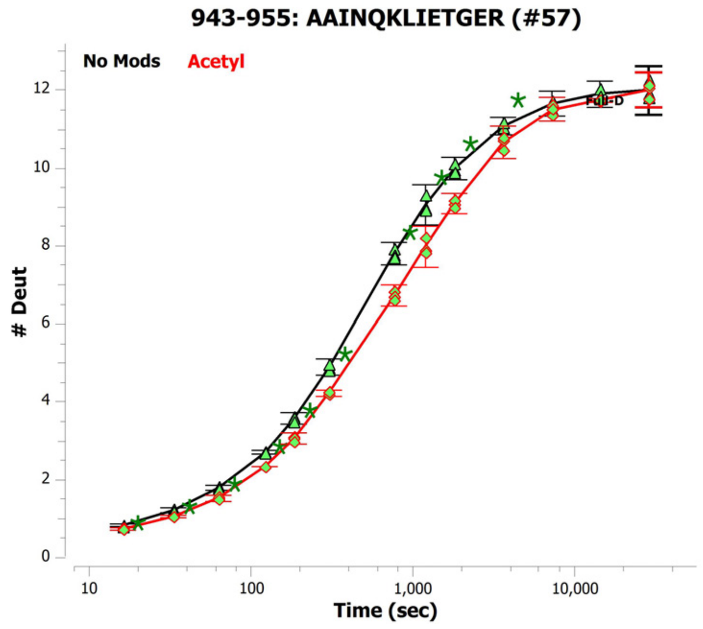
943-955: AAINQKLIETGER (#57)
No Mods
Acetyl
Full-D
Deut
0
2
4
6
8
10
12
Time (sec)
10
100
1,000
10,000

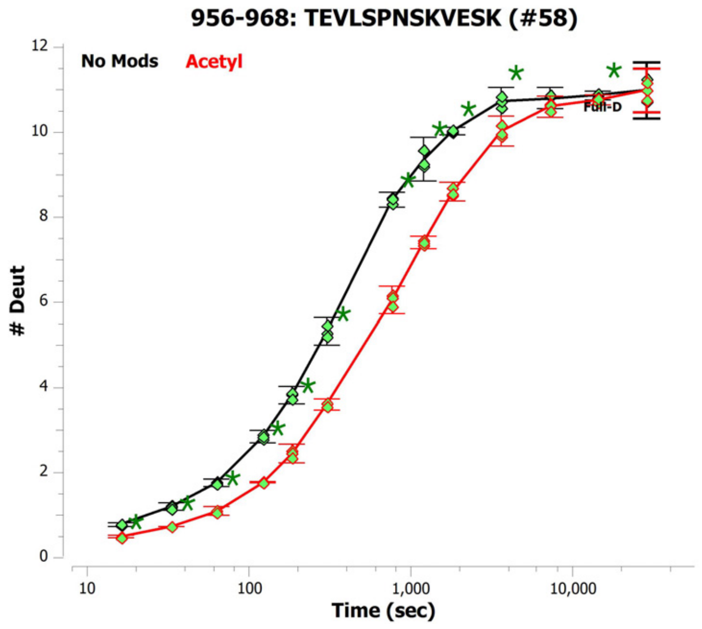
956-968: TEVLSPNSKVESK (#58)
No Mods
Acetyl
Full-D
Deut
0
2
4
6
8
10
12
Time (sec)
10
100
1,000
10,000

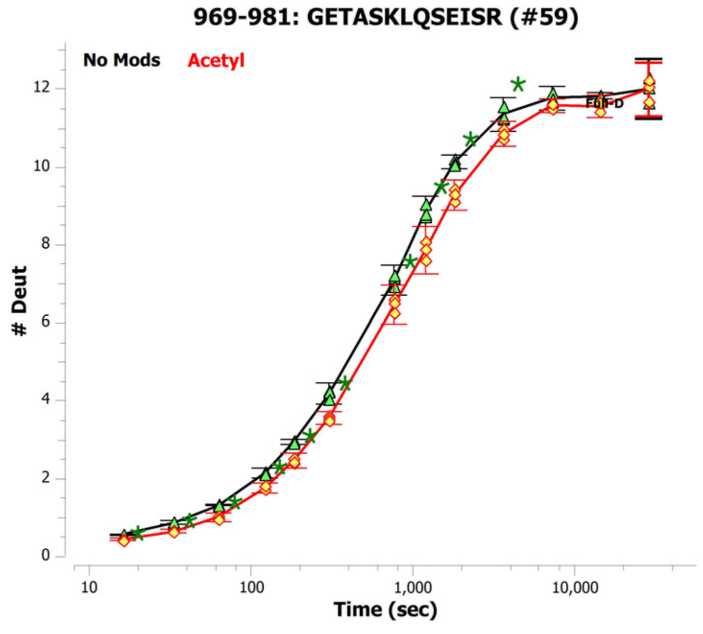
969-981: GETASKLQSEISR (#59)
No Mods
Acetyl
Full-D
Deut
0
2
4
6
8
10
12
Time (sec)
10
100
1,000
10,000

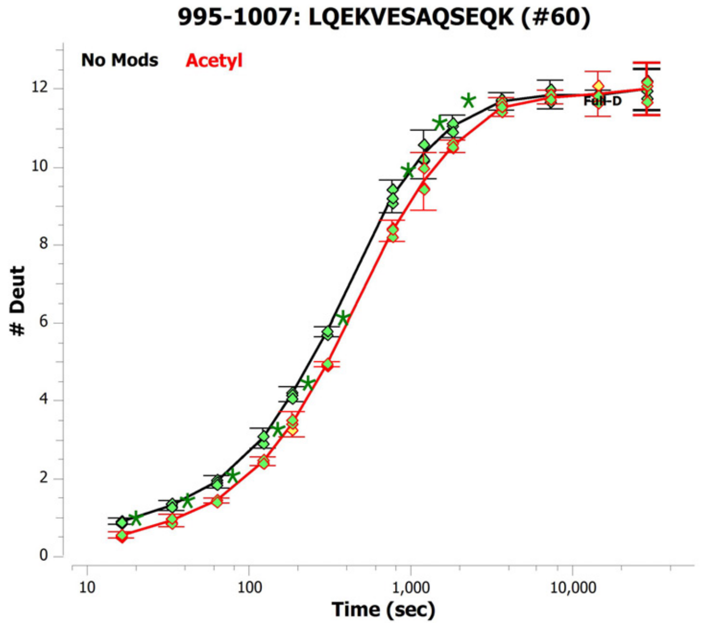
995-1007: LQEKVESAQSEQK (#60)
No Mods
Acetyl
Full-D
Deut
0
2
4
6
8
10
12
Time (sec)
10
100
1,000
10,000

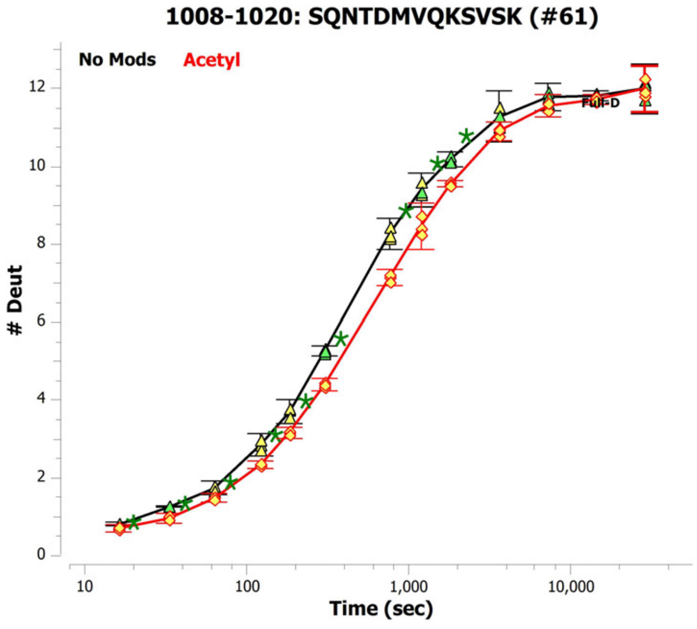
1008-1020: SQNTDMVQKSVSK (#61)
No Mods
Acetyl
Full-D
Deut
Time (sec)

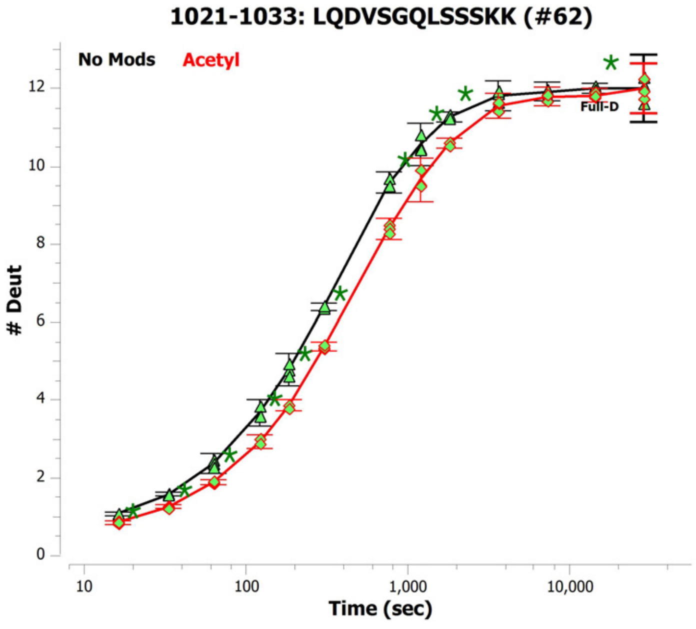
1021-1033: LQDVSGQLSSSKK (#62)
No Mods
Acetyl
Full-D
Deut
Time (sec)

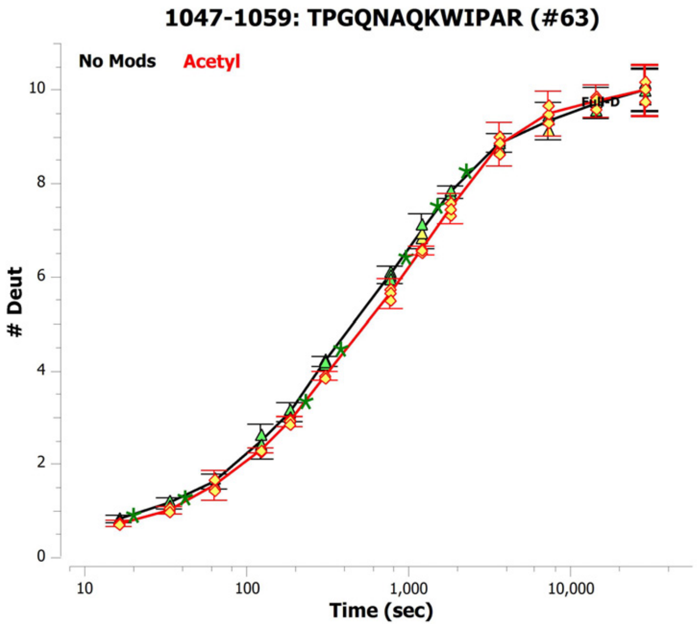
1047-1059: TPGQNAQKWIPAR (#63)
No Mods
Acetyl
Full-D
Deut
Time (sec)

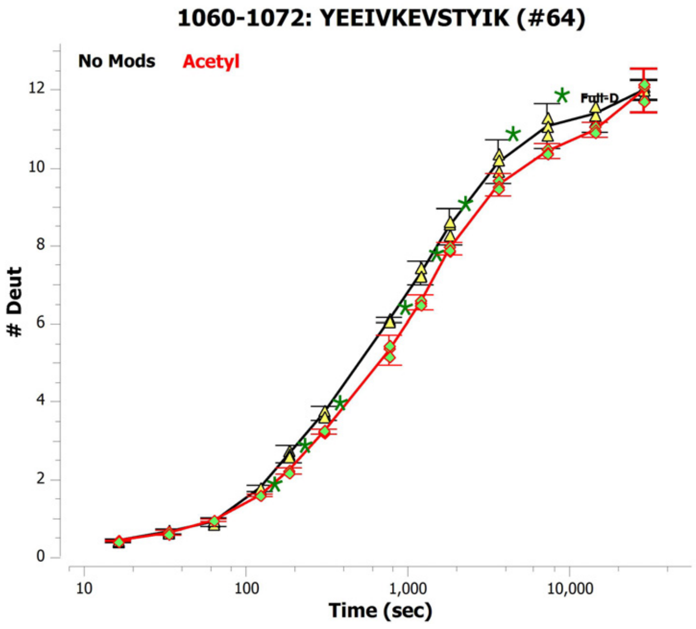
1060-1072: YEEIVKEVSTYIK (#64)
No Mods
Acetyl
Full-D
Deut
Time (sec)

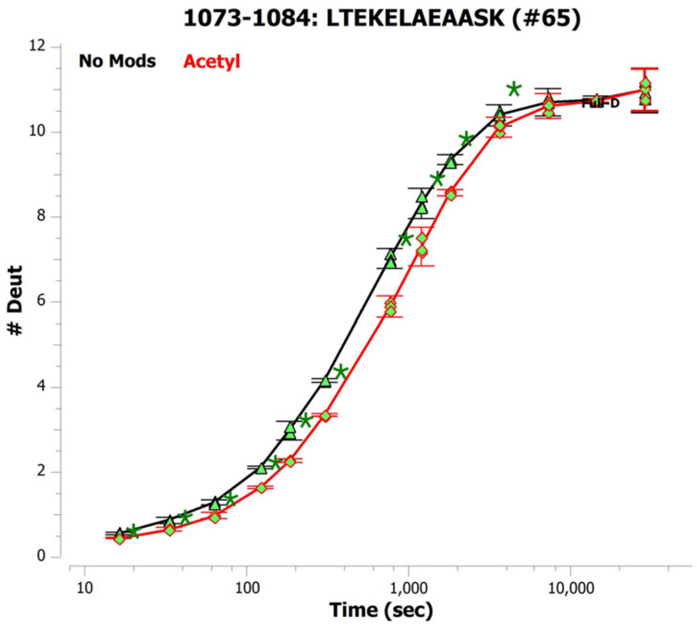
1073-1084: LTEKELAEAASK (#65)
No Mods
Acetyl
Full-D
Deut
Time (sec)

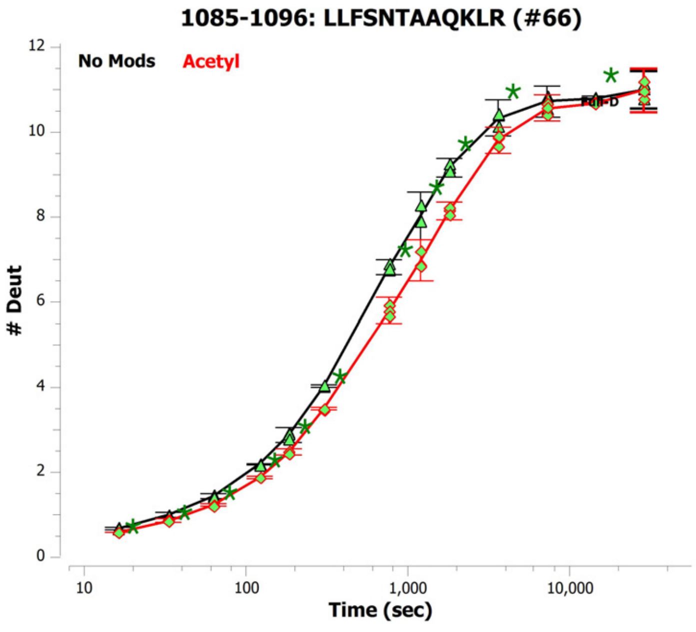
1085-1096: LLFSNTAAQKLR (#66)
No Mods
Acetyl
Full-D
Deut
Time (sec)

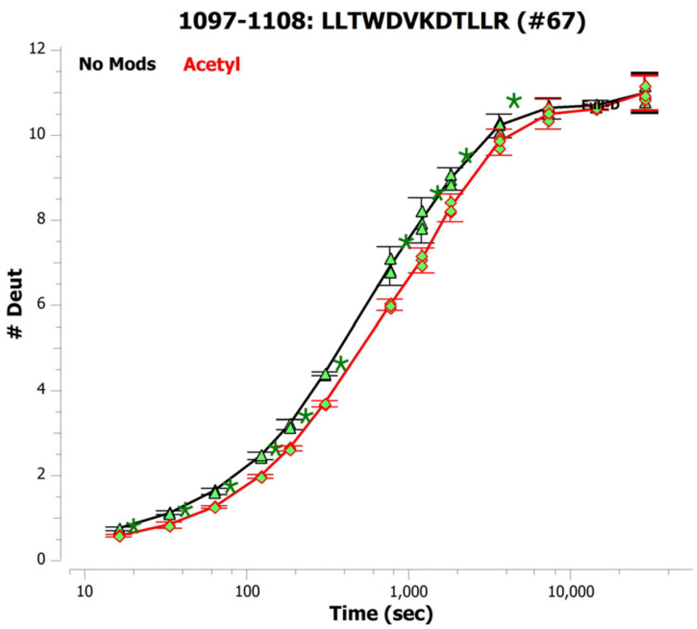
1097-1108: LLTWDVKDTLLR (#67)
No Mods
Acetyl
Deut
Time (sec)
FullD
0
2
4
6
8
10
12
10
100
1,000
10,000

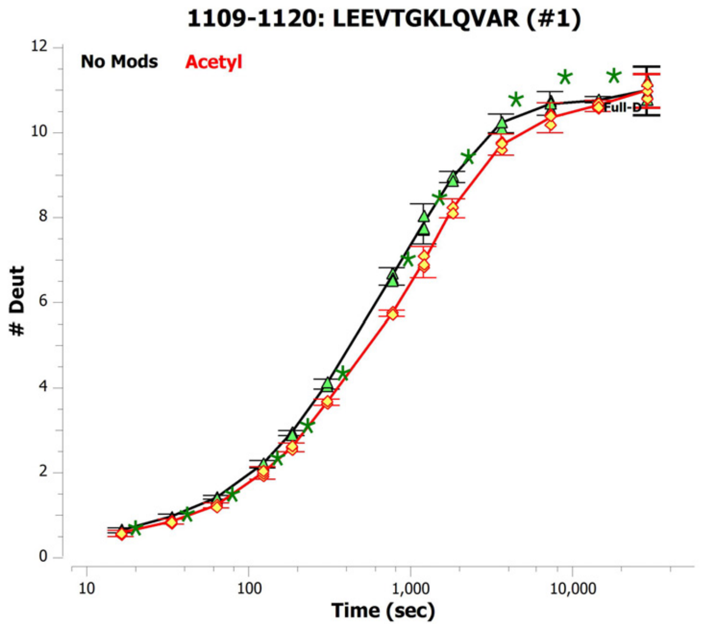
1109-1120: LEEVTGKLQVAR (#1)
No Mods
Acetyl
Full-D
Deut
Time (sec)
0
2
4
6
8
10
12
10
100
1,000
10,000

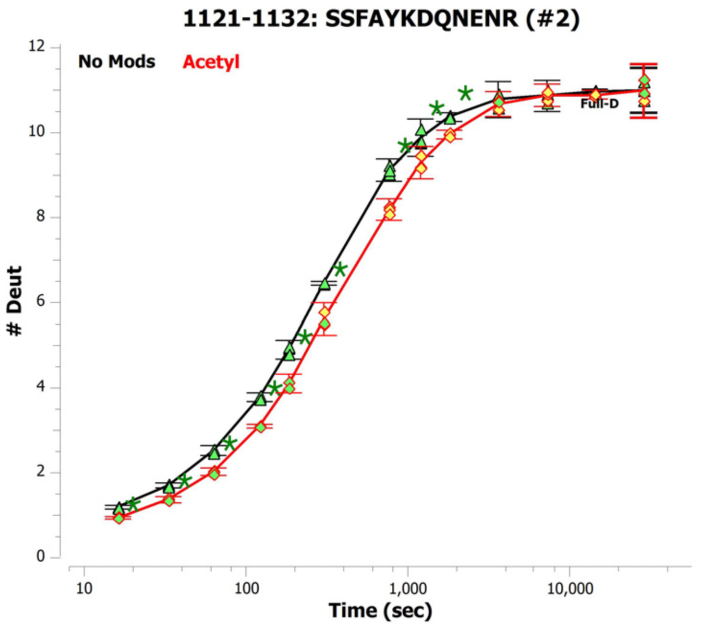
1121-1132: SSFAYKDQNENR (#2)
No Mods
Acetyl
Full-D
Deut
Time (sec)
0
2
4
6
8
10
12
10
100
1,000
10,000

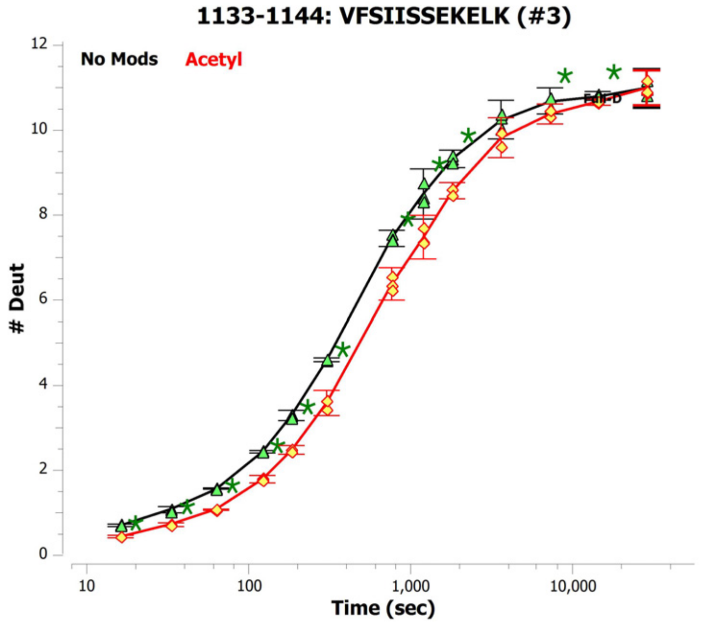
1133-1144: VFSIISSEKELK (#3)
No Mods
Acetyl
Full-D
Deut
Time (sec)
0
2
4
6
8
10
12
10
100
1,000
10,000

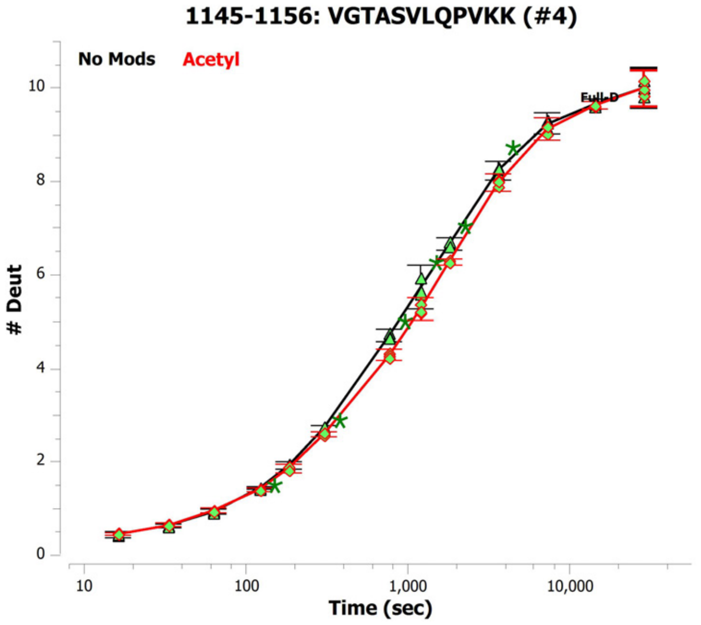
1145-1156: VGTASVLQPVKK (#4)
No Mods
Acetyl
Full-D
Deut
Time (sec)
0
2
4
6
8
10
10
100
1,000
10,000

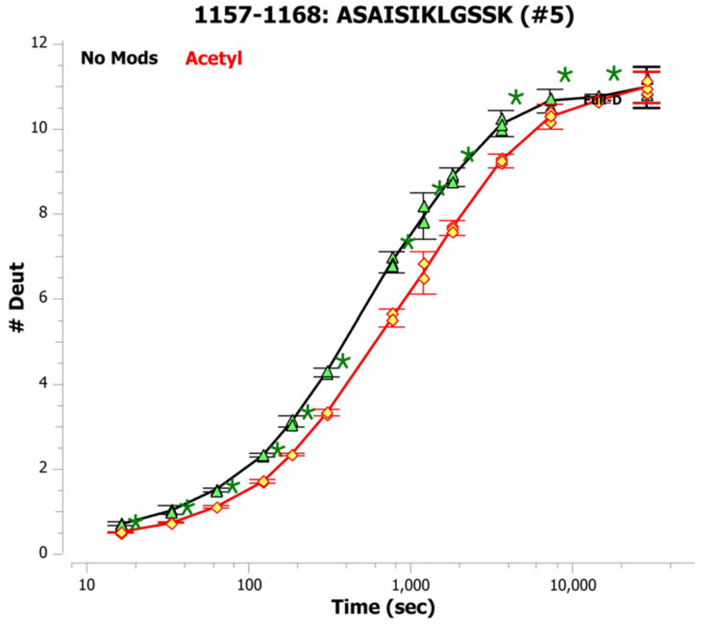
1157-1168: ASAISIKLGSSK (#5)
No Mods
Acetyl
Full-D
Deut
Time (sec)
0
2
4
6
8
10
12
10
100
1,000
10,000

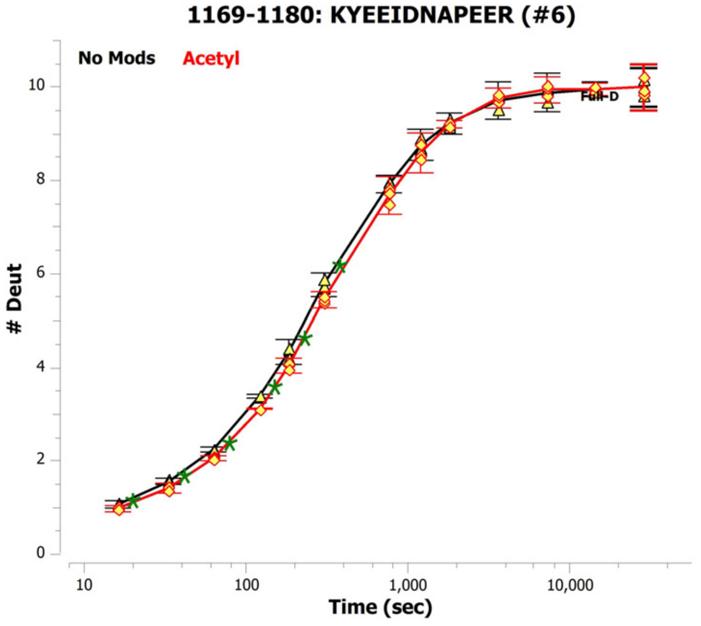
1169-1180: KYEEIDNAPEER (#6)
No Mods
Acetyl
Full-D
Deut
Time (sec)
0
2
4
6
8
10
10
100
1,000
10,000

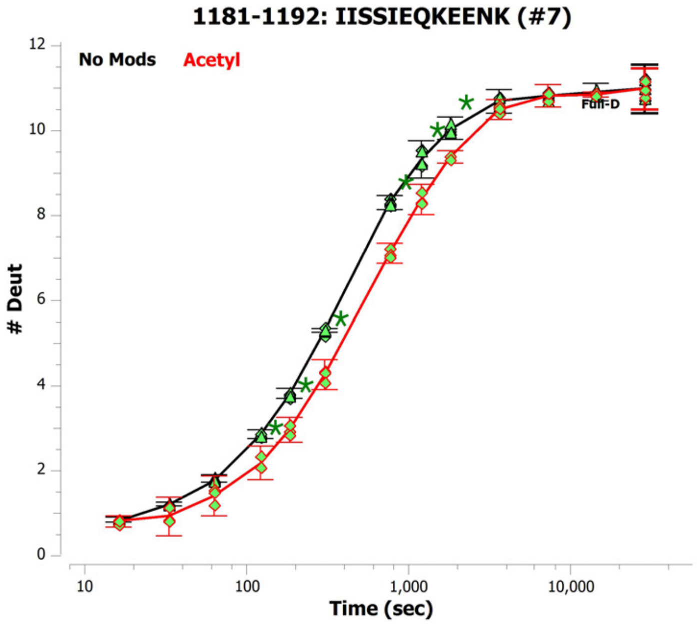
1181-1192: IISSIEQKEENK (#7)
No Mods
Acetyl
Full-D
Deut
Time (sec)

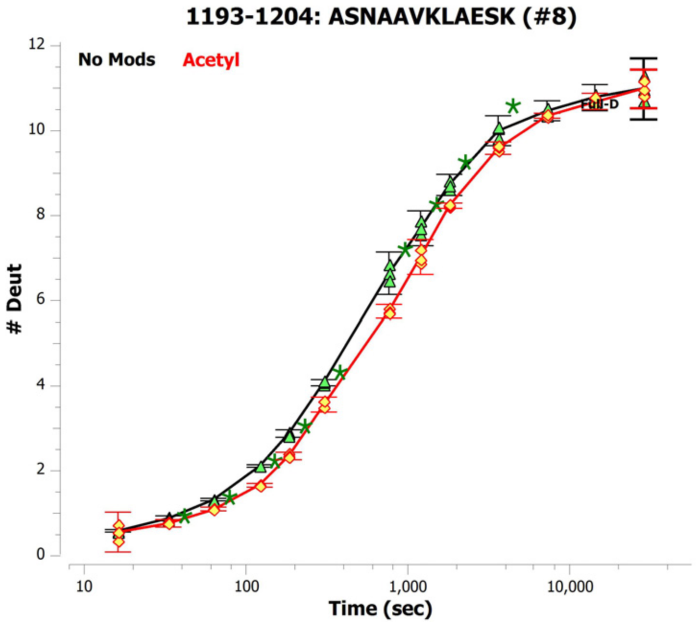
1193-1204: ASNAAVKLAESK (#8)
No Mods
Acetyl
Full-D
Deut
Time (sec)

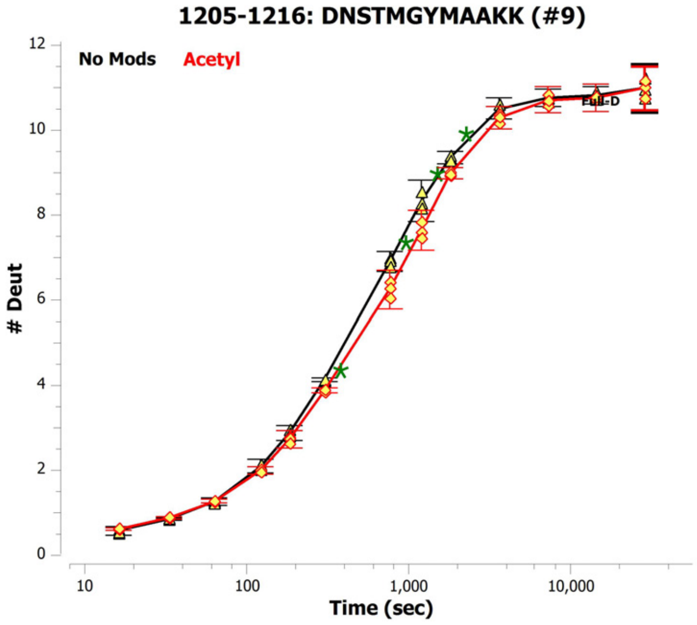
1205-1216: DNSTMGYMAAKK (#9)
No Mods
Acetyl
Full-D
Deut
Time (sec)

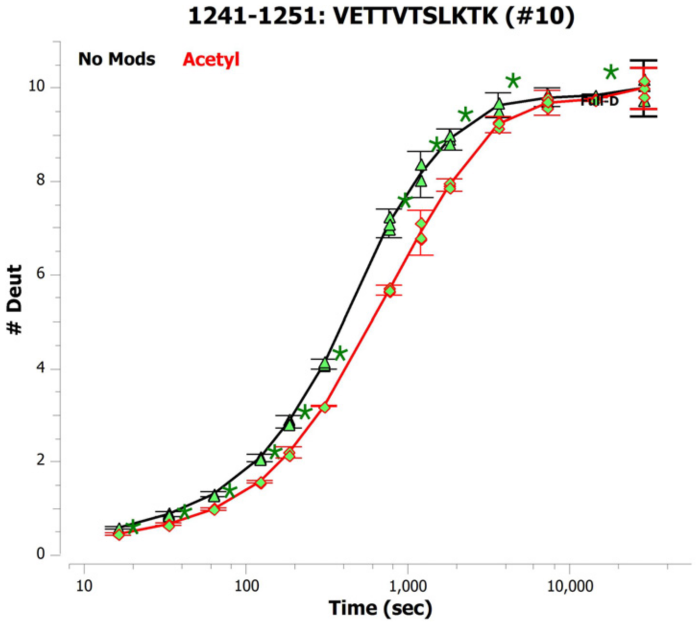
1241-1251: VETTVTSLKTK (#10)
No Mods
Acetyl
Full-D
Deut
Time (sec)

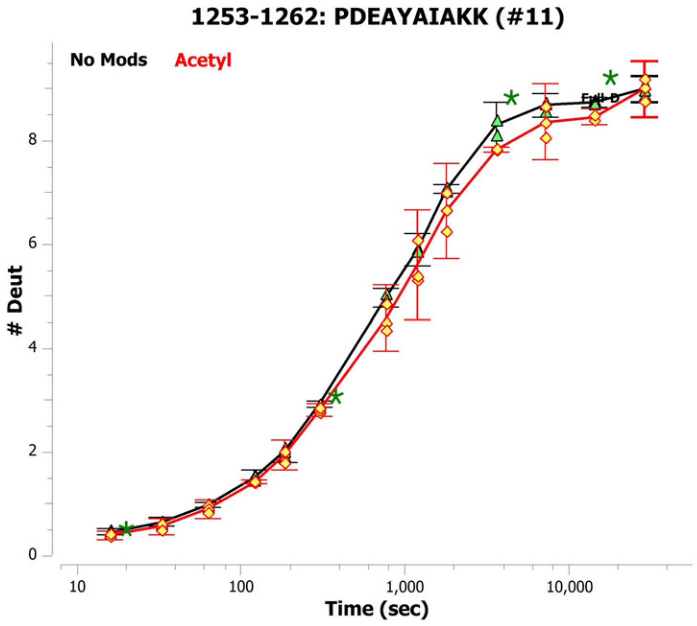
1253-1262: PDEAYAIAKK (#11)
No Mods
Acetyl
Full-D
Deut
Time (sec)

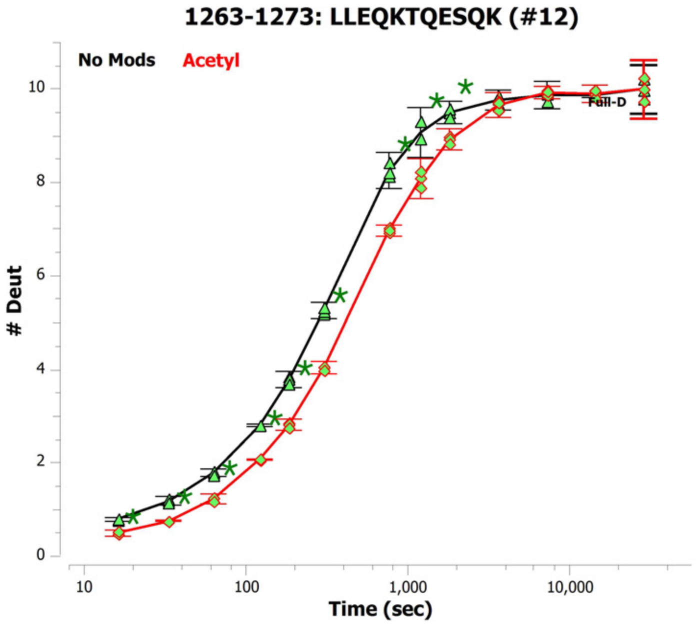
1263-1273: LLEQKTQESQK (#12)
No Mods
Acetyl
Full-D
Deut
Time (sec)

1274-1284: LEKTIDDLEEK (#13)

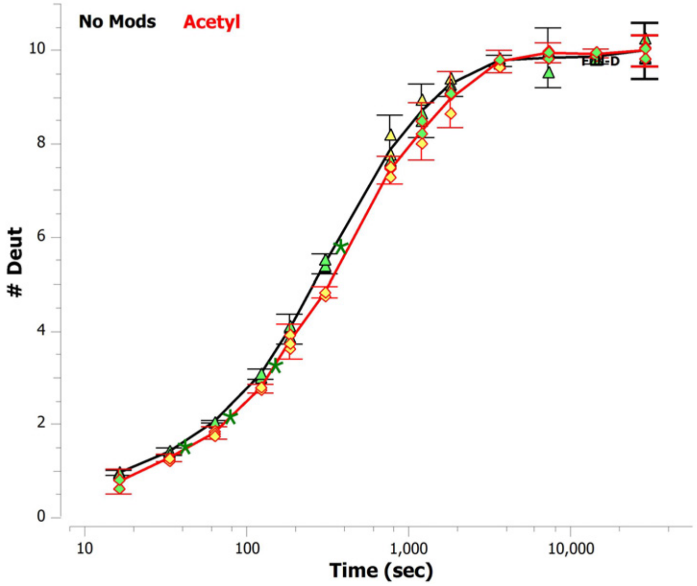

1285-1295: DFDTALKHYDK (#14)

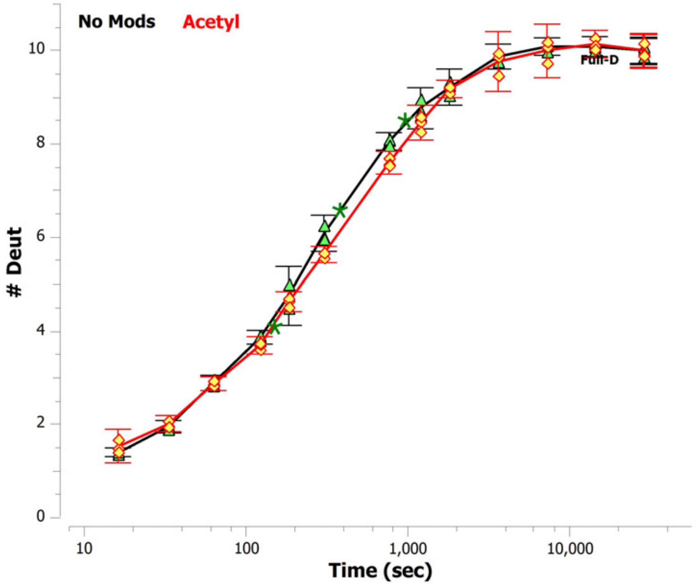

1296-1306: ITGKNQVTATK (#15)

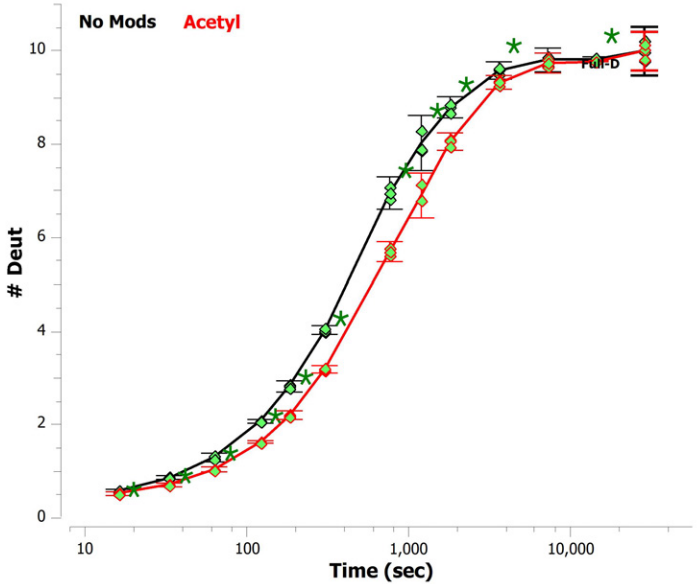

1297-1306: TGKNQVTATK (#16)

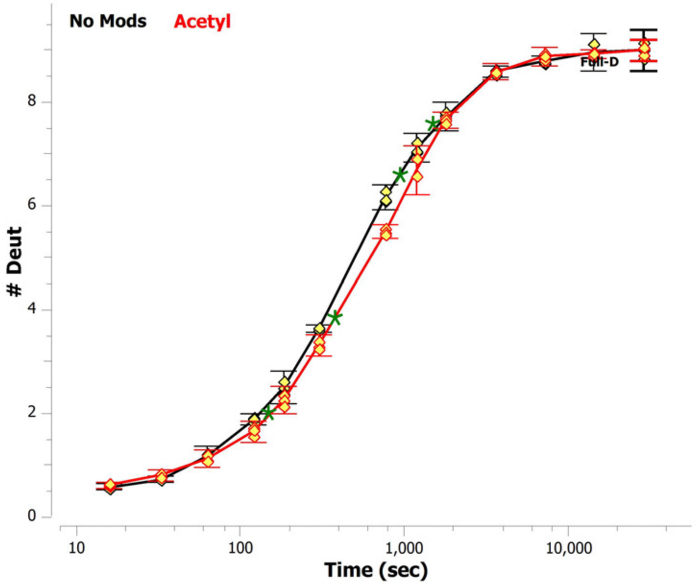

1307-1316: ASEALLKQLK (#17)

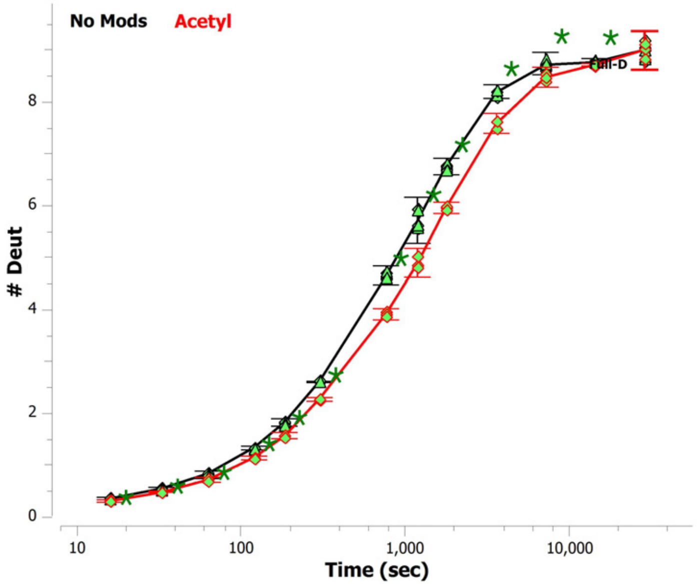

1317-1326: LLEEEDSKLK (#18)

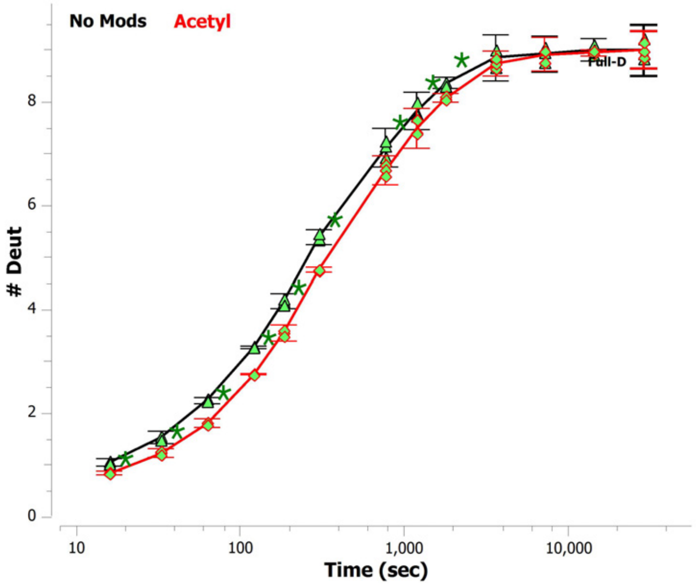

1327-1336: ISALQGKLSK (#19)

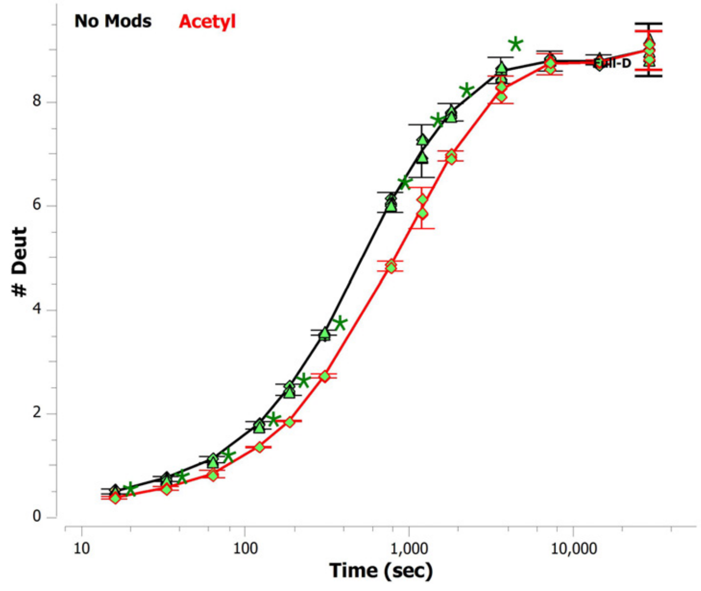

1337-1346: VNALKNLQVK (#20)

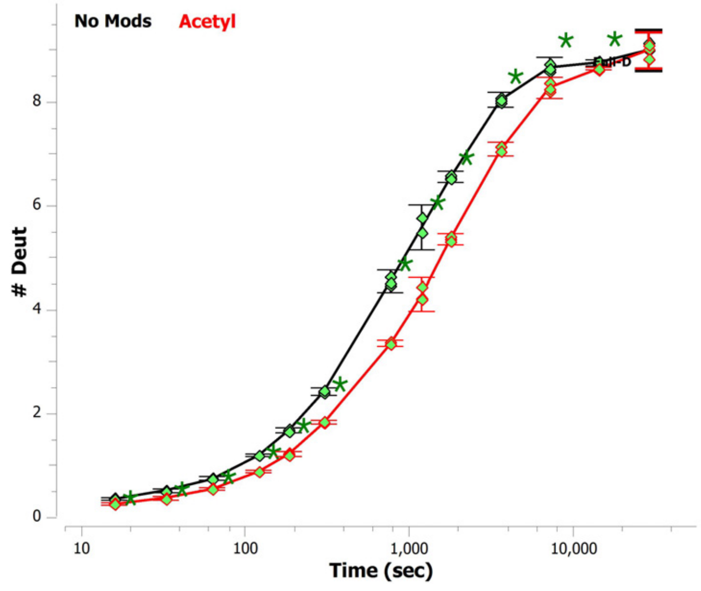

1347-1356: TPNLYIYSKK (#21)

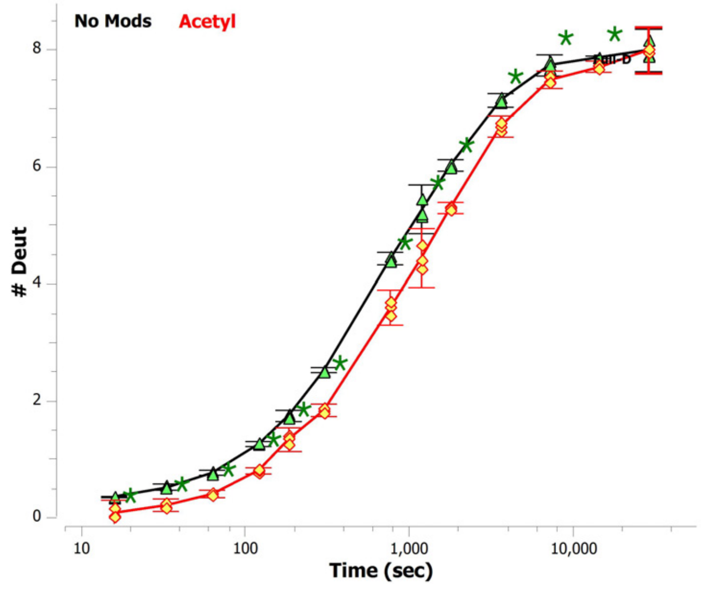

1357-1366: DQEALMKSVK (#22)

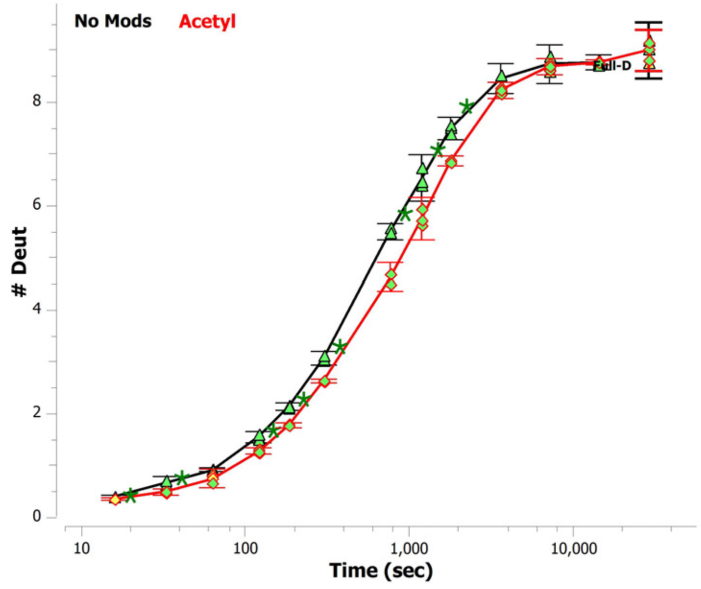

1367-1376: KFGVLSDNFK (#23)

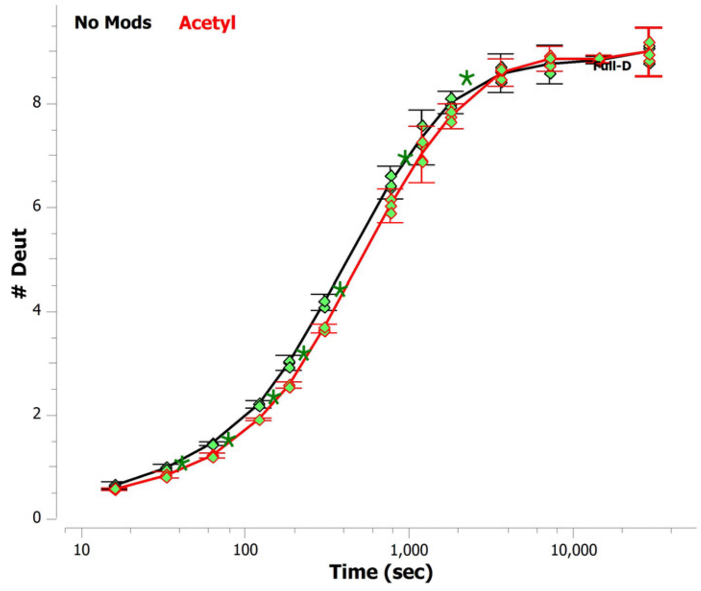

1377-1386: IQQELQTAKK (#24)

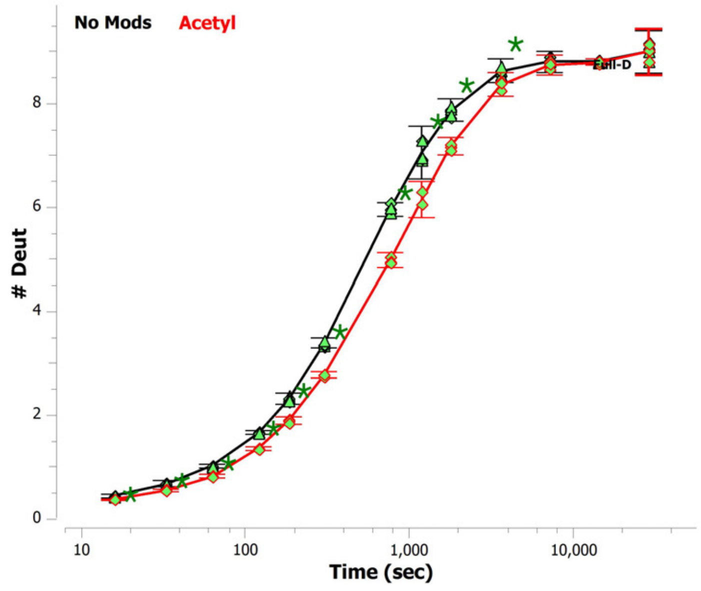

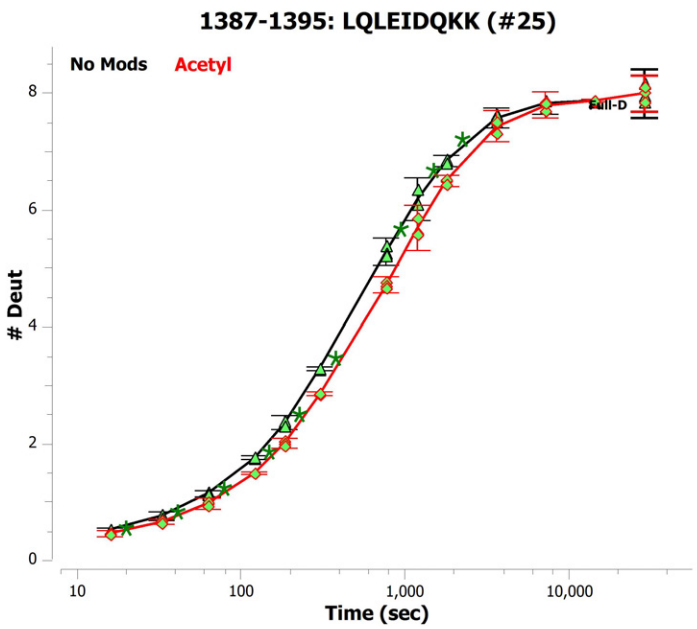

1387-1395: LQLEIDQKK (#25)
No Mods
Acetyl
Full-D
Deut
0
2
4
6
8
10
100
1,000
10,000
Time (sec)

Supplemental Figure 6A: D-uptake plots of non-modified and phosphopeptides (not normalized)

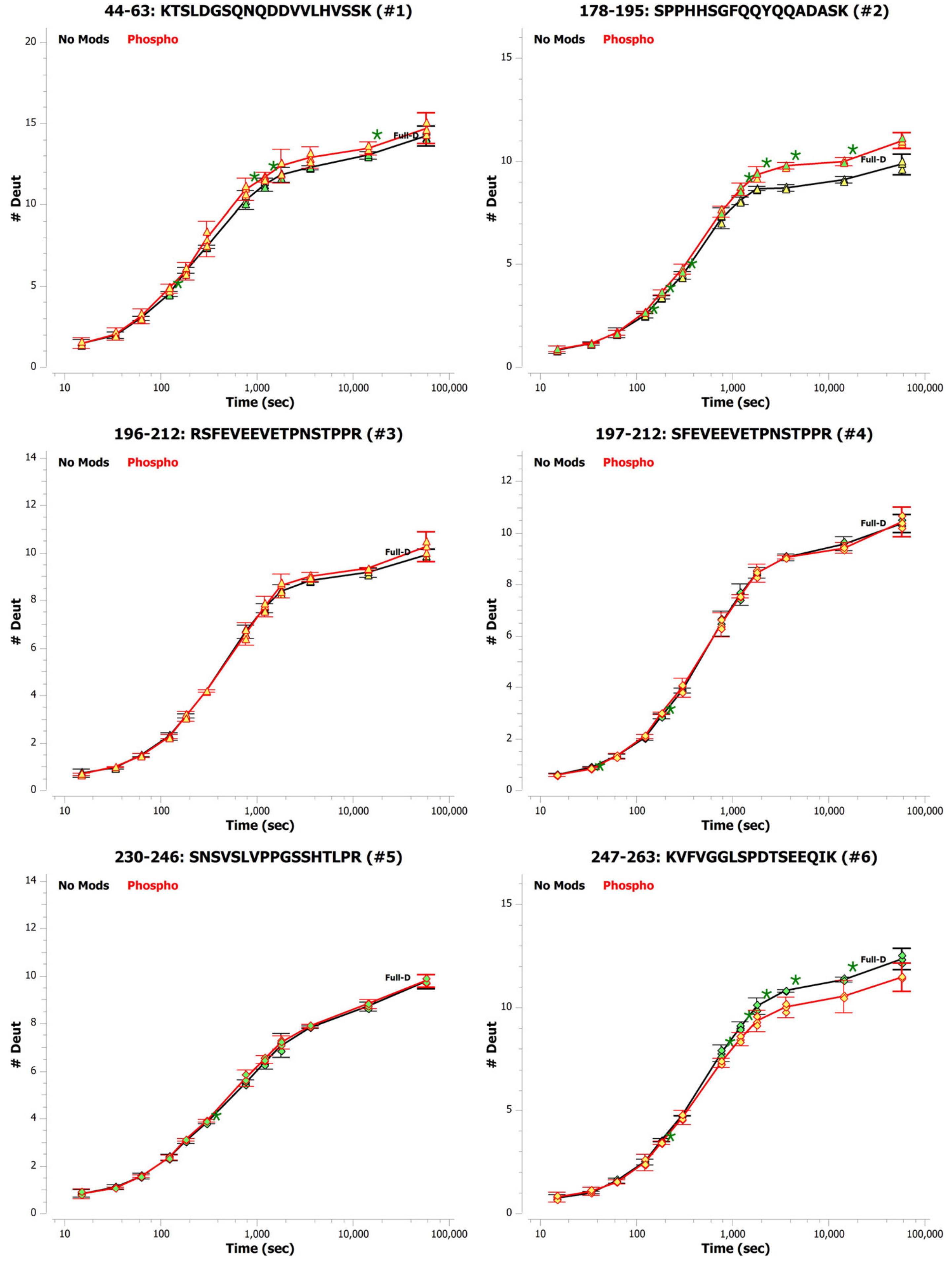

360-374: VHTGEKPYLCPECGK (#7)

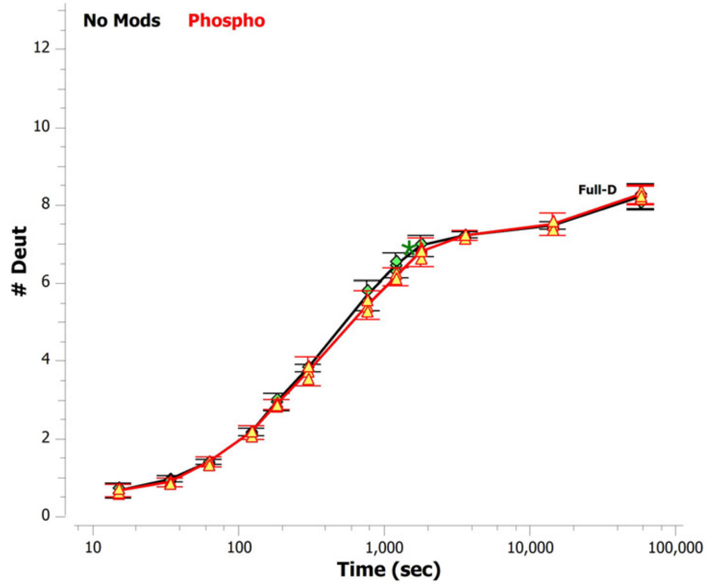

375-389: AHTGEKPYECNVCGK (#8)

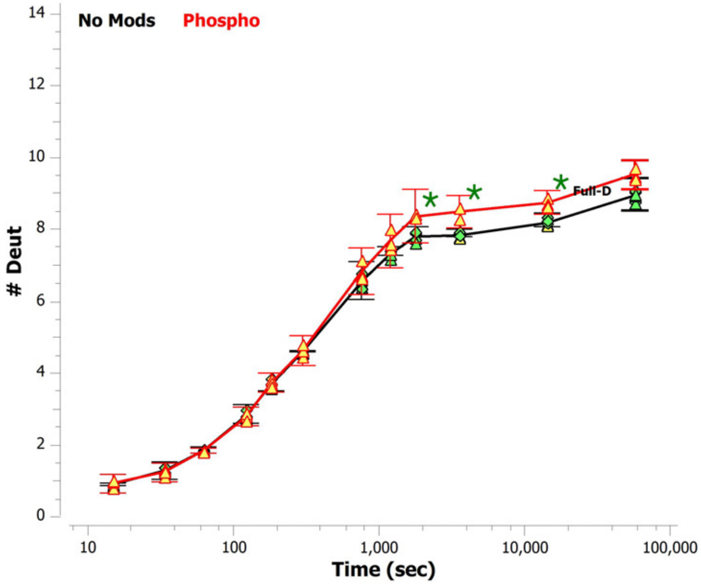

390-404: MSPALQDLSQPEGLK (#9)

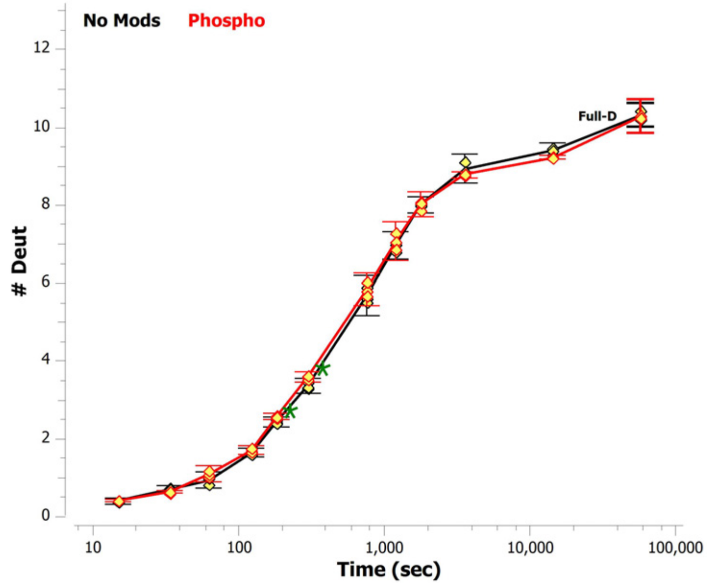

450-464: THTGEKPFECSQCGK (#10)

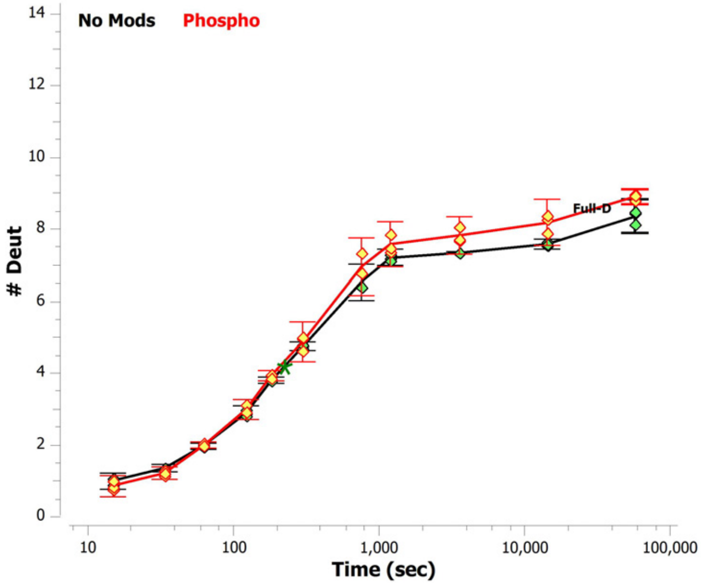

451-464: HTGEKPFECSQCGK (#11)

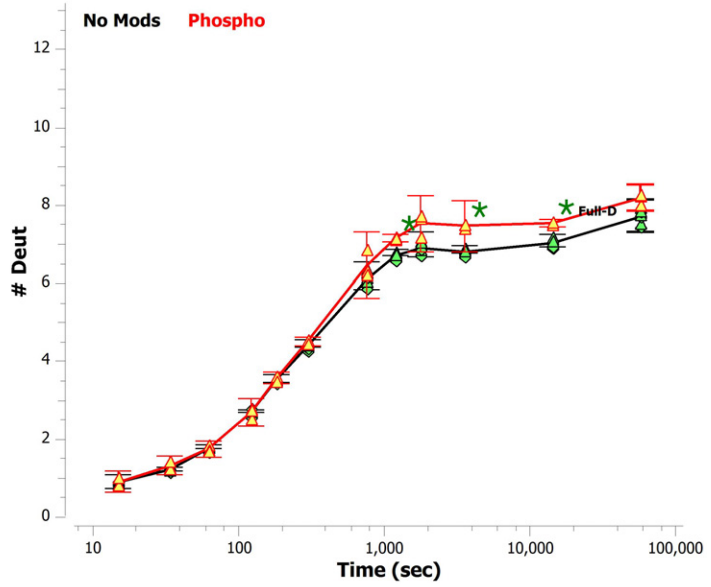

540-554: SGDSEVYQLGDVSQK (#12)

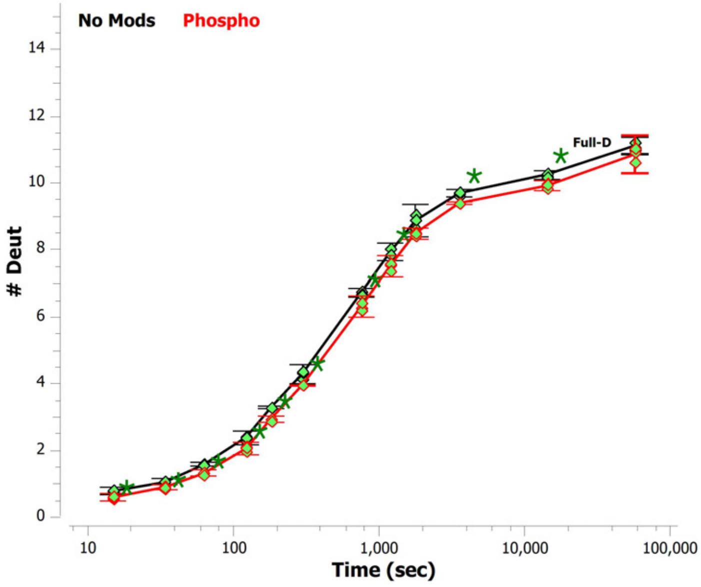

585-599: LGLSTLGELKQNLSR (#13)

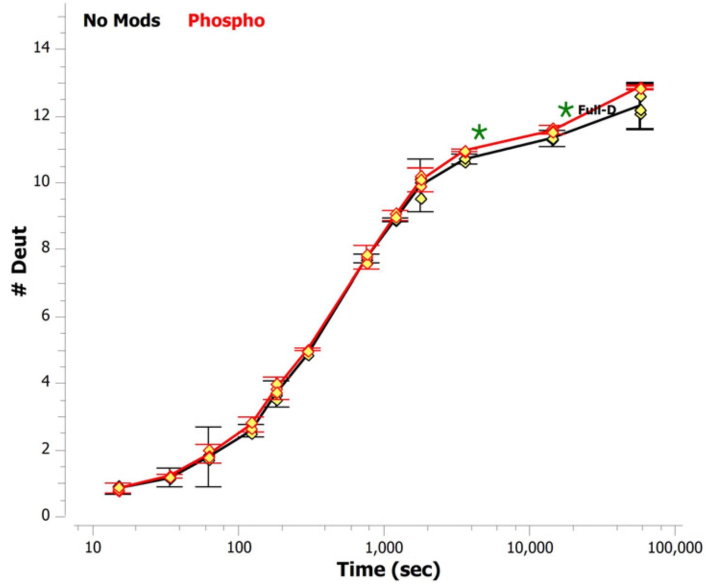

657-670: TSPPGGELSPGAGR (#14)

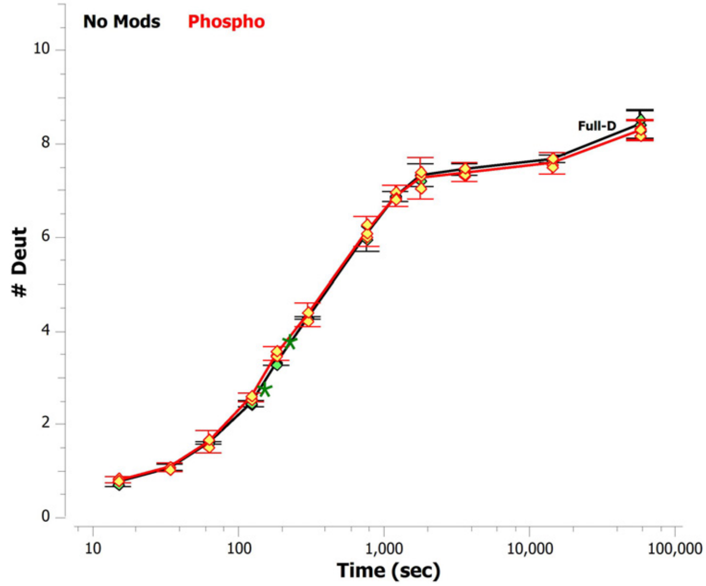

713-726: STHEFKPQSGAEIK (#15)

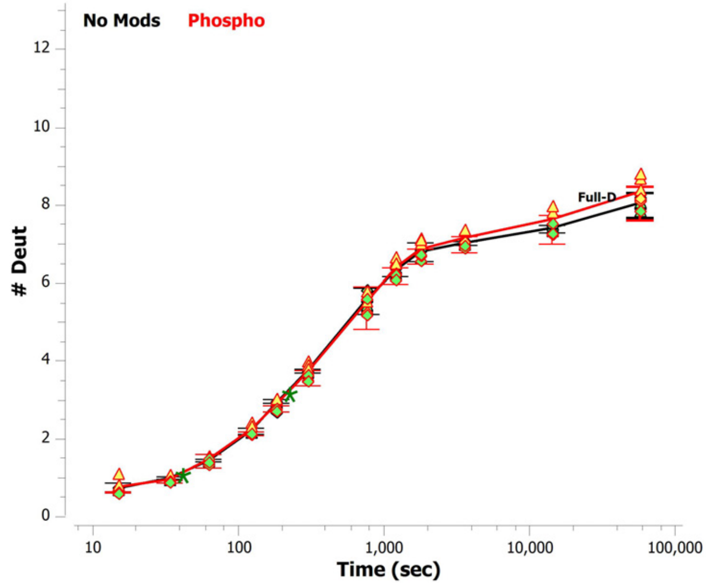

793-805: SKTSPVTQQPQQK (#16)

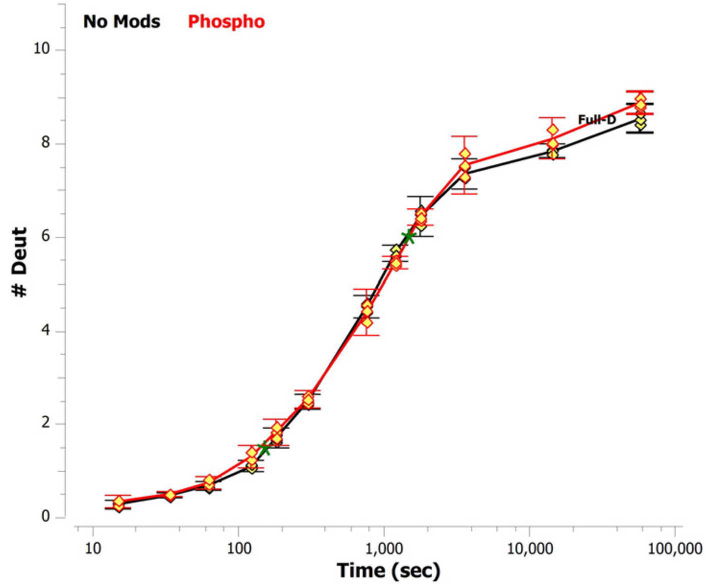

806-818: SLEFSYQEDKPTK (#17)

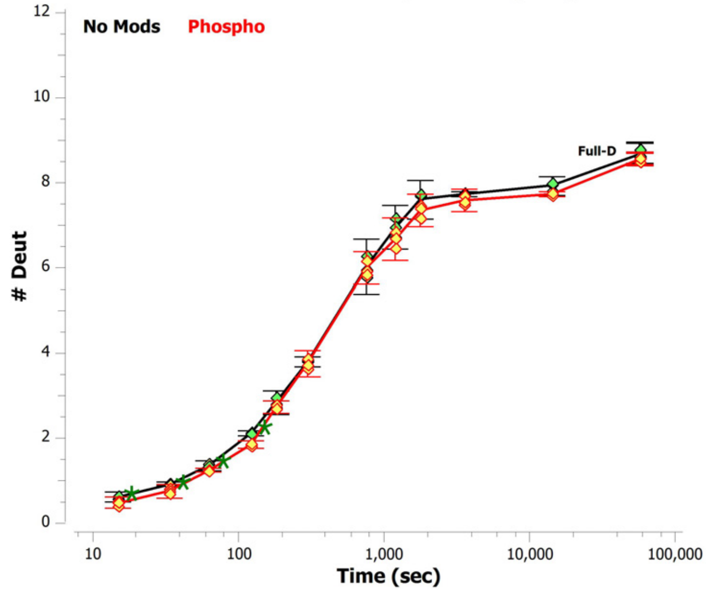

820-831: KTSQSEEEEAPR (#18)

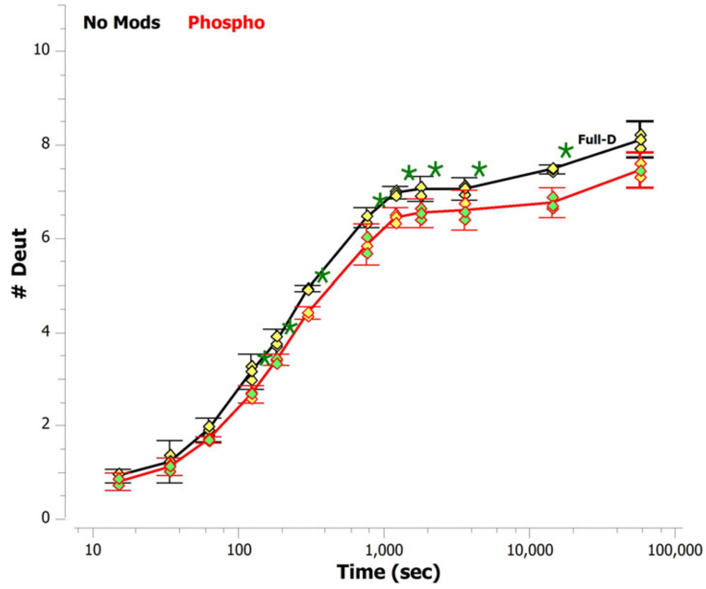

821-831: TSQSEEEEAPR (#19)

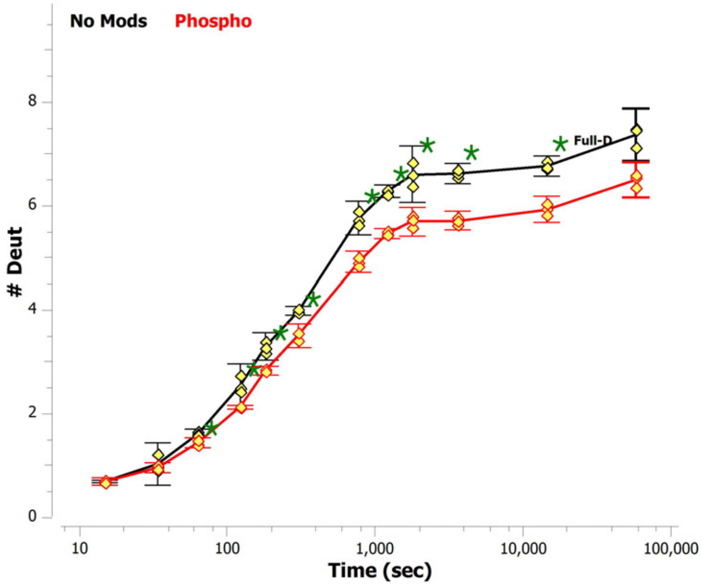

871-883: SNFSNSADDIKSK (#20)

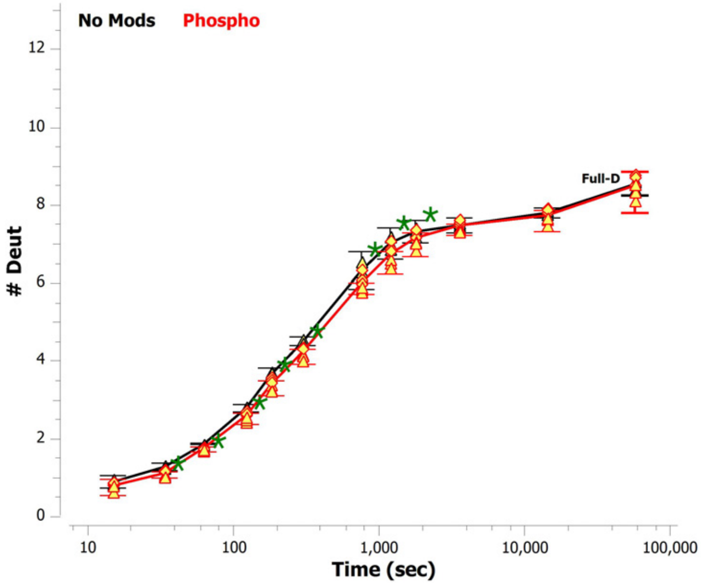

884-896: TATPQQAQEVHEK (#21)

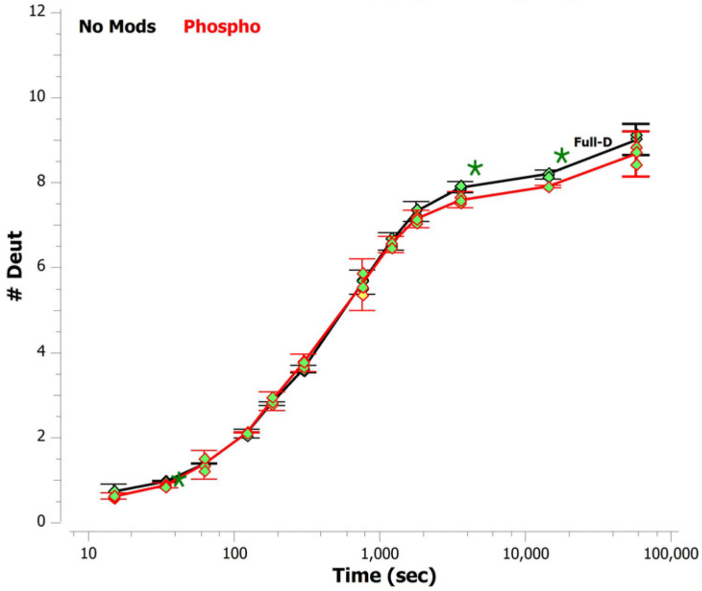

897-909: RSSENLSLDDCNK (#22)

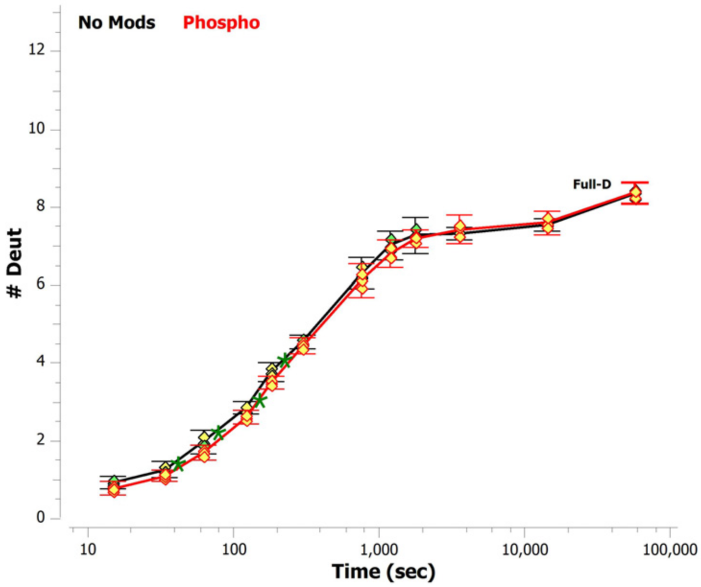

910-922: STTELGENLQELR (#23)

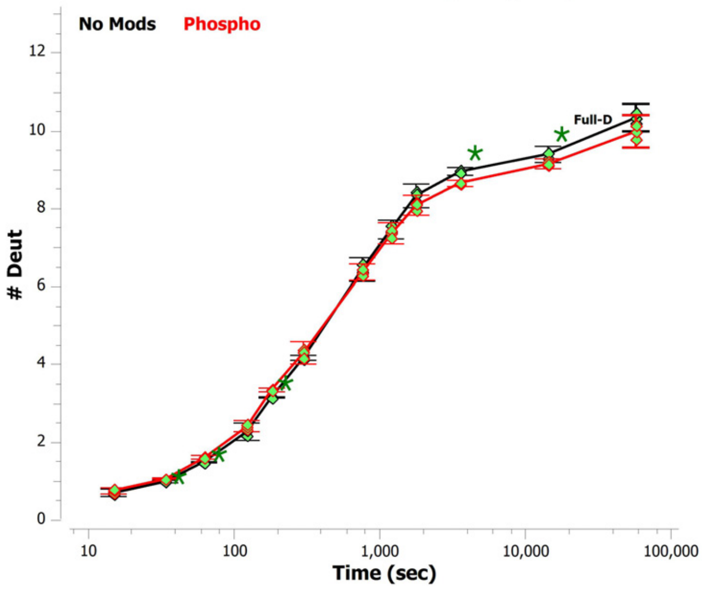

975-987: KTEELEEESFPER (#24)

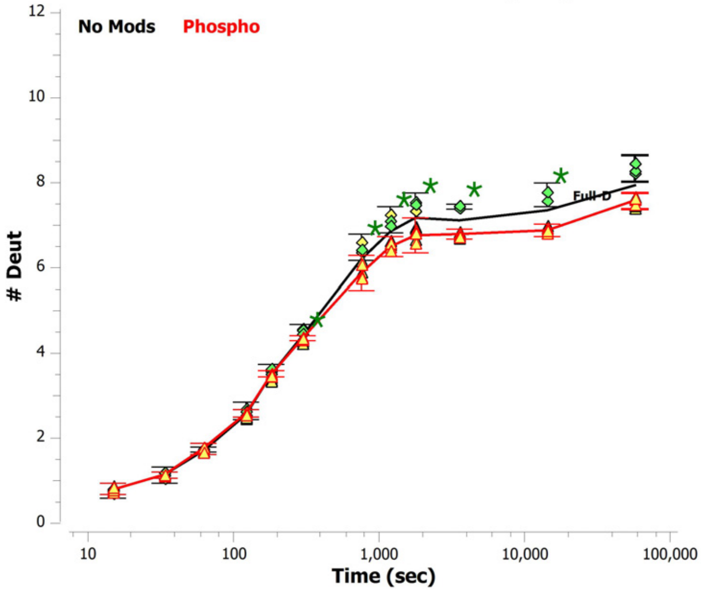

1084-1095: LTPSDMPLLELK (#25)

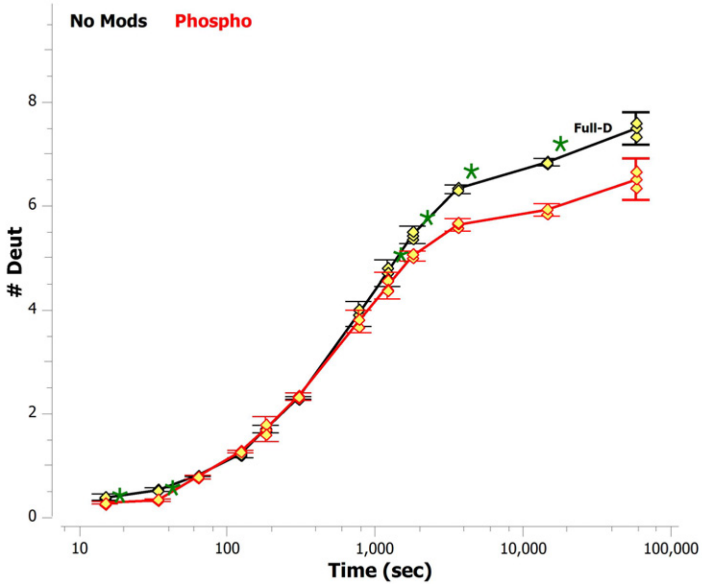

1180-1191: YTPEEEQELEKR (#26)

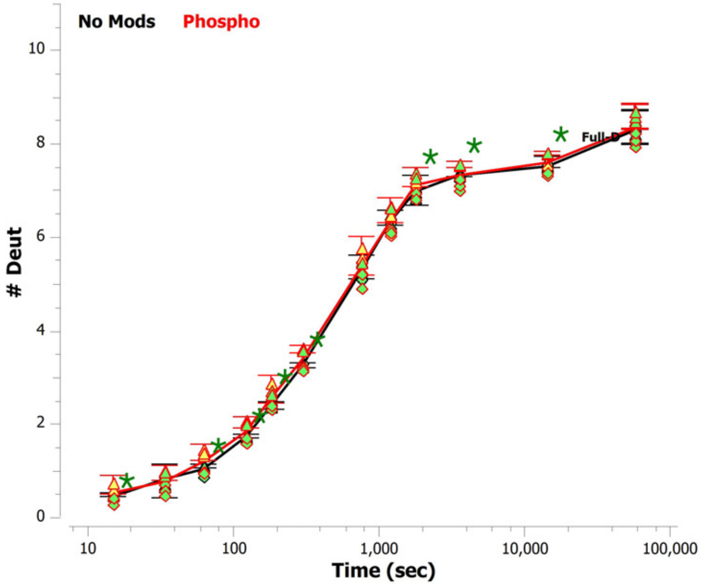

1192-1203: TSPVHPNLWASR (#27)

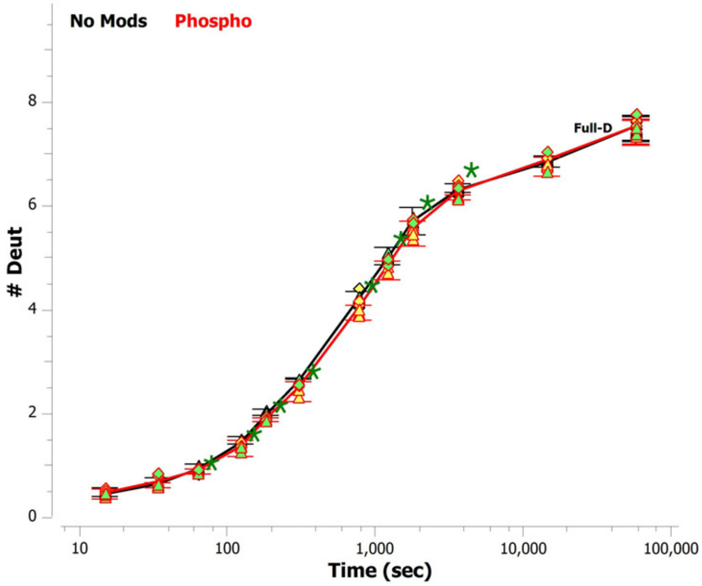

1216-1225: ASLSDIGFGK (#28)

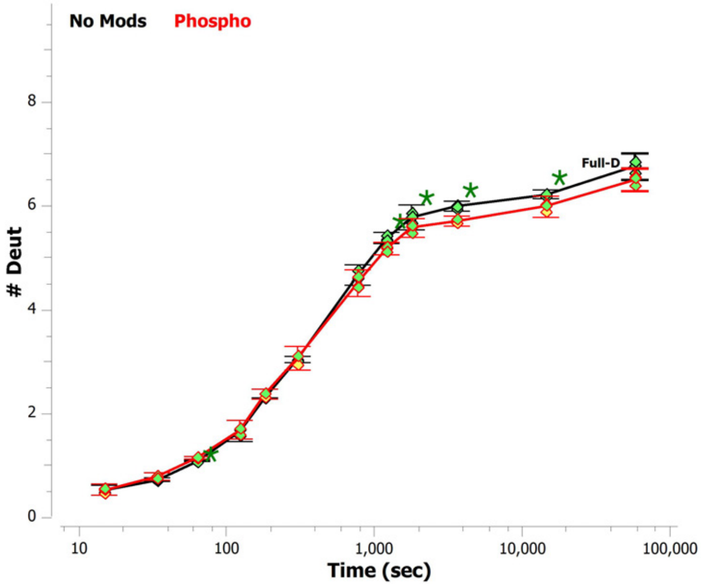

1305-1312: TPLLSFLK (#29)

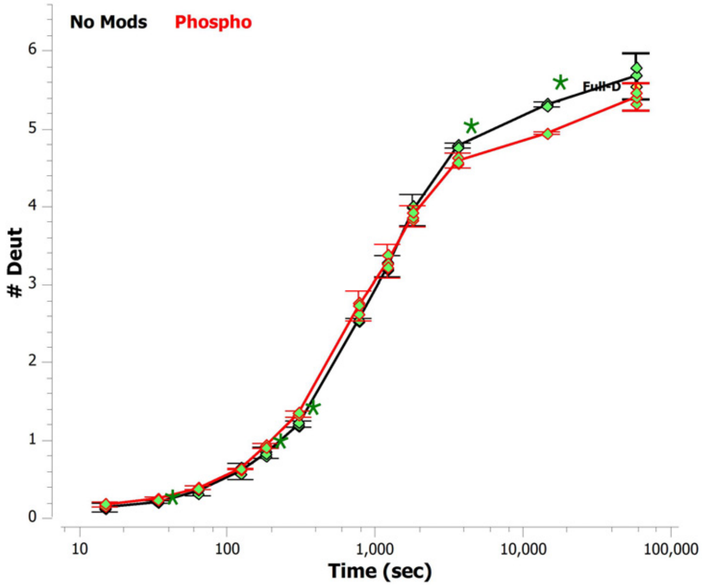

Supplemental Figure 6A: D-uptake plots of non-modified and phosphopeptides (FD normalized)

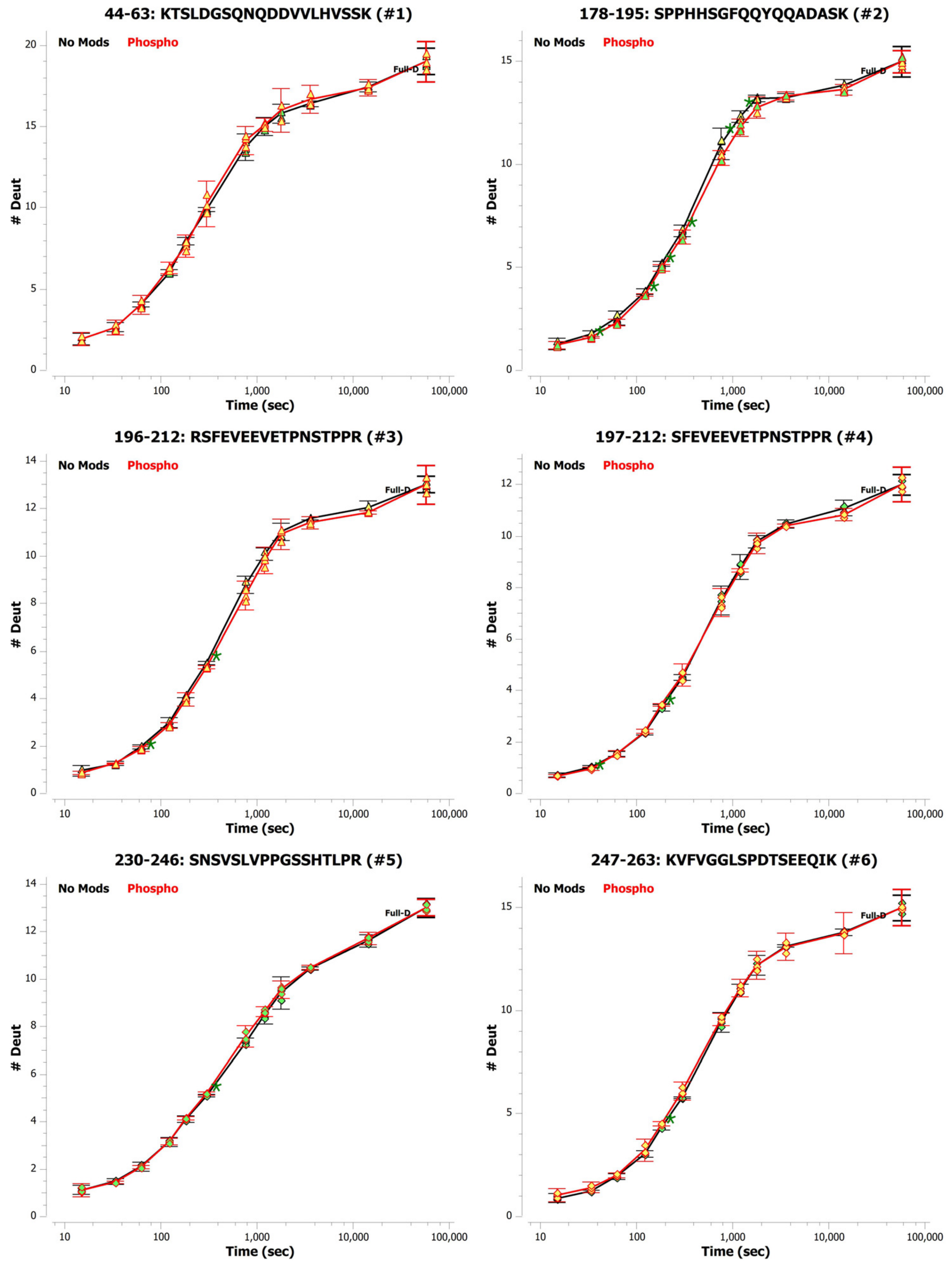

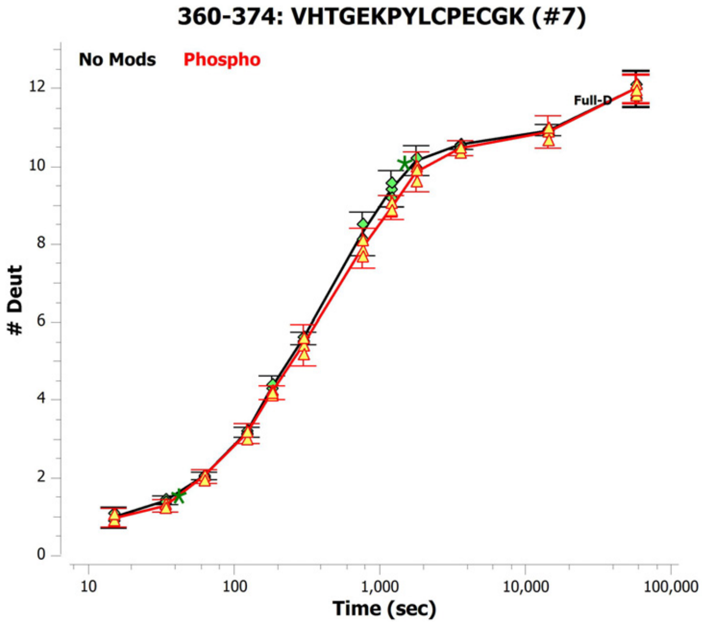
360-374: VHTGEKPYLCPECGK (#7)
No Mods
Phospho
Full-D
Deut
Time (sec)
10
100
1,000
10,000
100,000

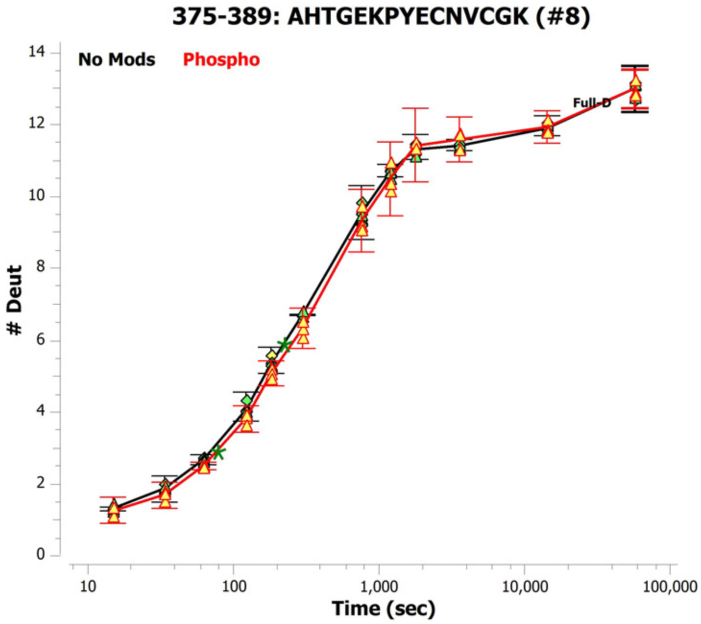
375-389: AHTGEKPYECNVCGK (#8)
No Mods
Phospho
Full-D
Deut
Time (sec)

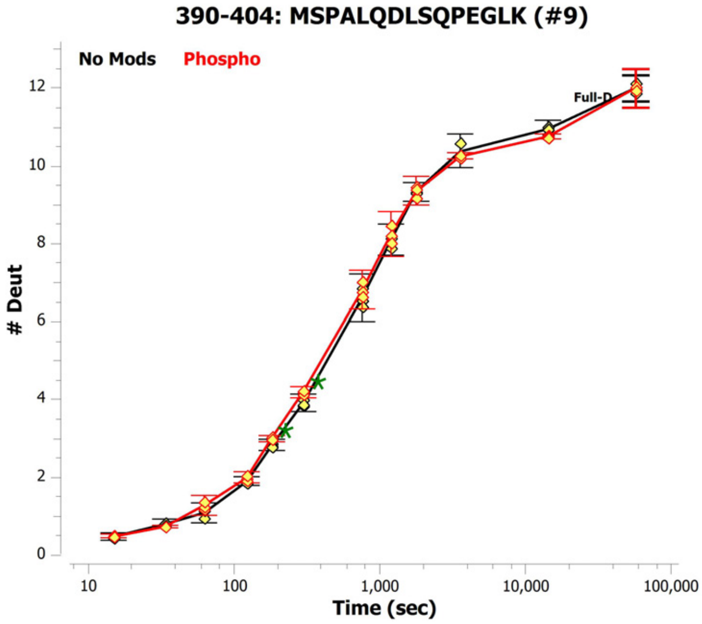
390-404: MSPALQDLSQPEGLK (#9)
No Mods
Phospho
Full-D
Deut
Time (sec)

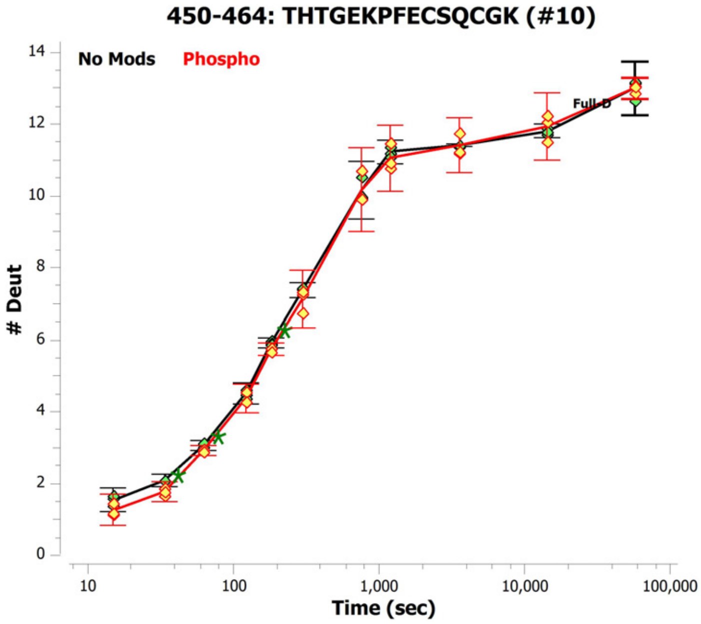
450-464: THTGEKPFECSQCGK (#10)
No Mods
Phospho
Full-D
Deut
Time (sec)

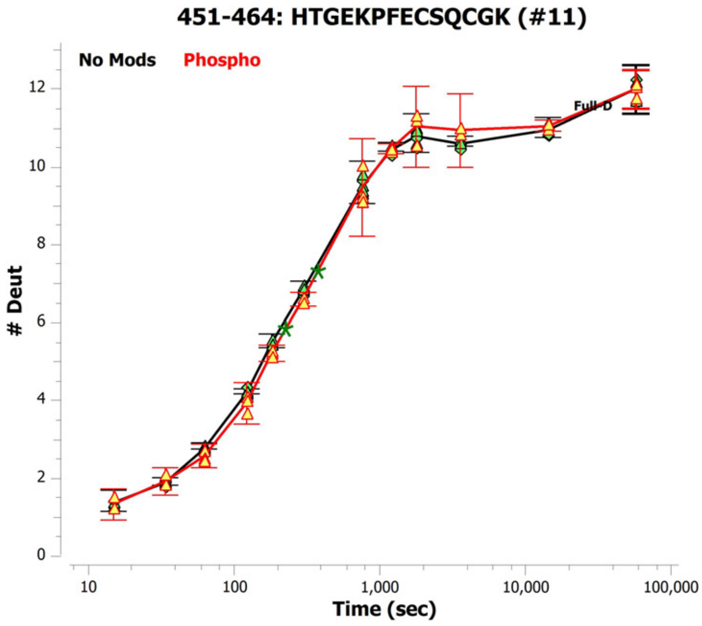
451-464: HTGEKPFECSQCGK (#11)
No Mods
Phospho
Full-D
Deut
Time (sec)

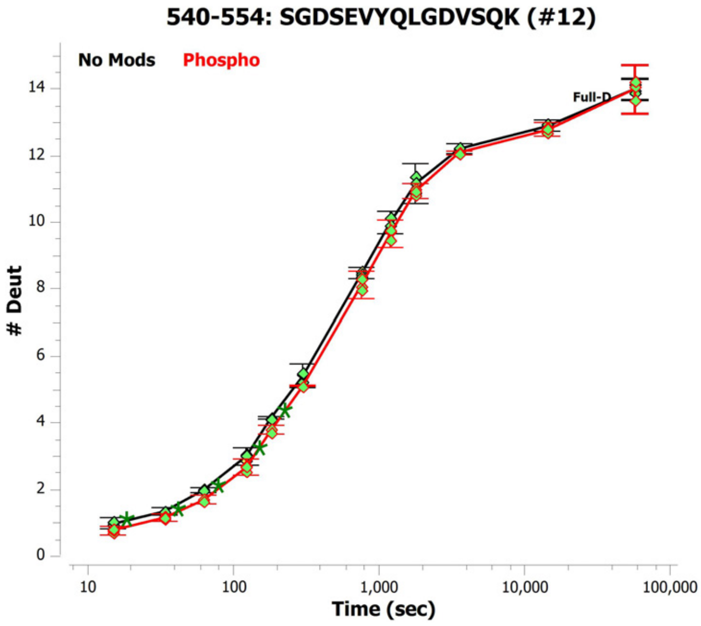
540-554: SGDSEVYQLGDVSQK (#12)
No Mods
Phospho
Full-D
Deut
Time (sec)

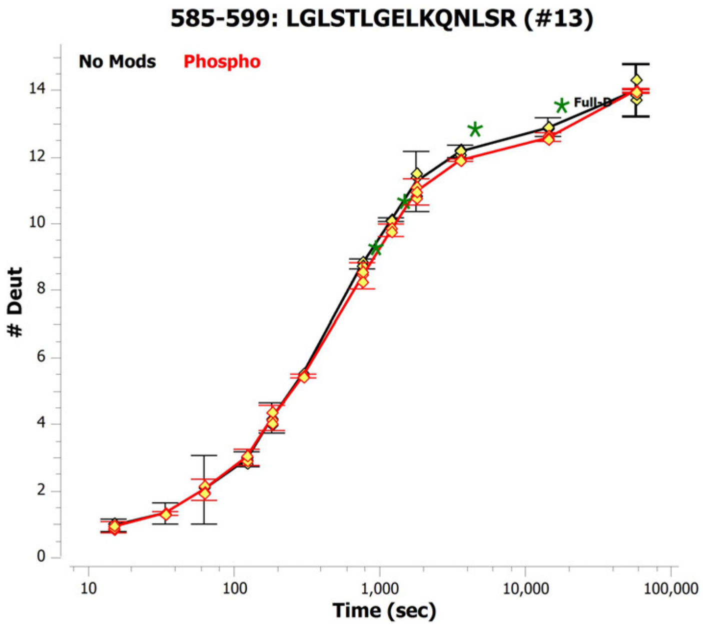
585-599: LGLSTLGELKQNLSR (#13)
No Mods
Phospho
Full-D
Deut
Time (sec)
0
2
4
6
8
10
12
14
10
100
1,000
10,000
100,000

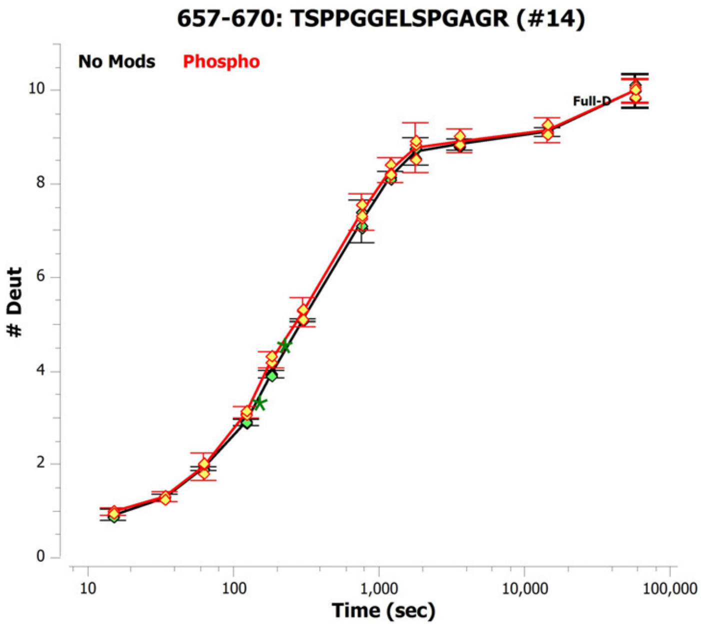
657-670: TSPPGGELSPGAGR (#14)
No Mods
Phospho
Full-D
Deut
Time (sec)
0
2
4
6
8
10
10
100
1,000
10,000
100,000

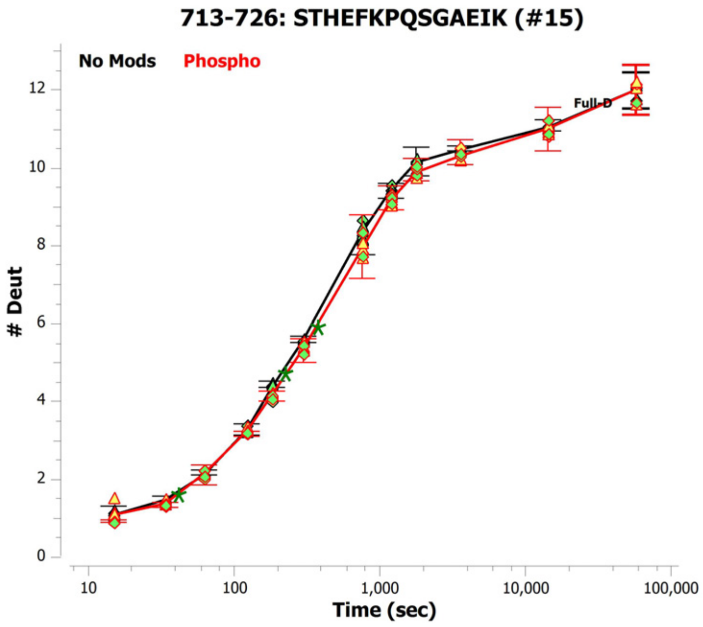
713-726: STHEFKPQSGAEIK (#15)
No Mods
Phospho
Full-D
Deut
Time (sec)
0
2
4
6
8
10
12
10
100
1,000
10,000
100,000

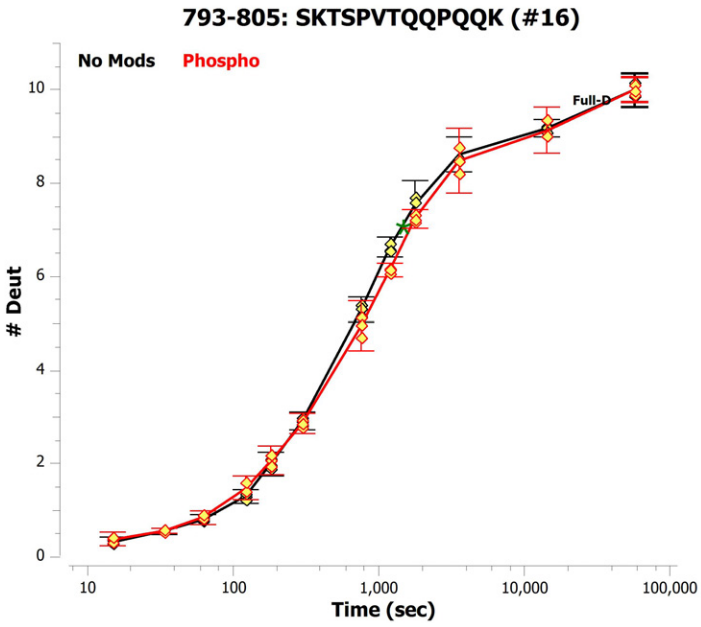
793-805: SKTSPVTQQPQQK (#16)
No Mods
Phospho
Full-D
Deut
Time (sec)
0
2
4
6
8
10
10
100
1,000
10,000
100,000

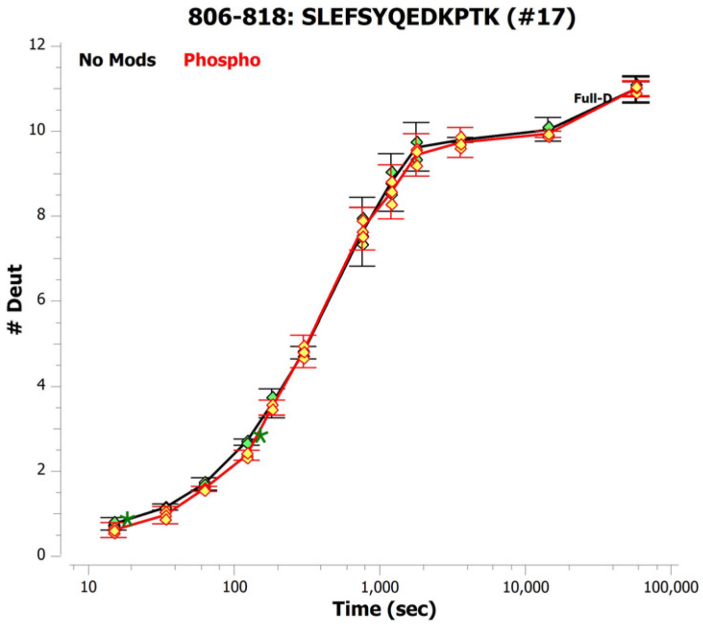
806-818: SLEFSYQEDKPTK (#17)
No Mods
Phospho
Full-D
Deut
Time (sec)
0
2
4
6
8
10
12
10
100
1,000
10,000
100,000

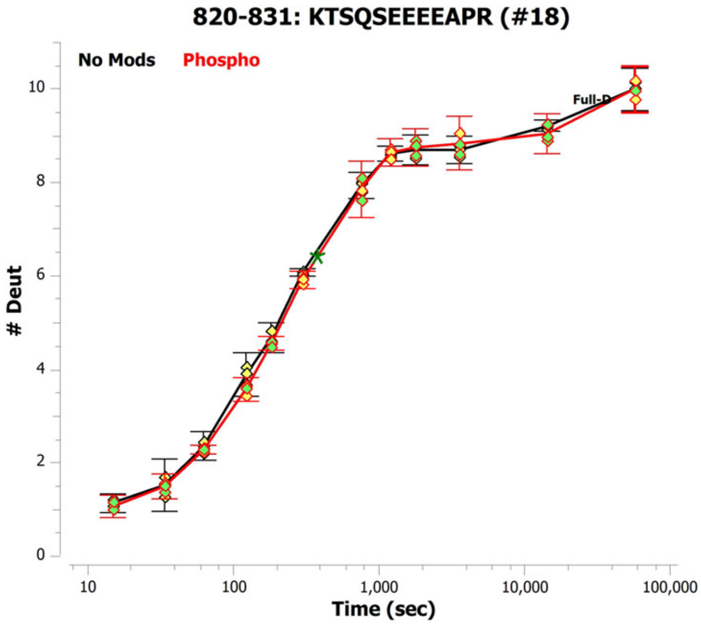
820-831: KTSQSEEEEAPR (#18)
No Mods
Phospho
Full-D
Deut
Time (sec)
0
2
4
6
8
10
10
100
1,000
10,000
100,000

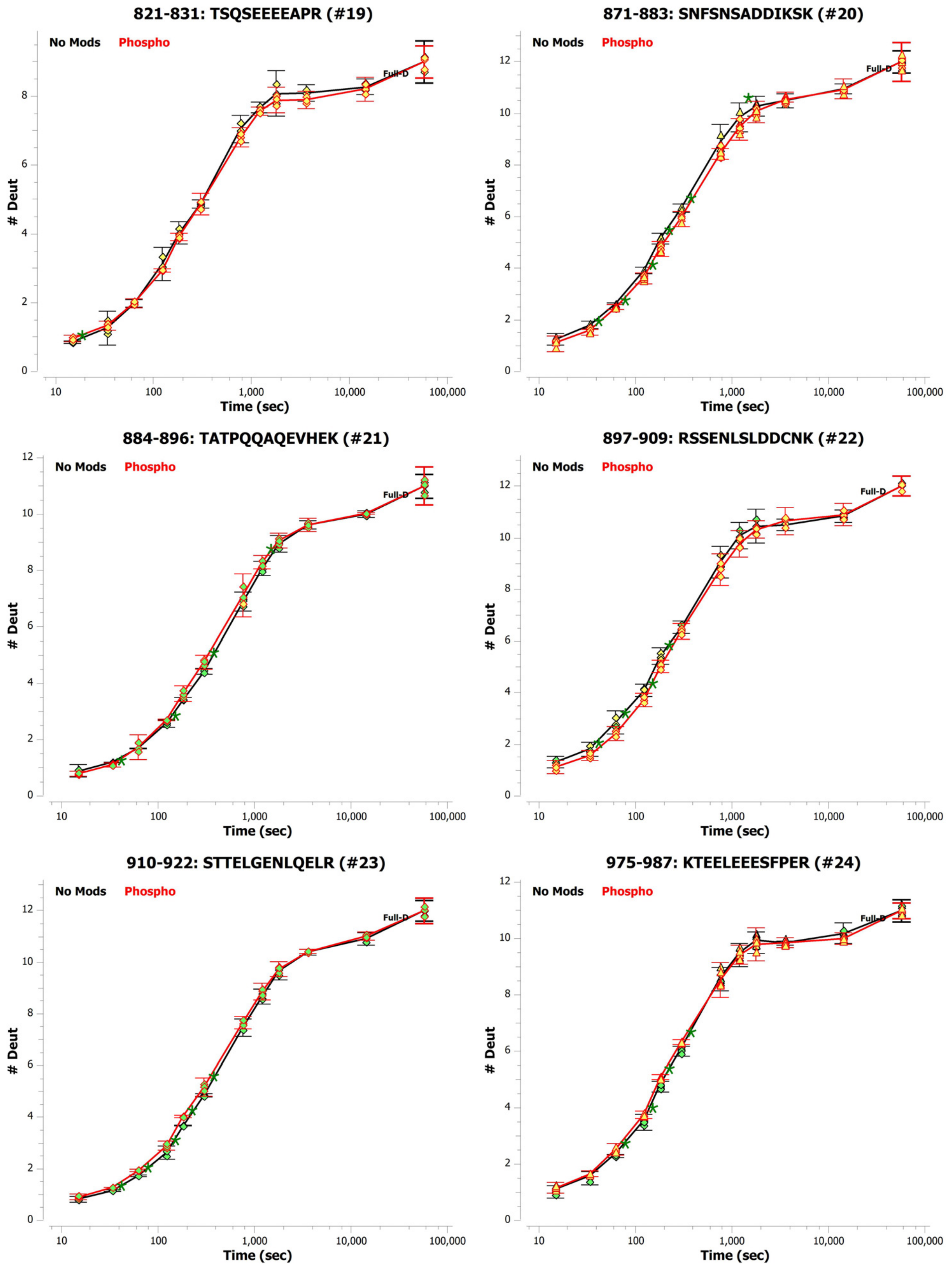
821-831: TSQSEEEEAPR (#19)
No Mods
Phospho
Full-D
Deut
Time (sec)
871-883: SNFSNSADDIKSK (#20)
No Mods
Phospho
Full-D
Deut
Time (sec)
884-896: TATPQQAQEVHEK (#21)
No Mods
Phospho
Full-D
Deut
Time (sec)
897-909: RSSENLSLDDCNK (#22)
No Mods
Phospho
Full-D
Deut
Time (sec)
910-922: STTELGENLQELR (#23)
No Mods
Phospho
Full-D
Deut
Time (sec)
975-987: KTEELEEESFPER (#24)
No Mods
Phospho
Full-D
Deut
Time (sec)

1084-1095: LTPSDMPLLELK (#25)

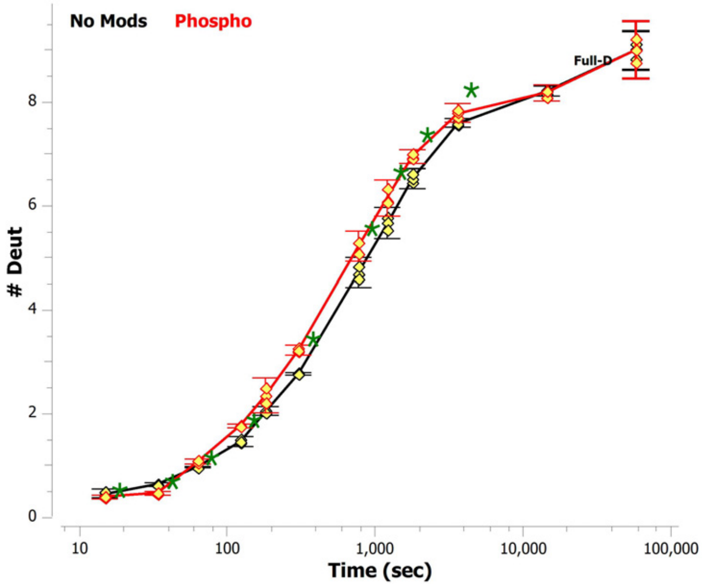

1180-1191: YTPEEEQELEKR (#26)

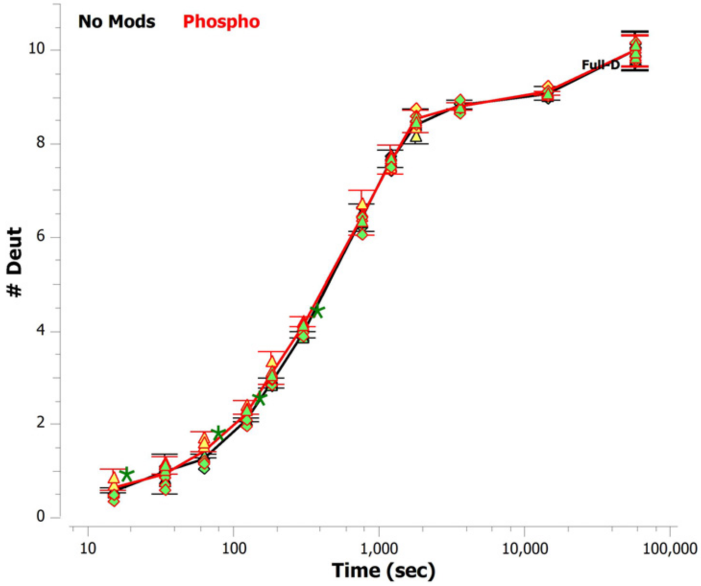

1192-1203: TSPVHPNLWASR (#27)

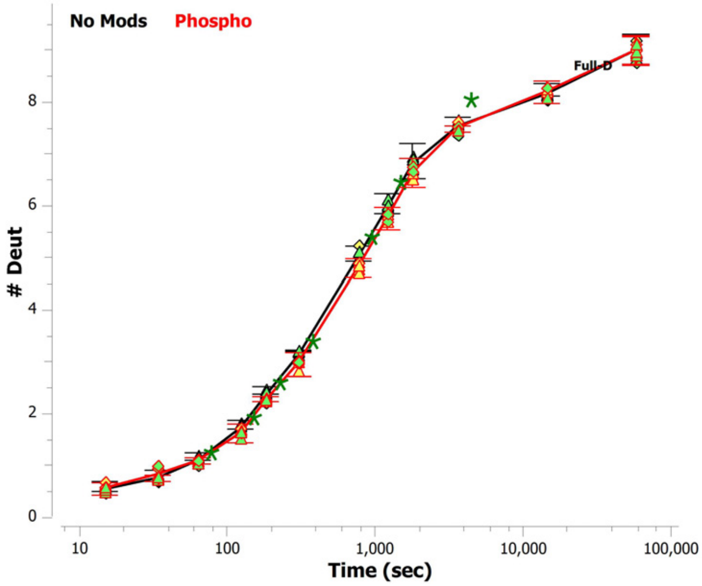

1216-1225: ASLSDIGFGK (#28)

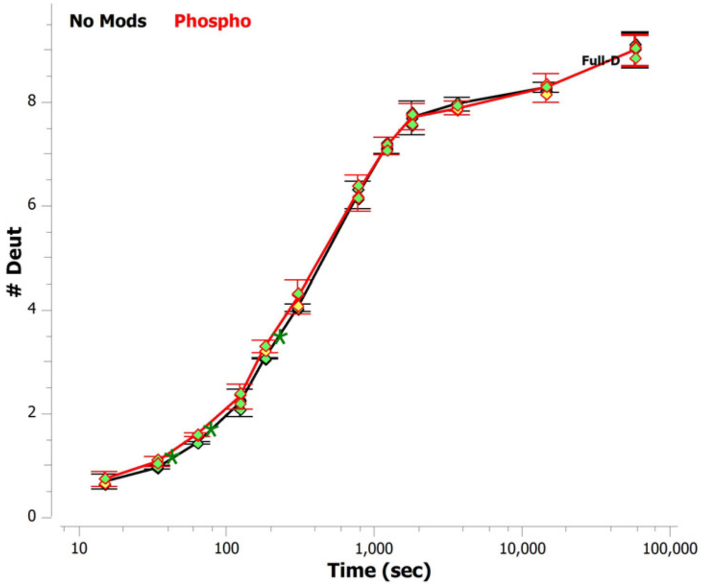

1305-1312: TPLLSFLK (#29)

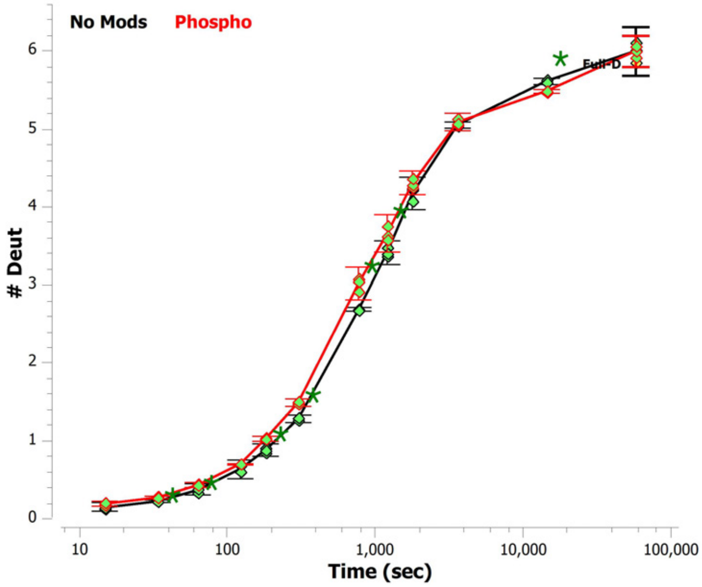

Supplement: Supplemental data [file mmc1.pdf]
